# Ambroxol for the Treatment of Patients With Parkinson Disease With and Without Glucocerebrosidase Gene Mutations: A Nonrandomized, Noncontrolled Trial

**DOI:** 10.1001/jamaneurol.2019.4611

**Published:** 2020-01-13

**Authors:** Stephen Mullin, Laura Smith, Katherine Lee, Gayle D’Souza, Philip Woodgate, Josh Elflein, Jenny Hällqvist, Marco Toffoli, Adam Streeter, Joanne Hosking, Wendy E. Heywood, Rajeshree Khengar, Philip Campbell, Jason Hehir, Sarah Cable, Kevin Mills, Henrik Zetterberg, Patricia Limousin, Vincenzo Libri, Tom Foltynie, Anthony H. V. Schapira

**Affiliations:** 1 Department of Clinical and Movement Neurosciences, University College London Institute of Neurology, London, United Kingdom; 2 Institute of Translational and Stratified Medicine, University of Plymouth School of Medicine, Plymouth, United Kingdom; 3 NIHR UCLH Clinical Research Facility, University College London Hospitals NHS Foundation Trust, London, United Kingdom; 4 Translational Mass Spectrometry Research Group, University College London Institute of Child Health, London, United Kingdom; 5 Department of Medical Statistics, University of Plymouth School of Medicine, Plymouth, United Kingdom; 6 Neurogenetics Unit, National Hospital for Neurology and Neurosurgery, UCLH NHS Foundation Trust, London, United Kingdom; 7 Department of Neurodegenerative Disease, University College London Institute of Neurology, London, United Kingdom; 8 UK Dementia Research Institute at University College London, London, United Kingdom; 9 Clinical Neurochemistry Laboratory, Sahlgrenska University Hospital, Molndal, Sweden; 10 Institute of Neuroscience and Physiology, Department of Psychiatry and Neurochemistry, Sahlgrenska Academy, University of Gothenburg, Molndal, Sweden

## Abstract

**Importance:**

Mutations of the glucocerebrosidase gene, *GBA1* (OMIM 606463), are the most important risk factor for Parkinson disease (PD). In vitro and in vivo studies have reported that ambroxol increases β-glucocerebrosidase (GCase) enzyme activity and reduces α-synuclein levels. These observations support a potential role for ambroxol therapy in modifying a relevant pathogenetic pathway in PD.

**Objective:**

To assess safety, tolerability, cerebrospinal fluid (CSF) penetration, and target engagement of ambroxol therapy with GCase in patients with PD with and without *GBA1* mutations.

**Interventions:**

An escalating dose of oral ambroxol to 1.26 g per day.

**Design, Setting, and Participants:**

This single-center open-label noncontrolled clinical trial was conducted between January 11, 2017, and April 25, 2018, at the Leonard Wolfson Experimental Neuroscience Centre, a dedicated clinical research facility and part of the University College London Queen Square Institute of Neurology in London, United Kingdom. Participants were recruited from established databases at the Royal Free London Hospital and National Hospital for Neurology and Neurosurgery in London. Twenty-four patients with moderate PD were evaluated for eligibility, and 23 entered the study. Of those, 18 patients completed the study; 1 patient was excluded (failed lumbar puncture), and 4 patients withdrew (predominantly lumbar puncture–related complications). All data analyses were performed from November 1 to December 14, 2018.

**Main Outcomes and Measures:**

Primary outcomes at 186 days were the detection of ambroxol in the CSF and a change in CSF GCase activity.

**Results:**

Of the 18 participants (15 men [83.3%]; mean [SD] age, 60.2 [9.7] years) who completed the study, 17 (8 with *GBA1* mutations and 9 without *GBA1* mutations) were included in the primary analysis. Between days 0 and 186, a 156-ng/mL increase in the level of ambroxol in CSF (lower 95% confidence limit, 129 ng/mL; *P* < .001) was observed. The CSF GCase activity decreased by 19% (0.059 nmol/mL per hour; 95% CI, –0.115 to –0.002; *P* = .04). The ambroxol therapy was well tolerated, with no serious adverse events. An increase of 50 pg/mL (13%) in the CSF α-synuclein concentration (95% CI, 14-87; *P* = .01) and an increase of 88 ng/mol (35%) in the CSF GCase protein levels (95% CI, 40-137; *P* = .002) were observed. Mean (SD) scores on part 3 of the Movement Disorders Society Unified Parkinson Disease Rating Scale decreased (ie, improved) by 6.8 (7.1) points (95% CI, –10.4 to –3.1; *P* = .001). These changes were observed in patients with and without *GBA1* mutations.

**Conclusions and Relevance:**

The study results suggest that ambroxol therapy was safe and well tolerated; CSF penetration and target engagement of ambroxol were achieved, and CSF α-synuclein levels were increased. Placebo-controlled clinical trials are needed to examine whether ambroxol therapy is associated with changes in the natural progression of PD.

**Trial Registration:**

ClinicalTrials.gov identifier: NCT02941822; EudraCT identifier: 2015-002571-24

## Key Points

**Question:**

Does ambroxol cross the blood-brain barrier, and what are the biochemical changes associated with ambroxol therapy in patients with Parkinson disease with and without glucocerebrosidase gene mutations?

**Findings:**

In this open-label clinical trial of 17 patients with Parkinson disease, ambroxol crossed the blood-brain barrier and bound to the β-glucocerebrosidase enzyme, and it increased β-glucocerebrosidase enzyme protein levels and cerebrospinal fluid α-synuclein levels in patients both with and without glucocerebrosidase gene mutations.

**Meaning:**

Ambroxol therapy has potential for study as a neuroprotective compound for the treatment of patients with Parkinson disease both with and without glucocerebrosidase gene mutations.

## Teaser

This single-center open-label nonrandomized, noncontrolled trial assesses the safety, tolerability, cerebrospinal fluid penetration, and biochemical changes associated with ambroxol therapy for the treatment of patients with Parkinson disease with and without mutations in the glucocerebrosidase gene.

## Introduction

Mutations in the glucocerebrosidase gene, *GBA1* (OMIM 606463), cause the autosomal recessive lysosomal storage disorder, Gaucher disease.^1^ These mutations are the most important genetic risk factor for Parkinson disease (PD),^2^ exhibiting penetrance of 10% to 30%.^3,4^ They are present in 5% to 15% of Caucasian patients with PD, 25% of Ashkenazi Jewish patients with PD, and 1% of individuals without PD.^5^

In *GBA1* cell and animal models, an increased α-synuclein accumulation and a reciprocal relationship between α-synuclein levels and β-glucocerebrosidase (GCase) enzyme activity have been reported.^6,7,8,9,10,11^ Although GCase activity is reduced in the brains of patients with PD with and without *GBA1* mutations, it is lower in those with the mutations.^12^ Reduced GCase activity in the brain is associated with increased levels of α-synuclein.^13^ In addition, GCase activity is decreased in the cerebrospinal fluid (CSF) of patients with PD with and without *GBA1* mutations compared with controls.^14^ Upregulation of brain cytosolic/lysosomal GCase activity may reduce α-synuclein levels, mediating a neuroprotective effect in patients with PD both with and without *GBA1* mutations.^15,16,17^

Ambroxol therapy has been safely used as a cough linctus since the 1970s (summary of product characteristics in eMethods 1 in Supplement 2). Its principal adverse effects are gastrointestinal disturbance and a small risk of anaphylaxis. A high-throughput compound evaluation indicated that ambroxol delivered a pH-dependent increase in GCase activity.^18^

Ambroxol administration has also been reported to increase GCase activity and reduce α-synuclein levels in vitro and in vivo.^6,7,8,9,10,11^ Ambroxol is an inhibitory chaperone that mobilizes the sequestered mutant GCase from the endoplasmic reticulum by binding to and inhibiting the enzyme active site, inducing conformational change and facilitating transportation to the lysosome.^19,20^ In the acidic lysosome, ambroxol is eluted, allowing normal catalysis to resume and restoring lysosomal function.

Ambroxol may modulate α-synuclein levels through several mechanisms. The GCase may have a direct role in α-synuclein protein disposal,^21,22^ and ambroxol has been shown to upregulate GCase expression through the transcription factor EB pathway and stimulation of lysosomal exocytosis.^9,23^ Alternatively, *GBA1* mutations may interrupt physiologic posttranslational folding, preventing transportation of the enzyme to the lysosome.^19,20,24,25^ This interruption appears to result in sequestration in the endoplasmic reticulum and an unfolded protein response that may induce α-synuclein aggregation.^24^ Evidence also suggests that ambroxol corrects posttranslational folding, mitigating unfolded protein response.^19^

We investigated the biological changes associated with ambroxol therapy in human participants and its consequences on the biochemical and clinical markers of PD. The primary end points were safety and tolerability of ambroxol therapy, central nervous system penetration, and change in CSF GCase activity between baseline and 186 days.

## Methods

### Study Design and Participants

We performed a single-center open-label noncontrolled clinical trial of oral ambroxol therapy (escalating dose to 1.26 g per day [420 mg 3 times per day]) in patients with PD of moderate severity. The clinical trial was conducted at the Leonard Wolfson Experimental Neuroscience Centre, a dedicated clinical research facility at the University College London Queen Square Institute of Neurology from January 11, 2017, to April 25, 2018. A clinical trial steering committee provided clinical oversight. Statistical support was provided by the Peninsula Clinical Trials Unit of the University of Plymouth in the United Kingdom. The study was approved by the Institutional Joint Research Office of University College London, the UK Medicines and Healthcare products Regulatory Agency, and the London-Bloomsbury Research Ethics Committee. Written informed consent was obtained from all participants before study-related procedures were initiated. The study was conducted in accordance with the ethical principles of the Declaration of Helsinki^26^ and the *Guideline for Good Clinical Practice.*^27^ The trial protocol is available in Supplement 1, and a detailed summary of study visits is available in eTable 1 in Supplement 2.

Patients were recruited from the established research databases of the National Hospital for Neurology and Neurosurgery and the Royal Free London Hospital. Eligible participants were aged 40 to 80 years, had idiopathic PD as defined by Queen Square Brain Bank criteria,^28^ were judged able to self-administer the clinical trial drug, and were at stage 3 or less on the Hoehn and Yahr Scale, which classifies the progression of PD symptoms in 5 stages (stage 1 indicates unilateral involvement only, and stage 5 indicates wheelchair-bound or bedridden unless aided). A portion of participants were preselected on the basis of their known *GBA1* mutation–carrier status. Key exclusion criteria included the use of an interventional medicinal product within the last 30 days or exposure to 3 or more interventional medicinal products within the last 12 months. All participants underwent confirmatory sequencing of exons 1 to 11 of the *GBA1* gene. A full list of exclusion and inclusion criteria can be found in eMethods 2 in Supplement 2.

At the evaluation for clinical trial entry, each patient underwent a physical and neurological examination, an electrocardiogram, and blood sampling for clinical laboratory tests. Women of childbearing age also received a pregnancy test. After confirmation of patient eligibility, clinical visits were held at baseline, day 11, day 93, day 186, and day 279, with telephone contact at predetermined intervals (eTable 1 in Supplement 2). In addition to routine clinical blood tests, blood samples were taken at each assessment. Cerebrospinal fluid examination was performed at baseline and 186 days, with a third optional lumbar puncture (LP) at 279 days.

The 186-day exposure period comprised 28 days of dose escalation, with each dose administered 3 times per day as follows: 60 mg (days 1-7), 120 mg (days 8-14), 180 mg (days 15-21), and 300 mg (days 22-28). This exposure period was followed by 158 days of administration of ambroxol at 1.26 g per day (420 mg 3 times per day). Patients were issued ambroxol therapy in 2 batches, 1 at baseline and 1 at 93 days. Ambroxol tablets were donated by PRO.MED.CS Praha a. s. (Prague, Czech Republic).

All patients were taking dopaminergic therapy, and levodopa dosage equivalents have been included in Table 1. Patients attended each visit in an off-medication state, defined as more than 8 hours (overnight) of withdrawal of levodopa or more than 24 hours of withdrawal of levodopa for those taking modified-release dopamine agonists.

**Table 1.** Baseline Characteristics of Participants Who Completed the Study

Characteristic	Mean (SD)		
	Total Participants (n = 18)	Participants With *GBA1* Mutation (n = 8)	Participants Without *GBA1* Mutation (n = 10)
Age, y	60.2 (9.7)	56.1 (9.2)	63.4 (9.2)
Men, No. (%)	15 (83.3)	7 (87.5)	8 (80.0)
Hoehn and Yahr stage, median (range)^a^	2 (1-3)	2 (1-3)	2 (1-3)
Age at onset, y	51.7 (11.5)	44.5 (7.9)	58.9 (10.2)^b^
Levodopa equivalence, mg	743 (484)	756 (564)	733 (442)

Abbreviation: *GBA1*, glucocerebrosidase gene.

^a^ Recorded in the on state.

^b^ *P* = .007.

Assessments were performed between 8 am and 9 am by a single assessor (S.M.). Measurement instruments included (1) the Movement Disorder Society Unified Parkinson Disease Rating Scale (MDS-UPDRS; score range, 0-272, with 0 indicating no disability and 272 indicating total disability), which was administered on days 0, 93, 186, and 279; (2) the Montreal Cognitive Assessment (MoCA; score range, 0-30, with ≥26 indicating normal cognitive function), which was administered on days 0 and 186; (3) the Non-Motor Symptoms Scale (NMSS; score range, 0-360, with 0 indicating no severity and frequency of symptoms and 360 indicating high severity and frequency of symptoms), which was administered on days 0 and 186; and (4) the Non-Motor Symptoms Questionnaire (NMSQuest; 30 items evaluating the presence of nonmotor symptoms, with 0 indicating no presence of symptoms and 30 indicating maximum presence of symptoms), which was administered on days 0 and 186.

Empty ambroxol blister packs were collected at 93 and 186 days to assess participant adherence to treatment. All adverse events (AEs), biochemical results (eTable 2 in Supplement 2), and measurements of blood pressure, heart rate, and weight were recorded.

### Outcomes

The primary outcomes, all assessed at 186 days, were change in CSF ambroxol levels and change in CSF GCase activity. The predefined secondary outcomes, all assessed at 186 days, were the safety and tolerability of ambroxol in the study population (measured by the frequency and severity of AEs and abnormal findings on clinical examinations, blood tests, or electrocardiograms), the change in blood leucocyte GCase activity, the change in CSF GCase protein levels, the CSF total glucosylceramide levels, the CSF and serum α-synuclein levels, and the CSF and serum tau levels. We also recorded the results of the MoCA, NMSS, and NMSQuest assessments that were conducted at baseline and 186 days.

### Sample Collection, Assays, and Statistics

Cerebrospinal fluid was collected before 10 am from participants after they had fasted overnight. Samples were frozen at *–*80 °C within 60 minutes of collection in the case of CSF and within 90 minutes of collection in the case of leucocyte pellets. Samples were only defrosted immediately before the performance of assays. Cerebrospinal fluid GCase assays were all performed within 7 and 14 days of sample collection. Leucocyte GCase assays were performed between 7 and 28 days of sample collection.

Details of the biochemical assays are provided in eMethods 3 and 4 in Supplement 2.

The statistical analyses followed a predefined statistical analysis plan written by the clinical trial statisticians (A.H.V.S. and J. Hosking). As the result of unforeseen circumstances, the statistician specified in the original protocol was not available to perform the final analysis. Therefore, a revised statistical analysis plan (eMethods 5 in Supplement 2) was produced before data analysis was conducted and the study was completed. All data analyses were performed using Stata software, version 14.2 (StataCorp LLC). The distribution of the outcomes was assessed through inspection of the plotted data (eFigures 1, 2, and 3 in Supplement 2). For the primary analysis, 95% CIs were presented alongside the results from a 2-sided *t* test (significance threshold of *P* < .05) of the change in CSF GCase activity. The lower CI and 1-sided *t* test results (significance threshold of *P* < .05) that were presented for the change in CSF ambroxol levels reflected the fact that the change was lower bounded by 0 because of anticipated 0 concentrations at baseline. To register as a change, ambroxol concentrations were required to be greater than the assay limit of detection of 0.5 ng/mL at 186 days. A descriptive analysis that correlated the change (Pearson coefficient) in ambroxol levels and the change in CSF GCase activity was also performed. The changes in secondary outcomes between baseline and 186 days were presented with 95% CIs. All data analyses were performed from November 1 to December 14, 2018.

## Results

**Figure 1.**
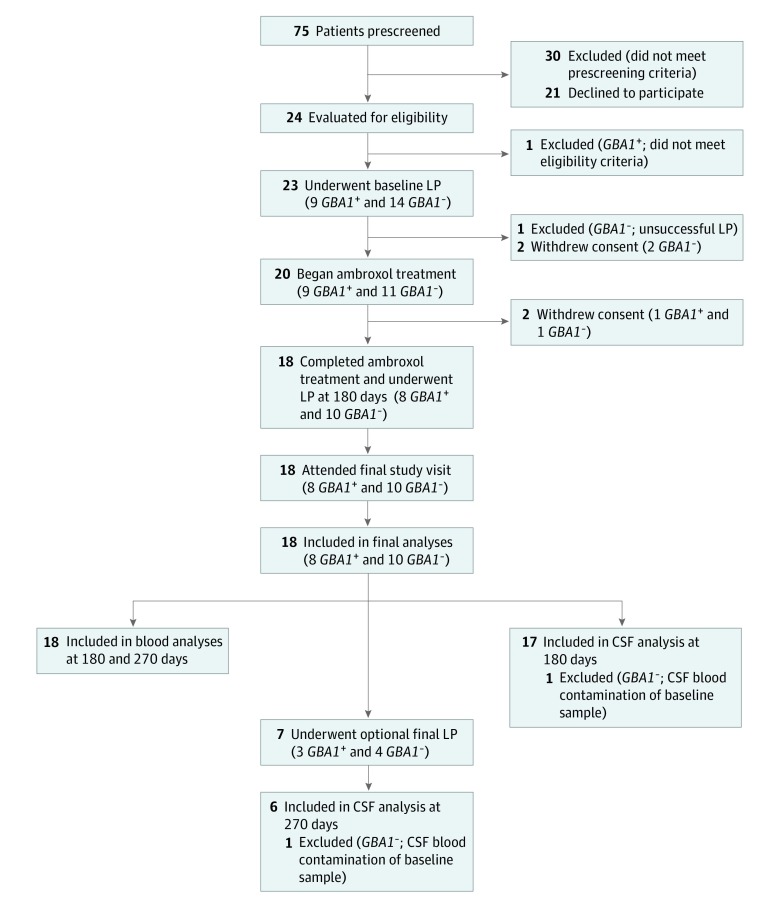
Flow Diagram of Participant Recruitment and Retention CSF indicates cerebrospinal fluid; *GBA1^-^*, negative glucocerebrosidase gene; *GBA1*^+^, positive glucocerebrosidase gene; and LP, lumbar puncture.

A total of 24 patients with moderate PD were evaluated for study eligibility. Of those, 23 patients entered the Ambroxol in the Modification of Parkinson Disease study. A total of 18 patients (15 men [83.3%]; mean [SD] age, 60.2 [9.7] years) completed the study. One patient was excluded after unsuccessful LP attempts that were performed by 2 experienced operators. Two patients withdrew because they experienced headaches after LP and before the beginning of ambroxol treatment. One participant withdrew at day 1 after treatment began, citing the high ambroxol tablet count (21 tablets per day), and 1 withdrew at 93 days after treatment began, citing family illness. Of the 18 patients who completed the study, 1 was excluded from the CSF analyses (including the primary analysis) because of a red blood cell contamination of more than 500 cells per cm^3^ in the baseline CSF sample (flow diagram of participant recruitment and retention in Figure 1).

Participants with *GBA1* mutations had the following variants (an asterisk indicates a phenotype that is known to cause severe Gaucher disease): p.E326K wild type (3 patients), p.N370S wild type (1 patient), p.R463C* wild type (2 patients), p.T369M/p.W393X* (1 patient), and RecNcil (p.L444P, p.A456P, and p.V460V)* wild type (1 patient).

Seven participants (3 with *GBA1* mutations [*GBA1*^+^] and 4 without mutations [*GBA1^–^*]) underwent a third LP at 279 days. These 7 participants included the patient who was excluded from the analyses because of a blood-contaminated baseline CSF sample. As a result, 18 participants (8 *GBA1*^+^ and 10 *GBA1^–^*) were included in blood and clinical analyses at 186 and 279 days. Seventeen participants (8 *GBA1*^+^ and 9 *GBA1^–^*) were included in the CSF analyses at 186 days, and 6 participants (3 *GBA1*^+^ and 3 *GBA1^–^*) were included in the CSF analyses at 279 days.

Based on the number of empty ambroxol blister packs collected, we estimated a mean (SD) adherence to treatment of 89% (13%). Blood and CSF ambroxol levels at baseline confirmed that no participants had taken the drug before the treatment start date.

Table 2 shows a summary of results for participants who completed the study. The results of the *GBA1*^+^ and *GBA1^–^* subgroups are available in eTables 3 and 4 in Supplement 2.

**Table 2.** Results of Participants Who Completed the Study

Result^a^	Mean (SD)			
	Baseline	Day 11	Day 93	Day 186
Total participants, No.				
Blood	18	18	18	18
CSF	17	17	17	17
Ambroxol, ng/mL				
Blood serum	0	316 (196)	1084 (396)	1432 (570)^b^
CSF	0	NA	NA	156 (53)^b^
GCase activity				
Blood leucocytes, nmol/mg/h	11.0 (5.2)	12.8 (4.9)	13.1 (4.8)	12.0 (5.2)
CSF, nmol/mL/h	0.309 (0.153)	NA	NA	0.250 (0.142)^b^
GCase protein level, pmol/L				
CSF	250 (47)	NA	NA	338 (104)^b^
α-Synuclein, pg/mL				
Blood serum	20 793 (9418)	19 991 (7380)	24 964 (9391)	23 395 (9998)
CSF	383 (103)	NA	NA	433 (117)^b^
Tau, pg/mL				
Blood serum	1.00 (0.25)	0.84 (0.24)	0.88 (0.22)	0.80 (0.24)^b^
CSF	206 (59)	NA	NA	211 (63)
Glucosylceramide, pmol/L				
CSF	246 (83)	NA	NA	260 (80)
MDS-UPDRS score				
Part 3	31.1 (14.5)	NA	27.2 (10.7)	24.3 (12.1)^b^
Total	62.6 (32.2)	NA	57.7 (27.6)	53.9 (30.3)^b^
MoCA score	25.0 (4.8)	NA	NA	26.7 (4.0)
NMSS score	49.3 (36.1)	NA	NA	60.8 (38.6)^b^
NMSQuest score	10.6 (6.0)	NA	NA	10.8 (6.0)
Weight, kg	83 (17)	83 (17)	82 (17)	82 (17)^b^
Arterial blood pressure, mm Hg	90 (8)	88 (9)	90 (10)	90 (11)

Abbreviations: CSF, cerebrospinal fluid; GCase, glucocerebrosidase enzyme; MDS-UPDRS, Movement Disorders Society Unified Parkinson’s Disease Rating Scale; MoCA, Montreal Cognitive Assessment; NA, not applicable; NMSQuest, Non-Motor Symptoms Questionnaire; NMSS, Non-Motor Symptoms Scale.

^a^ Clinical markers are recorded in the off state.

^b^ Indicates a significant change from baseline to 186 days (analyzed by *t* test).

### Primary and Secondary Outcomes

**Figure 2.**
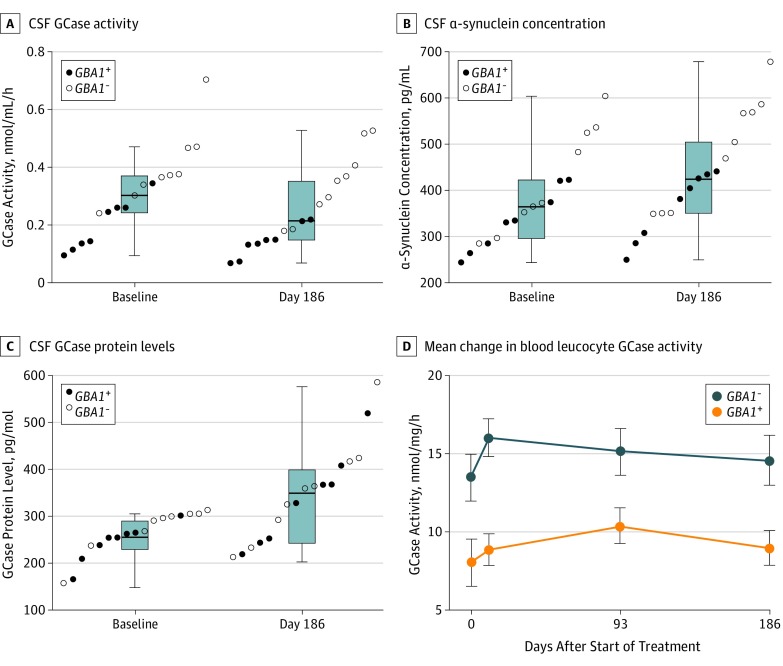
Biochemical Changes After Ambroxol Administration A, Box plot with superimposed data points at baseline (median, 0.321; interquartile range [IQR], 0.142) and 186 days (median, 0.216; IQR, 0.221). Analysis included 17 participants (8 *GBA1^+^* and 9 *GBA1^–^*). Mean (SE) change of 0.059 (0.026) nmol/mL per hour (95% CI, –0.115 to –0.002; 2-sided paired *t* test, *P* = .04) represents a 19% decrease. B, Box plot (median and IQR) with superimposed data points at baseline and 186 days. Analysis included 17 participants (8 *GBA1^+^* and 9 *GBA1^–^*). Mean (SE) change of 50 (17) pg/mL (95% CI, 14-87; 2-sided paired *t* test, *P* = .01) represents a 13% increase. C, Box plot (median and IQR) with superimposed data points at baseline and 186 days. Analysis included 17 participants (8 *GBA1^+^* and 9 *GBA1^–^*). Mean (SE) change of 88 (22) pmol/L (95% CI, 40-137; 2-sided paired *t* test, *P* = .002) represents a 35% increase. All data points are horizontally offset for ease of interpretation. D, Error bars indicate SE of the mean. Analysis included 18 participants (8 *GBA1^+^* and 10 *GBA1^–^*). Mean (SE) change between baseline and 186 days of 1.0 (1.4) nmol/mg per hour (95% CI, –2.0 to 4.0; *P* = .48) represents a 9% increase. CSF indicates cerebrospinal fluid; *GBA1^-^*, negative glucocerebrosidase gene; *GBA1*^+^, positive glucocerebrosidase gene; and GCase, glucocerebrosidase enzyme.

Ambroxol was undetectable in blood serum and CSF at baseline. At day 186, the CSF ambroxol level was 156 ng/mL (95% lower confidence limit, 129 ng/mL; 1-sided paired *t* test, *P* < .001). At 186 days, the mean (SE) CSF ambroxol levels were 11% (6%) of the mean blood levels. As anticipated by our pretrial CSF studies, which indicated inhibition of GCase in CSF rather than enhancement in tissues, the mean (SE) CSF GCase activity decreased by 0.059 (0.026) nmol/mL per hour (95% CI, -0.115 to -0.002; 2-sided paired *t* test, *P* = .04; Figure 2A), which was a 19% reduction from the mean baseline GCase activity.

The drug was well tolerated, and no serious AEs were reported. A total of 176 AEs occurred, of which 121 were deemed unrelated, 32 unlikely to be related, 15 possibly related, 5 probably related, and 3 definitely related to treatment. The AEs deemed to be probably related to the interventional medicinal product were nausea (1 patient), vomiting (2 patients), a burning sensation after swallowing the interventional medicinal product (1 patient), and loose stool (1 patient). Definitely related AEs were acid reflux (1 patient), nausea (1 patient), and a transitory skin condition on the chest, back, and arms (1 patient). A full list of AEs is available in eTable 5 and a list of recorded AEs is available in eTable 6 in Supplement 2. A mean (SD) weight loss of 1.3 (2.5) kg was observed between baseline and 186 days (95% CI, –2.59 to 0.01). No protocol deviations occurred.

Between baseline and 186 days, mean (SE) increases of 50 (17) pg/mL (13%) in total CSF α-synuclein concentration (95% CI, 14-87; *P* = .01; Figure 2B) and 88 (22) pmol/L (35%) in CSF GCase protein levels (95% CI, 40-137; *P* = .002; Figure 2C) were observed. Effect sizes did not indicate a significant change in CSF tau (mean [SE] change, 5 [8] pg/mL; 95% CI, –7 to 17; *P* = .36) or glucosylceramide levels (mean [SE] change, 14 [9] pmol/L; 95% CI, –6 to 35; *P* = .16). The correlation between ambroxol concentration and the change in CSF GCase activity was not significant (Pearson coefficient, *r* = –0.161; *P* = .52). The CSF analysis of the 6 participants from whom CSF was collected at baseline, 186 days, and 279 days is presented in eTable 4 in Supplement 2.

Between baseline and 186 days, mean (SE) blood leucocyte GCase activity increased by 1.0 (1.4) nmol/mg/h (95% CI, –2.0 to 4.0; *P* = .48) (Table 2 and Figure 2D). Wide variation was noted in the recorded change in serum α-synuclein concentration (mean [SE] change, 2602 [3649] pg/mL; 95% CI, –4689 to 9893; *P* = .46), although a mean (SE) decrease of 0.20 (0.08) pg/mL was observed in serum tau levels (95% CI, –0.37 to -0.05; *P* = .01).

Between baseline and 186 days, the mean (SD) total MDS-UPDRS score decreased (ie, improved) by 8.7 (11.8) points (95% CI, –15.3 to –2.2; *P* = .01) with a mean (SD) rebound of 7.2 (9.8) points between 186 and 279 days. This change appeared to be associated primarily with the MDS-UPDRS part 3 motor score (score range, 0-56, with 0 indicating no motor impairment and 56 indicating severe motor impairment), which showed a mean (SD) decrease of 6.8 (7.1) points between baseline and 186 days (95% CI, –10.4 to –3.1; *P* = .001) and a mean (SD) increase of 7.6 (7.0) points between 186 and 279 days. Seven of 18 patients increased their dopaminergic therapy during the course of the study, and the same deflection of MDS-UPDRS changes was observed in those on stable therapy and in those who increased their medication therapy (eFigures 4 and 5 in Supplement 2).

As anticipated, the change in MoCA scores were skewed and bound by 0 because of the preponderance of participants who maintained their scores from baseline. An increase (ie, improvement) in the mean (SD) MoCA scores of 1.7 (1.3) points was recorded between baseline and 186 days. In addition, the mean (SD) NMSS score increased (ie, worsened) by 11.5 (18.5) points (95% CI, 2.4-20.8; *P* = .02), but the mean (SD) change in the NMSQuest score was only 0.2 (2.6) points (95% CI, –1.5 to 1.0; *P* = .72) between baseline and 186 days.

### In Vitro Assays

Before the clinical trial, we performed in vitro assays to estimate the association of ambroxol, an inhibitory chaperone, with changes in GCase activity in acellular CSF. We added 500nM (189 ng/mL, chosen on the basis of CSF ambroxol levels reported in a previous clinical trial)^17^ of ambroxol to human CSF taken from healthy participants (via diagnostic LPs for suspected idiopathic intracranial hypertension).

In addition, we performed a positive control by thermodynamically denaturing and chemically inhibiting GCase with conduritol B epoxide.^29^ The experiment comprised 5 technical repeats for CSF derived from 5 participants for each condition. Compared with the control samples, a mean (SD) decrease of 42% (12%) in CSF GCase activity (mean [SD] change in activity, –0.093 [0.026]; 95% CI, 0.046-0.140 nmol/mL per hour; *P* = .01) was observed after the addition of ambroxol. The denatured and conduritol B epoxide–inhibited samples registered no residual activity.

## Discussion

To our knowledge, this study represents the first clinical trial of personalized therapy for a stratified (genetically defined) subtype of PD and the first use of ambroxol therapy in patients with PD. The study met its primary outcomes, which were to confirm that ambroxol was able to penetrate CSF and that it had a modulatory effect on CSF GCase. The finding that ambroxol penetrates CSF is consistent with a recent clinical trial of ambroxol (at an equivalent dose) in 5 patients with a mean age of 18 years who had neuronopathic Gaucher disease.^17^ Our study indicates that ambroxol therapy is well tolerated in patients with PD. This finding is important, as the administered dose was approximately 10 times higher and was administered for a longer duration than specified in its license.

Ambroxol has an inhibitory effect on GCase activity within acellular human CSF, which is consistent with its known activity as an inhibitory chaperone with a neutral pH. Within largely acellular CSF, the GCase protein is free, which is in contrast to its normal intracellular lysosomal location. Ambroxol is a pH-dependent inhibitory small molecular chaperone that binds to the active site of the GCase protein and reduces activity. Binding enables transportation to the lysosome and elution of free active enzymes under acidic conditions. Therefore, in acellular CSF, ambroxol will bind to and inhibit free GCase. However, in tissues, including those in the brain, ambroxol will increase intracellular GCase activity, as reported in studies of rodent and primate models^7,8^

The sustained upregulation of expression of CSF GCase protein levels indicates target engagement of ambroxol with the GCase pathway. The increase in total CSF α-synuclein concentration implies, based on previous in vitro and in vivo data,^6,7,8,9,11,15^ that ambroxol has also had an association with α-synuclein metabolism. These results suggest an increase in GCase activity within the brain itself, although this activity cannot, of course, be measured in vivo. No clear consensus exists on the association of PD with changes in total CSF α-synuclein, but reduced levels of total CSF α-synuclein have been described, while oligomeric and phosphorylated α-synuclein have increased.^30^ Ambroxol upregulates the expression of GCase, probably via the transcription factor EB pathway, and increases vesicular export.^9,23^ The increase in CSF α-synuclein could be interpreted as an increase of extracellular export of the protein from the brain parenchyma.

Interpretation of the changes in MDS-UPDRS and MoCA results is difficult in the context of a nonplacebo-controlled study. However, the changes support the clinical impression that no substantial deleterious effect of ambroxol was observed among participants taking ambroxol, including any adverse effect on the motor features of their PD.

### Limitations

Our study has several limitations. The sample was relatively small, although the complexity of the study and its nature as a proof-of-concept clinical trial were factors in its design. A placebo arm was not used, so the clinical outcomes should be interpreted with caution. We could have elected to have a more genetically homogenous *GBA1* study group, but this homogeneity may have limited the interpretation of the data. The mean age of our participants was 60.2 years, which is relatively young for patients with PD. However, this mean age was consistent with our inclusion criteria of a Hoehn and Yahr stage of 3 or less and was comparable with many drug intervention studies of patients with PD. Participants were recruited from databases of patients with PD with and without *GBA1* mutations, but we do not believe this recruitment method resulted in a bias relevant to the biochemical biomarkers used as end points in this study.

## Conclusions

In conclusion, we confirm that ambroxol has potential as a drug to target the glucocerebrosidase pathway in PD and increase GCase activity in the brain. These findings concur with cell and animal modeling, which indicate that ambroxol modulates α-synuclein levels. We believe ambroxol therapy has promise for further investigation as a drug to improve outcomes, particularly in patients who have PD with a *GBA1* mutation and potentially in those without a *GBA1* mutation. Larger placebo-controlled studies are warranted.

## Notes

Supplement 1. **Trial Protocol**

Supplement 2. **eMethods 1.** Summary of Product Characteristics of Ambroxol **eMethods 2.** Inclusion and Exclusion Criteria **eMethods 3.** Details of Biochemical Assays **eMethods 4.** Optimization Assays for Liquid Chromatography Mass Spectrometry of Ambroxol **eMethods 5.** Statistical Analysis Plan **eFigure 1.** Change in CSF Ambroxol Concentration Plotted Against Inverse of Normal **eFigure 2.** Change in CSF GCase Activity Plotted Against Inverse of Normal **eFigure 3.** Scatter Plot of Change in CSF GCase Activity Against Change in CSF Ambroxol **eFigure 4.** Influence of Change in PD Meds on MDS-UPDRS Total Score **eFigure 5.** Influence of Change in PD Meds on MDS-UPDRS Part 3 Score **eTable 1.** Summary of Study Visits **eTable 2.** Summary Table of Biochemical Results Including *GBA1* and Non-*GBA1* Subgroups **eTable 3.** Summary Table of Clinical Results Including *GBA1* and Non-*GBA1* Subgroups **eTable 4.** Summary Table of CSF Biochemical Results Including *GBA1* and Non-*GBA1* Subgroups in Those Who Underwent 3 Lumbar Punctures **eTable 5.** Summary Table of Adverse Events Felt to Be Possibly, Probably, or Definitely Related to Ambroxol **eTable 6.** List of Recorded Adverse Events
