# Supplementary material for: Ambroxol for the Treatment of Patients With Parkinson Disease With and Without Glucocerebrosidase Gene Mutations: A Nonrandomized, Noncontrolled Trial
Source: JAMA Neurol. 2020 Jan 13;77(4):427–34. doi: 10.1001/jamaneurol.2019.4611 (PMC6990847; doi:10.1001/jamaneurol.2019.4611)
Supplement: Supplement 1. — Trial Protocol [file jamaneurol-77-427-s001.pdf]

AiM-PD Study

A Phase IIA Prospective, Single-Centre, Open Label Clinical Trial to Evaluate the Safety, Tolerability and Pharmacodynamic Effects of Ambroxol in Patients with Parkinson Disease:

Ambroxol in Disease Modification in Parkinson Disease

Version 2.2 Date: 01.03.2017

Chief Investigator: Professor Anthony Schapira

Study Site: Leonard Wolfson Experimental Neurology Centre (LWENC)

Sponsor: University College London (UCL)

Funder: Cure Parkinson's Trust

Investigational Medicinal Product: Ambroxol hydrochloride
(Brand Name: Ambrosan)

EudraCT Number: 2015-002571-24
Sponsor Code: 15/0118

Key Trial Contacts

**Chief and Principal Investigator**	Professor Anthony Schapira MD DSc FRCP FMedSci<br>Head of Department of Clinical Neurosciences<br>University College London (UCL)<br>Professor of Neurology and Consultant at National Hospital of Neurology and Neurosurgery and the Royal Free Hospital<br>3B-94, Upper 3rd Floor<br>Institute of Neurology<br>Hampstead Campus<br>Rowland Hill St<br>London<br>NW3 2PF<br>Telephone Number: +44 (0) 207 679 6802<br>Fax Number: +44 (0) 203 108 2312<br>Email: a.schapira@ucl.ac.uk
**Trial Coordinator**	Dr Philip Woodgate BSc MRes PhD<br>Clinical Trials Coordinator<br>Leonard Wolfson Experimental Neurology Centre<br>University College London (UCL)<br>Institute of Neurology & The National Hospital for Neurology and Neurosurgery<br>Queen Square<br>London<br>WC1N 3BG<br>Telephone Number: +44 (0) 203 448 4531<br>Fax Number: +44 (0) 203 448 4547<br>Email: p.woodgate@ucl.ac.uk
**Sponsor Representative**	Adedayo Akinyemi<br>Sponsor Regulatory Advisor<br>University College London (UCL)<br>Joint Research Office<br>Gower Street,<br>London WC1E 6BT<br>Telephone Number: +44 (0) 207 679 6469<br>Fax Number: +44 (0) 203 108 2312<br>Email: a.akinyemi@ucl.ac.uk

**Co-Investigators**	Dr Vincenzo Libri MD PhD FRCP<br>Director of NIHR/Wellcome UCLH Clinical Research Facility<br>Head of Leonard Wolfson Experimental Neurology Centre<br>Director of the MRes in Translational Neurology<br>University College London (UCL)<br>Institute of Neurology & The National Hospital for Neurology and Neurosurgery<br>23 Queen Square<br>London<br>WC1N 3BG<br>Telephone Number: +44 (0) 203 448 4538 /4101<br>Fax Number: +44 (0) 203 448 4547<br>Email Address: vincenzo.libri@ucl.ac.uk<br><br>Dr Stephen Mullin MRCP<br>University College London (UCL)<br>Institute of Neurology<br>Royal Free Campus<br>Rowland Hill Street<br>Hampstead<br>London<br>NW3 2PF<br>Telephone Number: + 44 (0) 207 794 0500 Ext: 34374<br>Fax Number: +44 (0) 203 108 2312<br>Email address: stephen.mullin@ucl.ac.uk<br><br>Dr Thomas Foltynie BSc MBBS MRCP PhD<br>Senior Lecturer and Honorary Consultant Neurologist<br>Unit of Functional Neurosurgery<br>University College London (UCL)<br>Institute of Neurology & The National Hospital for Neurology and Neurosurgery<br>Queen Square<br>London<br>WC1N 3BG<br>Telephone Number: +44 (0) 203 456 7890<br>Email Address: t.foltynie@ucl.ac.uk<br><br>Professor Henrik Zetterberg MD PhD<br>Department of Neurochemistry<br>Department of Molecular Neuroscience<br>University College London (UCL)<br>Institute of Neurology<br>Queen Square<br>London<br>WC1N 3BG<br>Telephone Number: +46 768 672 647<br>Fax Number: +46 31 3432426<br>Email Address: h.zetterberg@ucl.ac.uk

**Statistician**	Dr Les Huson PhD<br>Honorary Lecturer in Medical Statistics<br>University College London (UCL)<br>Institute of Neurology<br>23 Queen Square<br>London<br>WC1N 3BG<br>Email Address: l.huson@ucl.ac.uk
**IMP Supplier**	PRO.MED.CS Praha a.s<br>Telčská 1<br>140 00 Prague 4<br>Czech Republic<br>Telephone Number: +420 2410 13111<br>Fax Number: +420 2410 13412
**Central Laboratories**	University College Hospitals NHS Foundation Trust (UCLH) Clinical Biochemistry Laboratory<br>University College Hospital NHS Trust<br>60 Whitfield Street<br>London<br>W1T 4EU<br>Telephone Number: +44 (0) 203 447 9405<br>Fax Number: +44 (0) 203 447 9584<br><br>Neuroimmunology and CSF Laboratory (NICL)<br>National Hospital for Neurology and Neurosurgery (NHNN)<br>9th Floor Queen Square House<br>Queen Square<br>London<br>WC1N 3BG<br>Telephone Number: +44 (0) 203 448 3814<br>Fax Number: +44 (0) 203 448 3797
**Local Laboratories**	Neurogenetics Unit<br>6th Floor<br>Institute of Neurology<br>Queen Square<br>London<br>WC1N 3BG<br>Telephone Number: +44 (0) 203 448 4250<br><br>Institute of Child Health & Great Ormond Street Hospital for Sick Children<br>Centre for Translational Omics<br>Centre for Inborn Errors of Metabolism<br>University College London<br>30 Guilford Street<br>London<br>WC1N 1EH<br>Telephone Number: +44 (0) 207 905 2873

	Haematology Molecular Diagnostics / Lysosomal Storage Disorders Unit Laboratory<br>Health Service Laboratories<br>Royal Free London NHS Foundation Trust<br>Pond Street<br>London<br>NW3 2QG<br>Telephone Number: +44 (0) 207 794 0500 ext 33273<br><br>Laboratory of the Government Chemist (LGC)<br>Queens Road<br>Middlesex<br>TW11 0LY<br>Telephone Number: +44 (0) 208 943 7675<br>Fax Number: +44 (0) 208 943 2767<br><br>Clinical Neurochemistry Laboratory<br>Institute of Neuroscience and Physiology<br>University of Gothenburg<br>House V3<br>Sahlgrenska University Hospital / Mölndal<br>SE-431 80 Mölndal, Sweden<br>Telephone Number: +46 31 343 00 25 or +46 31 343 24 06<br>Fax Number: +46 31 41 92 89

Protocol Synopsis

**Full Title of Study**	A Phase IIA Prospective, Single-Centre, Open Label Clinical Trial to Evaluate the Safety, Tolerability and Pharmacodynamic Effects of Ambroxol in Patients with Parkinson Disease: **A**mbroxol **i**n Disease **M**odification in **P**arkinson **D**isease
**Short Title of Study**	**AiM-PD**
**Clinical Phase**	Phase IIA
**Disease Type**	Parkinson disease
**Study Design**	A single-centre, interventional, open label clinical trial to assess the safety, tolerability and pharmacodynamics effects of ambroxol – a proof of concept clinical trial of an investigational medicinal product (IMP).
**Primary Objective**	• To assess the central nervous system (CNS), cerebrospinal fluid (CSF) penetration and binding to GCase of ambroxol by the parameters outline (modulation of GCase activity & ambroxol level) at 5 intra-participant dose escalations from day 1 to day 186 at 60 mg TID (day 1-7), 120 mg TID (day 8-14), 180 mg TID (day 15-21), 300 mg TID (day 22-28) and 420 mg TID (day 29-186).
**Secondary Objectives**	• To assess the safety and tolerability of the Glucocerebrosidase (GCase) modulating chaperone ambroxol in Parkinson disease participants with and without Gaucher gene (*GBA*) mutation at 5 intra-participant dose escalations from day 1 to 186.<br>• To measure the pharmacodynamic effects of ambroxol on GCase activity in blood and CSF following ambroxol oral administration at 5 intra-participant dose escalations from day 1 to 186.<br>• To quantify the effect of ambroxol on biomarkers of Parkinson and neurodegeneration at 5 intra-participant dose escalations from day 1 to 186.
**Exploratory Objective**	• To explore whether ambroxol leads to improvement in cognitive performance, Montreal Cognitive Assessment (MoCA) and the motor and non-motor features of Parkinson disease from day 1 to day 186.<br>• To explore whether ambroxol leads to improvement in Parkinson Disease non-motor symptom assessment scale (NMSS) and non-motor symptom questionnaire (NMSQuest) from day 1 to day 186.
**Inclusion Criteria**	1. Male or female;<br>2. Age ≥ 40 and ≤ 80 years of age;<br>3. Confirmed diagnosis of Parkinson disease at any time; and Hoehn and Yahr criteria, confirmed staged between I – III, inclusive;<br>4. Able and willing to provide informed consent prior to any study related assessments and procedures at screening visit 1;<br>5. Capable of complying with all study procedures, including fasting lumbar puncture;<br>6. Willing to provide a blood sample for screening genomic for Parkinson Disease related DNA analysis and/or consent to

	Investigators obtaining and using participants previous *DNA* results if applicable;<br>7. Willing and able to self-administer oral ambroxol medication, from day 1 to 186 (at 60 mg TID (day 1-7), 120 mg TID (day 8-14), 180 mg TID (day 15-21), 300 mg TID (day 22-28) and 420 mg TID (day 29-186));<br>8. Able to travel to the participating study site;<br>9. A female participant is eligible to participate if she is of:<br>• Non-childbearing potential defined as pre-menopausal females with a documented tubal ligation or hysterectomy; or postmenopausal defined as 12 consecutive months of spontaneous amenorrhea, at least 6 weeks post-surgical bilateral oophorectomy (with or without hysterectomy) or post tubal ligation. In questionable cases, menopausal status will be confirmed by demonstrating levels of follicle stimulating hormone (FSH) 25.8 – 134.8 IU/L and oestradiol < 201 pmol/l at entry.<br>• Women of child-bearing potential must use accepted contraceptive methods (listed below), and must have a negative serum at screening visit 1 and urine pregnancy tests at subsequent visits if applicable. An additional pregnancy test will be performed, and results obtained, prior to administration of the first dose of ambroxol.<br><br>Accepted contraception methods:<br>• True abstinence: When this is in line with the preferred and usual lifestyle of the participant. [Periodic abstinence (e.g., calendar, ovulation, symptothermal, post-ovulation methods) and withdrawal are not acceptable methods of contraception].<br><br>Contraceptive Methods with a Failure Rate of < 1%:<br>• Oral contraceptive, either combined or progestogen alone;<br>• Injectable progestogen;<br>• Implants of levonorgestrel;<br>• Estrogenic vaginal ring;<br>• Percutaneous contraceptive patches; -<br>• Intrauterine device (IUD) or intrauterine system (IUS) that meets the <1% failure rate as stated in the product label;<br>Please note:<br>▪ All male and female participants of child bearing potential must agree with their partners to use double-barrier birth control or abstinence while participating in the study and for 2 weeks following the last dose of the study drug.<br>▪ Participants may continue to take PD medications including glutamate antagonists, anticholinergics, dopamine agonists, Levodopa (L-DOPA and decarboxylase (DDC) inhibitor), Monoamine oxidase B (MAO-B) inhibitors catechol-O-methyltransferase (COMT) inhibitors, beta blockers, selective serotonin uptake inhibitors (SSRIS), tricyclic antidepressants (TCAs) and indomethacin.

**Exclusion Criteria**	Participants are excluded from participating in this study if 1 or more of the following criteria are met:<br>1. Current treatment with anticoagulants (e.g. warfarin) that might preclude safe completion of the lumbar puncture and in the opinion of the Investigator;<br>2. Current use of investigational medicinal product or participation in another interventional clinical trial or who have done so within 30 days prior to the first dose in the current study;<br>3. Exposure to more than three investigational medicinal products within 12 months prior to the first dose in the current study;<br>4. Confirmed dysphagia that would preclude self-administration of ambroxol up to 7 tablets TID for the duration of day 1 to day 186);<br>5. Significant known lower spinal malformations or other spinal abnormalities that would preclude lumbar puncture;<br>6. History of known sensitivity to the study medication, ambroxol or its excipients (lactose monohydrate, granulated microcrystalline cellulose, copovidone and magnesium stearate) in the opinion of the investigator that contraindicates their participation;<br>7. History of known rare hereditary disorders of galactose<br>8. intolerance, Lapp lactase deficiency or glucose-galactose malabsorption; Evidence or history of hypersensitivity to lidocaine or its derivatives;<br>9. History of drug abuse or alcoholism in the opinion of the Investigator that would preclude participation in the study;<br>10. Donation of blood (one unit or 350 ml) within three months prior to receiving the first dose of the study drug;<br>11. Pregnant or breastfeeding;<br>12. All participants of child bearing potential in the opinion of the Investigator that would preclude participation in the study and who do not agree to use double-barrier birth control or abstinence while participating in the study and for 2 weeks following the last dose of the study drug;<br>13. Any clinically significant or unstable medical or surgical condition that in the opinion of the PI or PI-delegated clinician may put the participant at risk when participating in the study or may influence the results of the study or affect the participant's ability to take part in the study, as determined by medical history, physical examinations, electrocardiogram (ECG), or laboratory tests.<br>Such conditions may include:<br>a) Impaired renal function<br>b) Moderate/Severe hepatic impairment<br>c) A major cardiovascular event (e.g. myocardial infarction, acute coronary syndrome, decompensated congestive heart failure, pulmonary embolism, coronary revascularisation that occurred within 6 months prior to the screening visit.
**Investigational Medicinal Product**	Ambroxol (Brand name: Ambrosan), 60 mg per tablet
**Participant Group**	10 *GBA* positive PD participants & 10 *GBA* negative PD participants
**Formulation, 5 Intra-participant Dose Escalation, Route**	Ambroxol (brand name: ambrosan), 60 mg per tablet.<br>5 intra-participant dose escalations:

**of Administration**	Dose escalation 1: Day 1-7, 60 mg TID<br>Dose escalation 2: Day 8-14, 120 mg TID<br>Dose escalation 3: Day 15-21, 180 mg TID<br>Dose escalation 4: Day 22-28, 300 mg TID<br>Dose escalation 5: Day 29-186, 420 mg TID
**Total Number of Participants**	20
**Study Site(s)**	1 (UK)
**Recruitment Period**	12 months
**Length of Study**	2 years
**Sponsor**	University College London (UCL)
**EudraCT Number**	2015-002751-24

Protocol Version History

Version Number	Date	Protocol Update Finalised By (insert name of person):	Reasons for Update

Signatures

The Chief Investigator and the Joint Research Office have discussed this protocol. The Investigator agrees to perform the investigations and to abide by this protocol.

I agree to ensure that the confidential information contained in this document will not be used for any other purpose other than the evaluation or conduct of the clinical investigation without the prior written consent of the Sponsor.

I also confirm that I will make the findings of the study publically available through publication or other dissemination tools without any unnecessary delay and that an honest accurate and transparent account of the study will be given; and that any discrepancies from the study as planned in this protocol will be explained.

I agree to conduct the trial in compliance with the approved protocol, EU GCP and UK Regulations for CTIMPs (SI 2004/1031; as amended), the UK Data Protection Act (1998), the Trust Information Governance Policy (or other local equivalent), the Research Governance Framework (2005' 2nd Edition; as amended), the Sponsor's SOPs, and other regulatory requirements as amended.

The undersigned confirm that the following protocol has been agreed and accepted and that the Chief Investigator agrees to conduct the trial in compliance with the approved protocol and will adhere to the principles outlined in the Medicines for Human Use (Clinical Trials) Regulations 2004 (SI 2004/1031), amended regulations (SI 2006/1928) and any subsequent amendments of the clinical trial regulations, Good Clinical Practice (GCP) guidelines, the Sponsor's SOPs, and other regulatory requirements as amended.

For and on behalf of the Study Sponsor:

Signature: ....

Date: 09/06/2016

Name (please print):

NICK MCNALLY

Position:

....

Chief Investigator:

Signature: ....

Date: 08/06/2016

Name: (please print):

Professor Anthony Schapira

Contents

Key Trial Contacts **1**

Protocol Synopsis **5**

Protocol Version History **9**

Signatures **10**

List of Abbreviations **15**

1. Summary **17**

2. Background and Rationale **17**

2.1 Ambroxol Justification of Route of Administration and Dosage 19

2.2 Scientific Rationale for Ambroxol 1260 mg TID maximum dose 20

3. Assessment and management of risk **21**

4. Objectives **22**

4.1 Primary Objective 22

4.2 Secondary Objectives 23

4.3 Exploratory Objective 23

5. Trial Design **23**

5.1 Overall Design 23

6. Study Settings **23**

6.1 Site Selection 23

6.2 Study Setting 23

6.3 Study Duration 24

6.4 Chief/Principal Investigator's (CI/PI) Qualification and Agreement 24

7. Eligibility Criteria **24**

7.1 Study Population 24

7.2 Participant Selection 24

7.3 Participant Inclusion Criteria 25

7.4 Participant Exclusion Criteria 26

7.5 Pregnancy and Contraception 27

8. Trial Procedures **27**

8.1 Recruitment 27

8.2 Participant Identification Centres (PIC) 27

8.3 Informed Consent Process 27

8.4 Re-screening of Participant 28

8.5 Informed consent for Genotyping/disclosure of previously held genotyping 28

8.6 Study Procedure Assessments 28

8.6.1 Unified Parkinson Disease Rating Scale (MDS-UPDRS) 28

8.6.1.1 MDS-UPDRS Restrictions 29

8.6.2 Vital Signs 29
8.6.3 Montreal Cognitive Assessment (MoCA) 29
8.6.4 Parkinson Disease Non-Motor Symptom Scale (NMSS) 29
8.6.5 Parkinson's Non-Motor Symptom Questionnaire (NMSQuest) 29
8.6.6 Electrocardiogram (ECG) Procedure 29
8.6.7 Lumbar Puncture (LP) 30
8.6.7.1 Lumbar Puncture Restrictions 30
8.7 CSF Biomarkers Analysis 30
8.8 Routine and Study Related Blood Collection Analysis 30
8.9 Study Related Urine Collection Analysis 31
9. Assessments and Follow Up 31
9.1 Screening Visit 1 (within 60 days of Day 1, pre-treatment phase) 31
9.2 Screening Visit 2 (within 60 days of Day 1, pre-treatment phase) 32
9.3 Telephone Visit 1 (within 3 days of Visit 2) 32
9.4 Treatment Phase - Visit 3 Day 1 (within 60 days of Visit 1 & 2) 32
9.5 Telephone Visit 2 (within 3 days of Visit 3, day 1) 33
9.6 Telephone Visit 3 (pre-dose escalation 2, within 3 days before day 8) 33
9.7 Dose Escalation 2 (day 8) 33
9.8 Treatment Phase - Visit 4 (within 3 days after dose Escalation 2, day 8) 33
9.9 Telephone Visit 4 (pre-dose escalation 3, within 3 days before day 15) 33
9.10 Dose Escalation 3 (day 15) 33
9.11 Telephone Visit 5 (post-dose escalation 3, within 3 days after day 15) 33
9.12 Telephone Visit 6 (pre-dose escalation 4, within 3 days before day 22) 34
9.13 Dose Escalation 4 (day 22) 34
9.14 Telephone Visit 7 (post-dose escalation 4, within 3 days after day 22) 34
9.15 Telephone Visit 8 (pre-dose escalation 5, within 3 days before day 29) 34
9.16 Dose Escalation 5 (day 29) 34
9.17 Telephone Visit 9 (post-dose escalation 5, within 3 days after day 29) 34
9.18 Treatment Phase - Visit 5 - Day 93 (+/- 14 days, treatment phase) 34
9.19 Telephone Visit 10 (within 5 days of Visit 5) 35
9.20 Treatment Phase - Visit 6 - Day 186 (+/- 14 days) 35
9.21 Telephone Visit 11 (within 5 days of Visit 6, day 186) 35
9.22 End of Study/Early Termination Visit Follow Up Phase – Visit 7, Day 35
279 (+/- 30 days) 35
9.23 Optional Telephone Visit 12, if participant consents to optional 36
lumbar puncture (within 5 days of Visit 7, Day 279) 36
9.24 Unscheduled Visits 36

10. Investigational Medicinal Product (IMP) – Ambroxol 36
10.1 Name and Description of IMP 36
10.2 Mode of Action 36
10.3 Source of IMP, Manufacture, Distribution and Storage 36
10.4 Preparation and Labelling of Investigational Medicinal Product 37
10.5 Storage and Handling of IMP at Site 37
10.6 Accountability of IMP(s) 37
10.7 Treatment Schedule and Dosing Instructions 37
10.8 Missed Dosing Instruction 38
10.9 Overdose of Trial Medication 38
10.10 Dispensing 39
10.11 IMP Discontinuations 39
10.12 Participant Stopping Criteria 39
10.13 Intra-participant Dose Escalation Stopping Criteria 39
10.14 Participant Termination Criteria 39
10.15 Participant Withdrawal and Termination 40
10.16 Assessment of Compliance and Adherence 40
10.17 Post-trial IMP Arrangement 40
10.18 Definition of End of Trial 40
11. Recording and Reporting of Adverse Events and Reactions 40
11.1 Definitions 40
11.2 Recording Adverse Events 41
11.3 Assessments of Adverse Events 41
11.4 Severity 41
11.5 Causality 42
11.6 Expectedness 43
11.7 Expected Procedural Related Events 43
11.8 Seriousness 43
11.9 Procedures for Recording and Reporting Serious Adverse Events 43
11.10 Reporting SUSARs 46
11.11 Development Safety Update Reports (DSUR) 46
11.12 Pregnancy 46
11.13 Reporting Urgent Safety Measures and other Safety Events 46
11.14 Notification of Deaths 47
11.15 Notification of Serious Breaches to GCP and/or the protocol 47
11.16 Clinical Incidental Findings 47
12. Data Management and Quality Assurance 47

12.1 Confidentiality....47
12.2 Data Collection Tools and Source Document Identification....47
12.3 Completing Case Report Forms....48
12.4 Trial Management Group (TMG)....48
12.5 Data Handling and Analysis....48
13. Statistical Considerations 48
13.1 Primary Outcomes....48
13.2 Secondary Outcomes....48
13.3 Sample Size and Recruitment....48
13.4 Sample Size Calculation....49
13.5 Statistical Analysis Plan (SAP)....49
14. Record Keeping and Archiving 49
14.1 Direct Access to Source Data/Documents....49
15. Ethics and Regulatory Requirements 49
16. Monitoring Requirement for the Trial 50
17. Finance 50
18. Insurance 50
19. Publication Policy 50
20. References 51
21. Appendices Appendix 1 - Schedule of Events 55
Appendix 2 – UK Parkinson disease society brain bank clinical diagnostic criteria ... 57

List of Abbreviations

ADL	Activities in Daily Life
AE	Adverse Event
ALI	Acute Lung Injury
APR	Annual Progress Report
AR	Adverse Reaction
ARDS	Acute Respiratory Distress Syndrome
BBB	Blood-brain barrier
BP	Blood Pressure
CA	Competent Authority
CI	Chief Investigator
CNS	Central Nervous System
COMT	Catechol-O-methyltransferase
CRF	Case Report Form
CSF	Cerebrospinal Fluid
CTA	Clinical Trial Authorisation
CTCAE	Common Terminology Criteria for Adverse Events
CTIMP	Clinical Trial of Investigational Medicinal Product
DIBD	Developmental International Birth Date
DDC	Decarboxylase
DMC	Data Monitoring Committee
DSUR	Development Safety Update Report
EC	European Commission
ECG	Electrocardiogram
ELISA	Enzyme-linked Immunosorbent Assay
EMEA	European Medicines Agency
EU	European Union
EudraCT	European Clinical Trials Database
FDA	Food and Drug Administration
FSH	Follicle Stimulating Hormone
FPFV	First Participant First Visit
GBA	Gaucher gene
GCase	Glucocerebrosidase
GCP	Good Clinical Practice
GD	Gaucher Disease
HED	Human Equivalent Dose
HR	Heart Rate
HPLC	High Performance Liquid Chromatography
HTA	Human Tissue Authority
ICF	Informed Consent Form
IgA	Immunoglobulin A
IMP	Investigational Medicinal Product
ISF	Investigator Site File
ITU	Intensive Therapy Unit
LGC	Laboratory of the Government Chemist
L-DOPA	Levodopa
LP	Lumbar Puncture
LPLV	Last Participant Last Visit
LWENC CRF	Leonard Wolfson Experimental Neurology Centre Clinical Research Facility

MA	Marketing Authorisation
MAO-B	Monoamine Oxidase B (MAO-B)
MDS-UPDRS	Movement Disorder Society (MDS-sponsored) revision of the Unified Parkinson's Disease Rating Scale
MoCA	Montreal Cognitive Assessment
MHRA	Medicines and Healthcare products Regulatory Agency
NHNN	National Hospital for Neurology and Neurosurgery
NHS R&D	National Health Service Research & Development
NMSS	Parkinson's Disease Non Motor Symptom Scale
NMS Quest	Parkinson's Non Motor Symptom Questionnaire
NOAEL	No-observed-adverse-effect-level
PD	Parkinson disease
PI	Principal Investigator
PIC	Participant Identification Centre
PIS	Participant Information Sheet
PR	Pulse Rate
QA	Quality Assurance
QC	Quality Control
QP	Qualified Person
R&D	Research & Development
REC	Research Ethics Committee
RFH	Royal Free London NHS Foundation Trust (Royal Free Hospital)
RR	Respiratory Rate
SAR	Serious Adverse Reaction
SAE	Serious Adverse Event
SAP	Statistical Analysis Plan
SDV	Source Document Verification
SoDA	Summary of Drug Arrangements
SOP	Standard Operating Procedures
SmPC	Summary of Product Characteristics
SSA	Site Specific Assessment
SSRIS	Selective Serotonin Uptake Inhibitors
SUSAR	Suspected Unexpected Serious Adverse Reaction
T	Temperature
TID	ter in diē (three times a day)
TMF	Trial Master File
TMG	Trial Management Group
TNFα	Tumour Necrosis Factor alpha
UCLH	University College London Hospitals NHS Foundation Trust
UCL	University College London

1. Summary

This is a proof of concept investigational medicinal product (IMP) study to investigate the safety, tolerability and pharmacodynamic effects of the Glucocerebrosidase (GCase) enhancing chaperone ambroxol in Parkinson disease (PD) participants with and without Gaucher gene (*GBA*) mutations. Twenty participants will be recruited in total, 10 with a *GBA* positive status and 10 with a *GBA* negative status. Each participant will orally self-administer ambroxol (ambrosan) at 5 intra-participant dose escalations at 60 mg TID (day 1-7), 120 mg TID (day 8-14), 180 mg TID (day 15-28), 300 mg TID (day 22-28) and 420 mg TID (day 29-186).

Participants will be subjected to clinical and laboratory assessments to assess the safety, tolerability and pharmacodynamic effects of ambroxol on blood and CSF biomarkers. Lumbar punctures will be performed to assess the penetration of ambroxol into the cerebrospinal fluid (CSF) through the blood-brain barrier (BBB) in the central nervous system (CNS) and the effects of ambroxol on GCase activity. Biomarkers and GCase activity markers will be assessed through the visits.

Each participant will undergo 7 hospital visits and up to 12 telephone visits. Hospital visits will include 2 screening appointments within 60 days of the Day 1 hospital visit (at which participants will receive the first dose of ambroxol), followed by a visit within 3 days of the second dose escalation, Day 93 (month 3), Day 186 (month 6) and an end of study follow up/early termination visit at Day 279 (month 9). Participants will receive a telephone call to record any adverse events in between hospital visits after screening visit 2, visit 3, the first dose, between 1-3 days before and after each dose escalation (day 1, 8, 15, 22 and 29), between day 93 and day 186, between day 186 and day 279 and a final telephone call within 5 days of the day 279 hospital visit if an optional lumbar puncture (LP) is undertaken. Please refer to Appendix 1 for the detailed schedule of events.

2. Background and Rationale

PD is the second most common neurodegenerative disease after Alzheimer's disease with a prevalence of around 0.1% (Hobson et al., 2005; Schrag et al., 2000). The current lifetime risk for PD in the UK is estimated at 4% (Schapira et al., 2014) There is currently no therapy to slow down the progression in PD. Numerically the most important risk factor for the development of PD is the presence of the *GBA* mutation (Sidransky and Lopez, 2012).

GCase is a lysosomal hydrolase enzyme responsible for the degradation of the sphingolipid (class of lipids containing a backbone of sphingoid bases, a set of aliphatic amino alcohols that includes sphingosine) waste product glucosylceramide. Inherited mutations of *GBA* in the homozygous state cause Gaucher disease (GD), a lysosomal storage disorder in which the GCase substrate glucosylceramide accumulates in visceral organs, with a variety of clinical phenotypes (Grabowski, 2008). A mutation in the *GBA* gene in the heterozygous state represents carrier status that until recently was thought to be asymptomatic. It is presently estimated that approximately 7-10% of UK PD patients have *GBA* mutations and a mutation in heterozygous or homozygous form increases the risk of PD 20-30 fold (McNeill et al., 2012; Neumann et al., 2009; Sidransky et al., 2009; Winder-Rhodes et al., 2013). Penetrance of the PD phenotype amongst *GBA* carriers

(homozygous and heterozygous) is approximately between 10-30%.(Anheim et al., 2012; McNeill et al., 2012; Sidransky and Hart, 2012).

Idiopathic *GBA*-negative PD and *GBA*-positive PD are indistinguishable clinically, pharmacologically and pathologically except *GBA*-positive PD has a slightly earlier age of onset and a small increase in the frequency of age-adjusted cognitive impairment (Alcalay et al., 2012; Chahine et al., 2013; Setó-Salvia et al., 2012). The mechanisms by which *GBA* mutations result in PD are not yet understood, however there is evidence that that it may be connected with reduced levels of GCase enzyme activity. For instance, transgenic mice carrying mutations associated with PD and idiopathic PD brains/CSF appear to demonstrate reduced GCase activity (Gegg et al., 2012; Parnetti et al., 2014; Sardi et al., 2013). Furthermore, it has been demonstrated that there is a reciprocal relationship between GCase activity and levels of alpha synuclein, the cytosolic protein which is the main constituent of Lewy bodies, the pathological hallmark of PD (Cullen et al., 2011; Mazzulli et al., 2011). Alpha synuclein has also been shown directly to interact with *GBA in vitro*, an interaction which is reduced in mutant forms of the protein (Yap et al., 2013a; Yap et al., 2013b). There is evidence from cell and animal models that there is a reciprocal relationship between GCase and alpha-synuclein (Schapira and Gegg, 2013). Thus, elevation of GCase activity reduces alpha-synuclein levels and increased alpha-synuclein lowers GCase activity. This has raised the possibility that modulation of GCase enzyme activity may represent a putative neuroprotective mechanism in PD.

Current treatment for patients with GD includes enzyme replacement therapy however this treatment does not cross the BBB, making it unsuitable as for a CNS condition such as PD. There is extensive research on the use of pharmacological chaperones to enhance lysosomal *GBA* activity (Parenti, 2009). Screening of a library of 1,040 Food and Drug Administration (FDA) approved drugs identified ambroxol, a respiratory decongestant (which is a surfactant stimulant, a mucokinetic and secretagogue) as a potential chaperone for *GBA*. Our laboratory has confirmed that ambroxol has a distinct mode of action which enhances GCase activity in human *GBA* fibroblasts (McNeill et al., 2014).

Ambroxol hydrochloride appears to facilitate the refolding of misfolded mutant *GBA* protein at the endoplasmic reticulum, allowing effective trafficking to the lysosome (Bendikov-Bar et al., 2013; Westbroek et al., 2011). Preliminary *in vivo* studies from our own laboratory have confirmed ambroxol administration is able to increase GCase activity levels within mouse brains. The proposed design is a proof of principle trial to demonstrate that ambroxol is able to reach the CNS and increase GCase activity in human CSF. If confirmed, it will pave the way for a larger phase III trial to test clinically and biochemically the neuroprotective effect of ambroxol in *GBA*-positive and idiopathic *GBA*-negative PD. If positive, this would represent the first neuroprotective treatment in PD to date.

2.1 Ambroxol Justification of Route of Administration and Dosage

Ambroxol (brand name: ambrosan) has a long history of use in humans, which documents its very low level of toxicity. For several decades now ambroxol has held market authorisation (MA) by the European Medicines Agency (EMEA) and in several countries across Europe for over-the-counter indications at doses up to 120 mg/day. There have been a number of clinical trials of ambroxol at a variety of doses. In one small study 12 participants with type I GD took 150 mg daily in two divided doses for 6 months with minimal side effects. This study showed some improvement in clinical features of GD, particularly in those with the lowest weight. Briefly, all but one participant experienced an increase in their platelet count (average 11.8% increase) and all but two experienced a reduction in splenic volume (average 2.7% reduction), both positive features in GD (Zimran et al., 2013). A recent study in Japan treated 5 neuronopathic GD patients with ambroxol at an escalating dose of 3mg/kg/day to 5mg/kg/dose for 12-31 months. (Narita et al., 2013). The study reported anecdotal improvements in myoclonus and seizure frequency. The only adverse event reported was mild hyperuricaemia (Narita et al., 2016). Another study treated 12 healthy volunteers with 500 mg or 1g twice a day for 5 days and revealed no adverse effects (Oosterhuis et al., 1993). A number of studies have demonstrated use of a 1g daily dose on (Intensive Therapy Unit) ITU in participants with acute respiratory distress syndrome (ARDS) for short periods. In one study 32 participants with Acute Respiratory Distress Syndrome (ARDS) were administered 40 mg/kg ambroxol in 4 daily divided doses for up to 10 days with no adverse effects (Baranwal et al., 2015) whilst a recent meta-analysis of 508 participants enrolled in studies using high dose ambroxol (15-20mg/kg/day) for short periods (3-7 days) to treat ARDS and acute lung injury (ALI) did not reveal any significant adverse effects (Wu et al., 2014). A further trial assesses the efficacy of ambroxol (1g dose administered every 12 hours for a maximum of 48 hours) prophylactically in pregnant women to prevent infantile ARDS. The study demonstrated its safety to pregnant woman and the unborn child (Visani and Daniotti, 1992). Pre-clinically, the oral administration of ambroxol in GD mice has demonstrated its wide distribution and chaperone activity in tissues, including the brain, and lack of acute toxicity (Luan et al., 2013).

Ambroxol has a low index for acute toxicity. No toxicological effect on target organs has been detected to date. Four week intravenous toxicity studies with ambroxol in rats (4, 16 and 64 mg/kg/day) and in dogs (45, 90 and 120 mg/kg/day (infusion 3 h/day) showed no severe local or systemic toxicity including at histopathology. All adverse effects were reversible. Ambroxol was neither embryotoxic nor teratogenic when tested at oral doses up to 3000 mg/kg/day in rats and up to 200 mg/kg/day in rabbits. The fertility of male and female rats was not affected up to 500 mg/kg/day. Genotoxicity studies in vitro (Ames and chromosome aberration test) and in vivo (mouse micronucleus test) did not reveal any mutagenic potential of ambroxol. Ambroxol did not show any tumorigenic potential in carcinogenicity studies in mice (50, 200 and 800 mg/kg/day) and rats (65, 250 & 1000 mg/kg/day) when treated with a dietary admixture for 105 and 116 weeks, respectively.

The Human Equivalent Dose (HED) based on the no-observed-adverse-effect-level (NOAEL) derived allometric scaling of animal pharmacokinetics to human exposure was: 150 mg/kg/day in mice (following 4 weeks treatment), corresponding to a HED of 12.1 mg/kg; 50 mg/kg/day in rats (52 and 78 weeks), corresponding to a HED of 8.0 mg/kg; 40 mg/kg in rabbits (26 weeks), corresponding to a HED of 12.9 mg/kg; 10 mg/kg in dogs (52 weeks), corresponding to a HED of 5.5 mg/kg). The above NOAELs together

with the available clinical doses tested in humans provide toxicity cover for the dose chosen in this study at 5 intra-participant dose escalations at 60 mg TID (day 1-7), 120 mg TID (day 8-14), 180 mg TID (day 15-28), 300 mg TID (day 22-28) and 420 mg TID (day 29-186).

2.2 Scientific Rationale for Ambroxol 1260 mg TID maximum dose

To date, ambroxol has been used as an over-the-counter cough linctus for peripheral effects on respiratory mucosa. It has obtained market authorisations in a number of European markets. It has been shown in a number of studies to be safe for long term administration (Narita et al., 2016 & Zimran et al., 2013). This includes the study of Narita et al., 2016 where participants were given ambroxol in excess (up to 1.3 g total daily dose) of the maximum dose of 1.26g total daily dose proposed in our protocol, and for periods of up to 4 years. A number of other studies have used a total daily dose in excess of 1g for up to 10 days including one in the third trimester of pregnancy (Wu et al., 2014 & Baranwal et al., 2015).

The scientific basis for the use of ambroxol in PD has been established with a number of mechanistic studies (see above). CNS penetration has been confirmed by increased glucocerebrosidase activity and protein levels in animal studies using rodents and 5mM ambroxol in drinking water, available *ad libitum*. A limited non-human primate study was performed using 22.5 or 100 mg/day ambroxol by gavage for 28 days. Brain glucocerebrosidase was increased with the 100 mg but not the 22.5 mg dose. On an mg/kg basis, these doses correspond to 4.5 mg/kg/day and 20 mg/kg/day respectively in the non-human primates, approximately equivalent to 315 mg/day and 1400 mg/day in humans. These data are currently in submission for publication.

Narita et al studied the effects of ambroxol in 5 patients with Gaucher disease (homozygous glucocerebrosidase mutations) with daily doses of 1200 mg for 4 years, 1200 mg for 3 years, 1300 mg for 3 years, 625 mg for 0.5 years and 375 mg for 2 years and measured serum and CSF ambroxol concentrations and substrate (glucosylsphingosine) levels over these periods. No significant adverse events were noted. Ambroxol was detectable at 0.06-0.83uM/L in CSF, these levels are anticipated to be detectable in our assays, but the lower range is at the limit of detection. There was a reduction in substrate levels indicating enhanced glucocerebrosidase activity in the CSF, although this was not measured.

We have reviewed the above animal, non-human primate and patient data and selected the 1260 mg/day dose to ensure efficacy in target engagement in the CNS, and detection in the CSF. The data indicate that a lower dose may not penetrate the CNS, or induce an effect necessary to enhance glucocerebrosidase activity.

Because there have been a small number of case reports of anaphylaxis associated with the IMP (and to ensure against other unreported or idiosyncratic side effects) we have opted for a regimen which includes 5 intra-participant dose escalations, 7 dedicated study visits, 10 telephone visits over the 6 months of IMP administration. For the same reason safety blood tests, clinical examination, ECGs and recording of AEs and concomitant medication are included at each study visit. Because a mild uricosuric effect was also reported in a recent clinical trial and in previous studies, serum urate will also be monitored (Narita et al., 2016 & Oosterhuis et al., 1993). The protocol also includes carefully thought-out and stringent stopping, withdrawal, termination and an additional intra-participant dose

escalation stopping criteria to ensure participant safety is not compromised throughout the course of the study.

3. Assessment and management of risk

The table below summarises the IMP and procedural risks, frequencies and mitigations of the IMP. Based on the above information the classification of risk assessment is Type B, somewhat higher than the risk of standard medical care.

IMP	Potential risk	Risk Frequency	Risk Management
Ambroxol (Brand name: Ambrosan) administration and sustained release (refer to SmPC)	1. Rash<br>2. Urticaria.	Rare (≥1/10,000 to <1/1,000)	Evidence or history of sensitivity to study medication, ambroxol or its excipients in the opinion of the investigator will be excluded from the study.
	3. Anaphylactic reactions including anaphylactic shock, angioedema, pruritus and other hypersensitivity	Not known based on information available	
	4. Nausea	Common (≥1/100 to <1/10)	Any adverse events will be recorded and serious adverse events will be reported.<br><br>Participants may be withdrawn from the study if they experience SAE, and will be based on the opinion of the PI.
	5. Dyspepsia<br>6. Vomiting<br>7. Diarrhoea<br>8. Abdominal pain	Uncommon (≥1/1,000 to <1/100)	
	9. Stevens-Johnson syndrome (new skin or mucosal lesions and non-specific influenza-like prodromes like e.g. fever, aching body, rhinitis, cough and sore throat)	Very rare (<1/10,000)	

	10. Lyell's syndrome (new skin or mucosal lesions and non-specific influenza-like prodromes like e.g. fever, aching body, rhinitis, cough and sore throat).	Very rare (<1/10,000)	

**Procedural**	**Potential risk**	**Risk Frequency**	**Risk Management**
Lumbar Puncture	1. Mild Headache	<10%	Participants with spinal malformation will be excluded from the study.<br><br>Please note any adverse events will be recorded and serious adverse events will be reported.<br><br>Participants may be withdrawn from the study if they experience SAE, and will be based on the opinion of the PI.
	2. Bleeding at wound site, localised back pain, oedema, rash and urticarial	Rare	
	3. Low pressure headache	Very Rare	
	4. Infection, damage to the nerves around the spine and bleeding into the CSF	Very Rare	
Venepuncture	5. Bruising, infection at wound site	Minimal Risk (very low risk)	
ECG	6. Rash from electrodes	Minimal Risk (very low risk)	

4. Objectives

4.1 Primary Objective

- To assess the central nervous system (CNS), cerebrospinal fluid (CSF) penetration and binding to GCase of ambroxol by the parameters outline (modulation of GCase activity & ambroxol level) at 5 intra-participant dose escalations from day 1 to day 186 at 60 mg TID (day 1-7), 120 mg TID (day 8-14), 180 mg TID (day 15-21), 300 mg TID (day 22-28) and 420 mg TID (day 29-186).

4.2 Secondary Objectives

- To assess the safety and tolerability of the Glucocerebrosidase (GCase) modulating chaperone ambroxol in Parkinson disease participants with and without Gaucher gene (*GBA*) mutation at 5 intra-participant dose escalations from day 1 to 186.
- To measure the pharmacodynamic effects of ambroxol on GCase activity in blood and CSF following ambroxol oral administration at 5 intra-participant dose escalations from day 1 to 186.
- To quantify the effect of ambroxol on biomarkers of Parkinson and neurodegeneration at 5 intra-participant dose escalations from day 1 to 186.

4.3 Exploratory Objective

- To explore whether ambroxol leads to improvement in cognitive performance, Montreal Cognitive Assessment (MoCA) and the motor and non-motor features of Parkinson disease from day 1 to day 186.
- To explore whether ambroxol leads to improvement in Parkinson Disease non-motor symptom assessment scale (NMSS) and non-motor symptom questionnaire (NMSQuest) from day 1 to day 186.

5. Trial Design

5.1 Overall Design

This is a single-centre, proof of concept, open-labelled IMP study to evaluate the safety, tolerability and pharmacodynamics of ambroxol in participants with PD. Participants will be recruited and administer ambroxol at 5 intra-participant dose escalations at 60 mg TID (day 1-7), 120 mg TID (day 8-14), 180 mg TID (day 15-21), 300 mg TID (day 22-28) and 420 mg TID (day 29-186).

Participants will undergo clinical assessments (see Appendix 1, schedule of events) lumbar punctures, venepuncture, biomarker, blood analysis and cognitive assessment throughout the course of the study.

6. Study Settings

6.1 Site Selection

This study will be conducted at the Leonard Wolfson Experimental Neurology Centre Clinical Research Facility (LWENC CRF), which is based at the National Hospital for Neurology and Neurosurgery (NHNN), and is part of the University College London Hospitals NHS Foundation Trust (UCLH). The Royal Free London NHS Foundation Trust (RFH) will act as a Participant Identification Centre (PIC).

6.2 Study Setting

All participant assessments will be performed at the LWENC CRF which is based at the NHNN.

6.3 Study Duration

Each participant will receive 5 intra-participant dose escalations at 60 mg TID (day 1-7), 120 mg TID (day 8-14), 180 mg TID (day 15-21), 300 mg TID (day 22-28) and 420 mg TID (day 29-186) for the duration of 6 months and will be followed up for 9 months from administration of the first dose of ambroxol at day 1.

6.4 Chief/Principal Investigator's (CI/PI) Qualification and Agreement

The CI/PI must be willing to sign the UCL's Standard Operating Procedures (SOP) and an Investigator Agreement to comply with the study protocol (confirming their specific roles and responsibilities related to the trial, and that their site is willing and able to comply with the requirements of the trial). This includes confirmation of appropriate qualifications, familiarity with the appropriate use of any investigational medicinal product (IMP), agreements to comply with principles of GCP, to permit monitoring and audit as necessary at the site, and to maintain documented evidence of all staff at the site that has been delegated significant trial related duties.

7. Eligibility Criteria

7.1 Study Population

All participants who are recruited to this study shall have a confirmed diagnosis of PD. There will be no exceptions to eligibility requirements and all participants must fulfil the inclusion criteria.

Twenty PD participants (10 *GBA* positive & 10 *GBA* negative) will be enrolled into the study. If enrolled and participants prematurely discontinue the study, up to 4 additional participants (2 *GBA* positive and 2 *GBA* negative) may be enrolled as replacement participants.

7.2 Participant Selection

A confirmed diagnosis of PD at any time will be based on the opinion of the PI or a PI-delegated clinician. The clinical history will be reviewed, and the participants will be examined physically and neurologically to ensure each participant meet the UK brain bank criteria for PD diagnosis (see Appendix 2) and Hoehn and Yahr criteria (confirmed staged between I – III, inclusive). Please see table below for the Hoehn and Yahr staging of PD:

Hoehn and Yahr Staging of Parkinson Disease Classification

Stage	Criteria
**I**	Signs and symptoms on one side only<br>Symptoms mild<br>Symptoms inconvenient but not disabling<br>Usually presents with tremor of one limb<br>Friends have noticed changes in posture, locomotion and facial expression
**II**	Symptoms are bilateral<br>Minimal disability<br>Posture and gait affected
**III**	Significant slowing of body movements<br>Early impairment of equilibrium on walking or standing

	Generalised dysfunction that is moderately severe
**IV**	Severe symptoms<br>Can still walk to a limited extent<br>Rigidity and bradykinesia<br>No longer able to live alone<br>Tremor may be less than earlier stages
**V**	Cachectic stage<br>Invalidism complete<br>Cannot stand or walk<br>Requires constant nursing care

7.3 Participant Inclusion Criteria

1. Male or female;
2. Age ≥ 40 and ≤ 80 years of age;
3. Confirmed diagnosis of Parkinson disease at any time; and Hoehn and Yahr criteria, confirmed staged between I – III, inclusive;
4. Able and willing to provide informed consent prior to any study related assessments and procedures at screening visit 1;
5. Capable of complying with all study procedures, including fasting lumbar puncture;
6. Willing to provide a blood sample for screening genomic for Parkinson Disease related *DNA* analysis and/or consent to Investigators obtaining and using participants previous *DNA* results if applicable;
7. Willing and able to self-administer oral ambroxol medication, from day 1 to 186 (at 60 mg TID (day 1-7), 120 mg TID (day 8-14), 180 mg TID (day 15-21), 300 mg TID (day 22-28) and 420 mg TID (day 29-186));
8. Able to travel to the participating study site;
9. A female participant is eligible to participate if she is of:
   - Non-childbearing potential defined as pre-menopausal females with a documented tubal ligation or hysterectomy; or postmenopausal defined as 12 consecutive months of spontaneous amenorrhea, at least 6 weeks post-surgical bilateral oophorectomy (with or without hysterectomy) or post tubal ligation. In questionable cases, menopausal status will be confirmed by demonstrating levels of follicle stimulating hormone (FSH) 25.8 – 134.8 IU/L and oestradiol < 201 pmol/l at entry.
   - Women of child-bearing potential must use accepted contraceptive methods (listed below), and must have a negative serum at screening visit 1 and urine pregnancy tests at subsequent visits if applicable. An additional pregnancy test will be performed, and results obtained, prior to administration of the first dose of ambroxol.

   Accepted contraception methods:
   - True abstinence: When this is in line with the preferred and usual lifestyle of the participant. [Periodic abstinence (e.g., calendar, ovulation, symptothermal, post-ovulation methods) and withdrawal are not acceptable methods of contraception].

   Contraceptive Methods with a Failure Rate of < 1%:
   - Oral contraceptive, either combined or progestogen alone;
   - Injectable progestogen;
   - Implants of levonorgestrel;
   - Estrogenic vaginal ring;
   - Percutaneous contraceptive patches; -

- Intrauterine device (IUD) or intrauterine system (IUS) that meets the <1% failure rate as stated in the product label;

Please note:

- All male and female participants of child bearing potential must agree with their partners to use double-barrier birth control or abstinence while participating in the study and for 2 weeks following the last dose of the study drug.
- Participants may continue to take PD medications including glutamate antagonists, anticholinergics, dopamine agonists, Levodopa (L-DOPA and decarboxylase (DDC) inhibitor), Monoamine oxidase B (MAO-B) inhibitors catechol-O-methyltransferase (COMT) inhibitors, beta blockers, selective serotonin uptake inhibitors (SSRIS), tricyclic antidepressants (TCAs) and indomethacin.

7.4 Participant Exclusion Criteria

Participants are excluded from participating in this study if 1 or more of the following criteria are met:

1. Current treatment with anticoagulants (e.g. warfarin) that might preclude safe completion of the lumbar puncture and in the opinion of the Investigator;
2. Current use of investigational medicinal product or participation in another interventional clinical trial or who have done so within 30 days prior to the first dose in the current study;
3. Exposure to more than three investigational medicinal products within 12 months prior to the first dose in the current study;
4. Confirmed dysphagia that would preclude self-administration of ambroxol up to 7 tablets TID for the duration of day 1 to day 186);
5. Significant known lower spinal malformations or other spinal abnormalities that would preclude lumbar puncture;
6. History of known sensitivity to the study medication, ambroxol or its excipients (lactose monohydrate, granulated microcrystalline cellulose, copovidone and magnesium stearate) in the opinion of the investigator that contraindicates their participation;
7. History of known rare hereditary disorders of galactose intolerance, Lapp lactase deficiency or glucose-galactose malabsorption;
8. Evidence or history of hypersensitivity to lidocaine or its derivatives;
9. History of drug abuse or alcoholism in the opinion of the Investigator that would preclude participation in the study;
10. Donation of blood (one unit or 350 ml) within three months prior to receiving the first dose of the study drug;
11. Pregnant or breastfeeding;
12. All participants of child bearing potential in the opinion of the Investigator that would preclude participation in the study and who do not agree to use double-barrier birth control or abstinence while participating in the study and for two weeks following the last dose of study drug;
13. Any clinically significant or unstable medical or surgical condition that in the opinion of the PI or PI-delegated clinician may put the participant at risk when participating in the study or may influence the results of the study or affect the participant’s ability to take part in the study, as determined by medical history, physical examinations, electrocardiogram (ECG), or laboratory tests.
    Such conditions may include:
    a) Impaired renal function
    b) Moderate/Severe hepatic impairment

c) A major cardiovascular event (e.g. myocardial infarction, acute coronary syndrome, decompensated congestive heart failure, pulmonary embolism, coronary revascularisation that occurred within 6 months prior to the screening visit.

7.5 Pregnancy and Contraception

Ambroxol is not recommended during pregnancy as it crosses the placental barrier and when breast feeding as ambroxol is excreted in breast milk and participants that are pregnant or breastfeeding will be excluded from the study.

All male and female participants of child bearing potential must agree with their partners to use double-barrier birth control or abstinence whilst participating in the study and for 2 weeks following the last dose of the study drug as specified in the inclusion and exclusion criteria.

In addition, regular pregnancy testing will be performed for female of potential child-bearing status during the study if applicable.

8. Trial Procedures

8.1 Recruitment

Potential participants will be approached by their treating clinician (who in the case of this study may also be the CI/PI, Co-Investigator or PI-delegated clinician) during routine standard of care outpatient appointments at the RFH or the NHNN. Potential participants will be given a participant information sheet (PIS) and the opportunity to ask questions before deciding whether or not to take part.

8.2 Participant Identification Centres (PIC)

The RFH will be the PIC and will refer all potential participants to NHNN at UCLH. The CI/PI, Co-Investigator and PI-delegated clinicians at the RFH have UCLH honorary contracts.

8.3 Informed Consent Process

It is the responsibility of the CI/PI Investigator, or a PI-delegated clinician to obtain written informed consent from each participant prior to participation in the trial, following adequate explanation of the aims, methods, anticipated benefits and potential hazards of the trial.

The person taking consent will be GCP trained, suitably qualified and experienced, and will have been delegated this duty by the CI/ PI on the Staff Signature and Delegation of Tasks.

"Adequate time" must be given for consideration by the participant before taking part. Consent will be sought at least 24 hours after being given the study documentation. It must be recorded in the medical notes when the PIS have been given to the participant.

A copy of the signed informed consent form will be given to the participant. The original signed form will be retained in the trial file at site and a copy placed in the medical notes. Participants are under no obligation to enter the trial and can withdraw at any time during the trial without providing a reason.

The PIS and consent form will be reviewed and updated if necessary throughout the trial (e.g. where new safety information becomes available) and participants will be re-consented as appropriate.

8.4 Re-screening of Participant

Participant may repeat the screening procedures once to be eligible for the study if they have:

- Used anticoagulant therapy apixaban (Eliquis), dabigatran (Pradaxa) rivaroxaban (Xarelto) and Warfarin within 30 days prior to Screening Visit 2 (rescreening may be performed 14 days or more after discontinuation of anticoagulant therapy).
- An abnormal test value may normalise, in the opinion of the Investigator (rescreening may be performed once test values is within normal range).

8.5 Informed consent for Genotyping/disclosure of previously held genotyping

The study will recruit an equal number of *GBA*-positive and *GBA*-negative participants. Participants will be genotyped for GBA and LRRK2 (a relatively common genetic risk factor for PD) at screening visit 1. All participants who have not undertaken genotyping in an NHS certified laboratory will be genotyped. The genotyping for LRRK2 is essential to prevent the confounding effect of this genetic form of Parkinson's disease on our results.

All participants will be asked whether they wish to be told the results of their genotyping and counselled accordingly as part of standard of care procedures by researchers with referral onto local genetic counselling services if required.

8.6 Study Procedure Assessments

The CI/PI or PI-delegated member of the research team will conduct each assessment highlighted in Appendix 1, schedule of events.

The following clinical and non-clinical assessments will be conducted during the study: informed consent, medical history, physical examination, neurological examination, movement disorder society (MDS-sponsored) revision of the Unified Parkinson's Disease Rating Scale United Parkinson Disease's Rating Scale (MDS-UPDRS), Montreal Cognitive Assessment (MoCA), non-motor symptom (NMS) questionnaire, non-motor symptom scale (NMSS), weight, height, electrocardiogram (ECG), vital signs, lumbar puncture, concomitant review, adverse events review, dispensing and collecting IMP packaging, urine collection, dosing (day 1 to 186), IMP compliance and dosing instructions, blood and CSF biomarkers and routine blood samples.

8.6.1 Unified Parkinson Disease Rating Scale (MDS-UPDRS)

The MDS-UPDRS is made up of the following:

Part I: evaluation of mentation, behaviour, and mood.
Part II: self-evaluation of the activities of daily life (ADLs) including speech, swallowing, handwriting, dressing, hygiene, falling, salivating, turning in bed, walking, and cutting food.

Part III: clinician-scored monitored motor evaluation.
Part IV: complications of therapy.
Part V: Hoehn and Yahr staging of severity of Parkinson disease.
Part VI: Schwab and England ADL scale

The scales provide an impartial measure of disease progression in established PD and are validated as such (Wenning et al., 2004). The assessment is evaluated by interview and clinical observation. The duration is 30-60 minutes and will be administered in the morning of each study visit.

8.6.1.1 MDS-UPDRS Restrictions

On screening visit 2, visit 3 (day 1), visit 5 (day 93), visit 6 (day 186) and visit 7 (day 279), participants must refrain from taking their standard of care PD medication (standard of care procedure) until they have undertaken the UPDRS assessment in the morning, and will be advised accordingly by the PI or PI-delegated member of the research team when to administer their standard of care medication during the study visits which will be also be highlighted in the participant dosing instruction booklet.

8.6.2 Vital Signs

Vital signs include the measurements of a blood pressure (BP), pulse rate (PR), respiration rate (RR) and temperature (T) must be performed as single reads. Prior to the blood pressure assessment the participant should remain rested in a seated position for 10 minutes. The BP measurement should be recorded whilst the participant is in a seated position. If results are abnormal, repeat testing can be performed and assessed by the CI/PI or PI-delegated clinician.

8.6.3 Montreal Cognitive Assessment (MoCA)

The MoCA is a screening test for cognitive impairment that spans the visuospatial/executive, naming, memory, attention, language, abstraction, delayed recall and orientation domains will be recorded at selected study visits. It is a multi-domain cognitive battery validated in established PD and the score ranges from 0 to 30 points (Hoops et al., 2009). The duration is 10-20 minutes and must be administered preferably in the morning of selected study visits.

8.6.4 Parkinson Disease Non-Motor Symptom Scale (NMSS)

The NMSS is a validated 30 question scale designed to detect the non-motor features of Parkinson’s disease.(Martinez-Martin et al., 2015). The duration of the scale is 10-20 minutes and can be administered at any time on specified study visits.

8.6.5 Parkinson’s Non-Motor Symptom Questionnaire (NMSQuest)

The NMSQuest is a validated 30 question scale designed to detect the non-motor features of Parkinson’s disease. (Chaudhuri et al., 2006) The duration of the scale is 5-10 minutes and can be administered at any time on specified study visits.

8.6.6 Electrocardiogram (ECG) Procedure

A standard 12-lead ECG single read will be conducted at selected study visits. Prior to the assessment the participant should be rested in a supine position for 10 minutes.

If any ECG finding that is judged by the site investigator as a clinically significant change (worsening) compared with Visit 3, Day 1 (baseline, pre-dose) value will be considered an

adverse event, recorded on the source documentation and the CRF. If results are abnormal repeat testing may be performed and checked by the CI/PI or PI-delegated clinician.

8.6.7 Lumbar Puncture (LP)

The LP procedure will be undertaken using aseptic technique as part of a morning assessment. The CSF collection should be performed using a small calibre atraumatic needle (e.g. 22 or 24 gauge Sprotte needle). During the procedure local anaesthetic will be used. The CSF will be collected using the traditional gravity drip method. Up to 20 mL (5-6 teaspoons) of CSF will be taken for analysis and storage, please refer to the latest laboratory manual for sample collection, processing transport and analysis.

After the LP procedure the participant must lay down (rest) for a minimum of 1 hour. The participant will be contacted by the research team within 5 days of the procedure to assess any adverse events or concomitant medication and advise the participant accordingly as part of the telephone visit. Participants will be advised to remain well hydrated, and to perform light activities for the next 48 hours.

8.6.7.1 Lumbar Puncture Restrictions

There are no restrictions to the study except the use of warfarin and other anticoagulants as described in the inclusion criteria and the requirements for a fasting CSF samples. Each participant must provide a fasting CSF sample for each LP procedure at the following visits: screening visit 2, visit 6 (day 186) and an optional LP at visit 7 (day 279). Participants are required to fast for a minimum of 4 hours before the LP procedure (no food except water).

8.7 CSF Biomarkers Analysis

CSF samples will be collected and analysed, please refer to the latest version of the AiM-PD laboratory manual. All sample collection, processing and analysis are within European Union.

**CSF Biomarker (Up to 20 mL)**	
**ELISA (enzyme-linked immunosorbent assay) antibody panel antibody panel**	
α-synuclein<br>t-tau<br>p-tau<br>β-amyloid<br>Neurofilament light chain<br>Neurogranin<br>Lysomal marker panel	Interleukin-8, Macrophage Inflammatory protein 1α<br>Monocyte chemoattractant protein-1 (MCP-1)<br>TNF-a (tumour necrosis factor alpha)
**Enzyme activity panel**	
Glucocerebrosidase (GCase)<br>Chitotriosidase	
CSF ambroxol levels	
CSF Cell Count	
CSF glucosylceramide	
CSF glucocerebrosidase protein	

8.8 Routine and Study Related Blood Collection Analysis

Blood samples will be collected and analysed, please refer to the latest version of the AiM-PD laboratory manual.

**Blood Biomarker (From 25-80 mL per visit)***

ELISA antibody panel antibody panel	
α-synuclein<br>t-tau<br>p-tau<br>β-amyloid<br>Neurofilament light chain<br>Neurogranin<br>Lysosomal marker panel	Interleukin-8<br>Macrophage Inflammatory protein 1α<br>Monocyte chemoattractant protein-1 (MCP-1)<br>TNF-α (tumour necrosis factor alpha)
**Enzyme activity panel**	
Glucocerebrosidase<br>Chitotriosidase<br>Lysosomal glucocerebrosidase	
**Blood ambroxol levels**	
**Routine blood panels**<br>Haematology Panel<br>Biochemistry Panel<br>Coagulation Panel<br>Serum uric acid	
**Serum Blood Pregnancy Test (if applicable)**	
***GBA* Genotyping**<br>*GBA* Genetic Analysis<br>LRRK2 Genetic Analysis	

*Please refer the latest version of laboratory manual for detailed volumes of blood required for each visit.

8.9 Study Related Urine Collection Analysis

Pregnancy urine samples will be collected and analysed, please refer to the latest version of the AiM-PD laboratory manual.

Urine
Urine pregnancy test*<br>*For female participants of child bearing potential, if applicable
Urine collection (Up to 50 mL)

Urine samples will be stored for future proteomics, metabolomics and lipidomics studies related to the PD biomarker detection and also High Performance Liquid Chromatography (HPLC) assays to detect ambroxol levels in the urine.

9. Assessments and Follow Up

All participants will undertake study related procedures at specified visits, please see Appendix 1 for detailed schedule of events.

9.1 Screening Visit 1 (within 60 days of Day 1, pre-treatment phase)

- Informed consent
- Medical history
- Physical and neurological examinations

- Screening genotyping (*GBA* and LRRK2), if applicable
- Vital signs (HR, BP, RR and T)
- Height and weight (morning assessment)
- ECG
- For female participants of child bearing potential, serum pregnancy test if applicable
- Routine blood collection
- Blood collection enzyme activity & ELISA antibody panels
- Blood ambroxol collection
- Adverse event review
- Concomitant medication review

9.2 Screening Visit 2 (within 60 days of Day 1, pre-treatment phase)

- Physical and neurological examinations
- MDS-UPDRS (morning assessment)
- Screening inclusion/exclusion criteria
- Vital signs (HR, BP, RR and T)
- Weight (morning assessment)
- ECG
- For female participants of child bearing potential, urine pregnancy test if applicable
- Routine blood collection
- Lumbar puncture (up to 20 mL) (morning assessment)
- CSF collection enzyme activity & ELISA antibody panels
- CSF ambroxol collection
- Adverse events review
- Concomitant medication review

9.3 Telephone Visit 1 (within 3 days of Visit 2)

- Adverse events review
- Concomitant medication review

9.4 Treatment Phase - Visit 3 Day 1 (within 60 days of Visit 1 & 2)

- Physical and neurological examinations
- MDS-UPDRS (morning assessment and pre-dose)
- Vital signs (HR, BP, RR and T), (pre-dose and 1 hour post IMP administration and prior to discharge)
- Weight (morning assessment)
- ECG (pre-dose and 1 hour post for each IMP administration)
- For female participants of child bearing potential, urine pregnancy test if applicable (pre-dose)
- Urine collection (morning assessment and pre-dose)
- Dosing on site, IMP Administration - Day 1 to day 186 (inclusive)
- Dispensing 3 months' supply of IMP to participant
- MoCA Cognitive assessment (morning assessment and pre-dose)
- NMSS and NMS Questionnaire
- Adverse events review
- Concomitant medication review

- Participants are instructed to self-administer 60 mg TID at day 1 to day 7
- Participant can be discharged and instructed to take the remaining daily dose(s) at home as soon as all other protocol procedures are completed.

9.5 Telephone Visit 2 (within 3 days of Visit 3, day 1)

- IMP compliance and dosing instructions
- Adverse events review
- Concomitant medication review

9.6 Telephone Visit 3 (pre-dose escalation 2, within 3 days before day 8)

- IMP compliance and dosing instructions
- Adverse events review
- Concomitant medication review

9.7 Dose Escalation 2 (day 8)

- Participants are instructed to self-administer 120 mg TID on day 8 to day 14.

9.8 Treatment Phase - Visit 4 (within 3 days after dose Escalation 2, day 8)

- Physical and neurological examinations
- Vital signs (HR, BP, RR and T)
- Weight (morning assessment)
- For female participants of child bearing potential, urine pregnancy test if applicable (pre-dose)
- Urine collection (morning assessment and pre-dose)
- Routine blood collection
- Blood collection
- Collecting IMP package (pill count only)
- Blood collection enzyme activity & ELISA antibody panels
- Blood ambroxol collection
- Urine collection (morning assessment and pre-dose)
- Dosing on site, IMP Administration - Day 1 to Day 186 (inclusive) if applicable
- IMP compliance and dosing instructions
- Adverse events review
- Concomitant medication review

9.9 Telephone Visit 4 (pre-dose escalation 3, within 3 days before day 15)

- IMP compliance and dosing instructions
- Adverse events review
- Concomitant medication review

9.10 Dose Escalation 3 (day 15)

- Participants are instructed to self-administer 180 mg TID on day 15 to day 21

9.11 Telephone Visit 5 (post-dose escalation 3, within 3 days after day 15)

- IMP compliance and dosing instructions
- Adverse events review
- Concomitant medication review

9.12 Telephone Visit 6 (pre-dose escalation 4, within 3 days before day 22)

- IMP compliance and dosing instructions
- Adverse events review
- Concomitant medication review

9.13 Dose Escalation 4 (day 22)

- Participants are instructed to self-administer 300 mg TID on day 22 and continue to day 28

9.14 Telephone Visit 7 (post-dose escalation 4, within 3 days after day 22)

- IMP compliance and dosing instructions
- Adverse events review
- Concomitant medication review

9.15 Telephone Visit 8 (pre-dose escalation 5, within 3 days before day 29)

- IMP compliance and dosing instructions
- Adverse events review
- Concomitant medication review

9.16 Dose Escalation 5 (day 29)

- Participants are instructed to self-administer 420 mg TID on day 29 to day 186

9.17 Telephone Visit 9 (post-dose escalation 5, within 3 days after day 29)

- IMP compliance and dosing instructions
- Adverse events review
- Concomitant medication review

9.18 Treatment Phase - Visit 5 - Day 93 (+/- 14 days, treatment phase)

- Physical and neurological examinations
- MDS-UPDRS (morning assessment and pre-dose)
- Vital signs (HR, BP, RR and T)
- Weight (morning assessment)
- For female participants of child bearing potential, urine pregnancy test if applicable, if applicable (pre-dose)
- Routine blood collection
- Blood collection enzyme activity & ELISA antibody panels
- Blood ambroxol collection
- Urine collection (morning assessment)
- Dosing on site, IMP Administration - Day 1 to Day 186 (inclusive) if applicable
- Dispensing 3 months' supply of IMP
- Collecting IMP packaging

- Adverse events review
- Concomitant medication review

9.19 Telephone Visit 10 (within 5 days of Visit 5)

- IMP compliance and dosing instructions
- Adverse events review
- Concomitant medication review

9.20 Treatment Phase - Visit 6 - Day 186 (+/- 14 days)

- Physical and neurological examinations
- MDS-UPDRS (morning assessment)
- Vital signs (HR, BP, RR and T)
- Weight (morning assessment)
- ECG
- Lumbar puncture (up to 20 mL) morning assessment
- For female participants of child bearing potential, urine pregnancy test if applicable (pre-dose)
- Routine blood collection
- Blood collection enzyme activity & ELISA antibody panels
- CSF collection enzyme activity & ELISA antibody panels
- Blood ambroxol collection
- CSF ambroxol collection
- Urine collection (morning assessment)
- Dosing on site, IMP Administration - Day 1 to Day 186 (inclusive), last day of dose
- Collecting IMP packaging
- MoCA cognitive assessment (morning assessment)
- NMSS and NMS Questionnaire
- Adverse events review
- Concomitant medication review

9.21 Telephone Visit 11 (within 5 days of Visit 6, day 186)

- Adverse events review
- Concomitant medication review

9.22 End of Study/Early Termination Visit Follow Up Phase – Visit 7, Day 279 (+/- 30 days)

- Physical and neurological examinations
- MDS-UPDRS (morning assessment)
- Vital signs (HR, BP, RR and T)
- Weight (morning assessment)
- ECG
- *Optional lumbar puncture – if participant agrees to optional LP they will be followed up by a telephone call to assess any AEs and a concomitant medical review within 5 days of the visit.
- CSF collection enzyme activity & ELISA antibody panels if participant consents to the optional lumbar puncture procedure (if participant consents to LP)
- *CSF ambroxol collection only if participant consents to optional lumbar puncture procedure (if participant consents to LP)

- **Collecting IMP packaging, if early termination visit
- For female participants urine pregnancy test unless menopausal status is confirmed (if applicable)
- Blood collection enzyme activity, ELISA antibody panels
- Routine blood collection
- Urine collection (morning assessment)
- Collecting IMP packaging
- Adverse events review
- Concomitant medication review

9.23 Optional Telephone Visit 12, if participant consents to optional lumbar puncture (within 5 days of Visit 7, Day 279)

- Adverse events review
- Concomitant medication review

9.24 Unscheduled Visits

Unscheduled visits may be performed at any time during the study, whenever necessary to assess or to follow up on adverse events (AEs) or serious adverse events (SAEs) at the participant's request, or as deemed necessary by the Investigator.

If a participant wishes to withdraw from the study early, they will be invited to attend visit 7, day 279 (end of study visit/early termination visit) at that point prior to withdrawing from the study.

10. Investigational Medicinal Product (IMP) – Ambroxol

10.1 Name and Description of IMP

Ambroxol hydrochloride 60mg tablets (Brand Name: Ambrosan).

Each tablet contains 60 mg of ambroxol hydrochloride. The appearance is almost round and is cross-scored and the diameter 9.5 mm. Ambroxol contains the following excipients: lactose monohydrate, granulated microcrystalline cellulose, copovidone and magnesium stearate.

10.2 Mode of Action

Ambroxol increases the clearance of mucus in the respiratory system by enhancing pulmonary surfactant production and stimulating ciliary activity improving mucociliary clearance and expectoration, and eases cough-like symptoms. Ambroxol decreases bronchial hyper-reactivity by increasing the secretion of immunoglobulin A (IgA) in bronchial mucus; it also shows antioxidant activity.

10.3 Source of IMP, Manufacture, Distribution and Storage

Ambroxol hydrochloride 60mg tablets will be sourced by PRO.MED.CS Praha a.s. (ambroxol marketing authorisation holder) that are based in the Czech Republic within Europe.

10.4 Preparation and Labelling of Investigational Medicinal Product

PRO.MED.CS Praha a.s. will send IMP shipments to the RFH Pharmacy Production Unit (MIA (IMP) holder) for re-packaging and re-labelling in accordance with EU GMP Annex 13. The latter will provide Qualified Person (QP) certification and will send the re-packaged and re-labelled IMP to the NHNN Pharmacy department for dispensing and storage for the study.

10.5 Storage and Handling of IMP at Site

The IMP does not require any special storage requirements and will be stored at ambient conditions during transportation and while at sites. Guidance on handling temperature excursions is provided in the Summary of Drug Arrangements (SoDA) document.

10.6 Accountability of IMP(s)

The Sponsor will provide guidance on the destruction of unused investigational product (eg, at the site). If destruction is authorised to take place at the study site, the investigator must ensure that the materials are destroyed in compliance with applicable environmental regulations, institutional policy, and any special instructions provided by the Sponsor and all destruction must be adequately documented. Further guidance will be provided in the SoDA. IMP shipping, receipt, storage and accountability arrangements for the site will be described in the Summary of Drug Arrangements (SoDA) document.

10.7 Treatment Schedule and Dosing Instructions

Participants will be instructed to self-administer 5 intra-participant dose escalations at 60 mg TID (day 1-7), 120 mg TID (day 8-14), 180 mg TID (day 15-21), 300 mg TID (day 22-28) and 420 mg TID (day 29-186). The study drug should be administered with or without a meal and rinsed down with sufficient amount of fluid for each daily morning, afternoon and evening in accordance to the instructions provided by the Investigator.

On the first day of dosing, day 1, the study drug should be self-administered by the participant at the site as described above. The participant will undergo vital signs performed an hour after the first dose. Participants can be discharged and instructed to take the remaining daily dose(s) at home as soon the other protocol procedures are completed and will undergo vital signs clinical assessment before being discharged.

At all subsequent schedule visits, participants may self-administer the study medication on site and will be instructed to take the remaining daily dose(s) if applicable by the Investigator accordingly (especially on lumbar puncture visits) and will be discharged after the first dose if applicable.

On screening visit 2, visit 3 (day 1), visit 5 (day 93), visit 6 (day 186) and visit 7 (day 279), participants should not administer their standard of care PD medication until they have undertaken the UPDRS assessment and will be instructed by the PI or PI-delegated member of the research team, this is a standard of care procedure and the Investigator will instruct each participant.

During the course of the study, if a participant misplaces, incorrectly stores or uses the IMP outside the dosing instructions provided, the participant must notify the PI/PI-delegated member of the research team to assess whether replacements of the IMP is required.

Dose Escalation Number	Day	Ambroxol (Ambrosan) Dose and Frequency	Ambroxol (Ambrosan) Daily Dose (mg/day)	Number of 60 mg Tablets per Dose	Total Number of 60 mg Tablets Per Day
1	1-7	60 mg TID	180	1	3
2	8-14	120 mg TID	360	2	6
3	15-21	180 mg TID	540	3	9
4	22-28	300 mg TID	900	5	15
5	29-186	420 mg TID	1260	7	21

Dose reductions are not planned but may be permitted. The PI may allow a dose reduction if adverse symptoms have emerged since dose increase that are considered to be due to the increase, are not SAEs and do not require the participant to be withdrawn, and in the opinion of the PI and participant to warrant a reduction to the last tolerated dose level.

10.8 Missed Dosing Instruction

In the event of a missed dose the outstanding tablet(s) should be taken as soon as it becomes known to the participant a dose had been missed (morning, afternoon or evening dose). If a morning, afternoon or evening dose is missed, the participant to proceed to the subsequent dose that day e.g. a participant misses the morning dose they should proceed to the afternoon dose and evening dose that day and should continue their dosing regimen the subsequent day.

Participants should try and ensure every attempt should be made to ensure consistency in the time of study drug administration during the course of the study.

10.9 Overdose of Trial Medication

Should a participant take an overdose that exceeds the recommended dose described in the protocol and experiences any adverse event(s) that may relate to an overdose the participant must inform the PI or PI-delegated member of the research team immediately who will then follow up on their medical care as needed. It will be the opinion of the PI to decide whether the participant should be withdrawn from the study or whether the IMP should be reintroduced.

Each overdose will be recorded and the Sponsor will be notified of the overdose on the deviation log. Overdoses will be observed by the research team being instructed by the participant and pill counts on specific study visits, Visit 5 (day 93), Visit 6 (day 186) and the early termination visit, if applicable.

10.10 Dispensing

The NHNN Pharmacy will dispense the IMP in accordance to the SoDA. Each participant will be dispensed 3 months' supply of IMP at visit 3 (day 1) and visit 5 (day 93) to last at least until the next study visit, along with instructions to use.

10.11 IMP Discontinuations

Based on the known good safety profile of ambroxol no clinical significant adverse reactions are expected in this study. However individual participants may be discontinued from treatment at any time during the study (alternatively the study may be terminated prematurely) in the events of unexpected, significant or unacceptable adverse event such that, in the opinion of Investigators would put them or other trial participant safety at risk.

10.12 Participant Stopping Criteria

The stopping criteria refer to a participant stopping administration of the IMP. The withdrawal of a participant from the study may include the following reasons:

- Participants withdrawal of consent
- Non-compliance with the protocol
- Occurrence of an AE or change in medical status or an incidental finding that leads the Investigator concerned about the participant's welfare
- Pregnancy during the study
- Inability to continue, loss to follow-up
- The termination of the study by the Sponsor

If a participant is withdrawn from the study, they will be asked to undergo the early termination visit (visit 7, day 279) study procedures if applicable.

If enrolled participants prematurely discontinue the study or do not comply with the protocol, additional participants may be enrolled as replacement participants and assigned to the same treatment sequence at the discretion of the Principal Investigator. A maximum of two GBA positive and two GBA negative participants may be enrolled as replacement participants.

10.13 Intra-participant Dose Escalation Stopping Criteria

Dose escalation will be halted and reviewed by the TMG if:

- a serious adverse event (SAE) occurs in 1 or more participants receiving ambroxol that is considered at least possibly related to study drug;
- two or more severe adverse events occur in a group for subjects receiving ambroxol that are considered at least possibly related to study drug;
- A formal decision will be sent to the sponsor to restart dose escalation (if deemed appropriate). A participant already on the dose escalation regime will continue if no adverse reactions have occurred.

10.14 Participant Termination Criteria

The termination criteria refer to a participant stopping the study drug and all assessments and visits. The Investigator can terminate participants from the study for any of the following reasons:

- Participants withdrawal of consent
- Inability to continue, loss to follow-up
- Death

Participants who terminate the study for any reason will not be allowed to return to the study drug or study assessments at any time.

10.15 Participant Withdrawal and Termination

Participants have the right to withdraw from the study at any time for any reason, without prejudice to their standard of care medical care. Additionally, the Investigator may discontinue a participant from the IMP or terminate the participant from the study.

10.16 Assessment of Compliance and Adherence

The participants will be instructed to return all (used and un-used) IMP packages at each clinic visit for compliance check. The returned IMP will be collected from the CI/PI or the PI-delegated member of the research team from the participant at Visit 5 (day 93) and Visit 6 (day 186). The returned IMP will be counted to assess compliance in addition to telephone visits 2-10 where IMP compliance will be assessed.

10.17 Post-trial IMP Arrangement

There will be no arrangements for participants to receive additional IMP on study completion.

10.18 Definition of End of Trial

The end of trial is the date of the last participant's last visit or last participant's last telephone follow up visit.

11. Recording and Reporting of Adverse Events and Reactions

Collection, recording and reporting of adverse events (including serious and non-serious events and reactions) to the sponsor will be completed according to the sponsor's SOP (JRO/INV/S05).

11.1 Definitions

Term	Definition
**Adverse Event (AE)**	Any untoward medical occurrence in a patient or clinical trial subject administered a medicinal product and which does not necessarily have a causal relationship with this treatment.
**Adverse Reaction (AR)**	Any untoward and unintended response in a participant to an investigational medicinal product which is related to any dose administered to that participant.<br>*This includes medication errors, uses outside of protocol (including misuse and abuse of product)*
**Serious Adverse Event (SAE), Serious Adverse**	Any adverse event, adverse reaction or unexpected adverse reaction, respectively, that:

**Reaction (SAR) or Unexpected Serious Adverse Reaction**	results in death,<br>is life-threatening*,<br>requires hospitalisation or prolongation of existing hospitalisation**,<br>results in persistent or significant disability or incapacity, or<br>consists of a congenital anomaly or birth defect
*A life- threatening event, this refers to an event in which the participant was at risk of death at the time of the event; it does not refer to an event which hypothetically might have caused death if it were more severe.<br>** Hospitalisation is defined as an in-patient admission, regardless of length of stay. Hospitalisation for pre-existing conditions, including elective procedures do not constitute an SAE.	
**Important Medical Event**	These events may jeopardise the subject or may require an intervention to prevent one of the above characteristics/consequences. Such events should also be considered 'serious'.
**Unexpected adverse reaction**	An adverse reaction the nature and severity of which is not consistent with the information about the medicinal product in question set out:<br>(a) in the case of a product with a marketing authorization, in the summary of product characteristics for that product,<br>(b) in the case of any other investigational medicinal product, in the investigator's brochure relating to the trial in question.
**Suspected Unexpected Serious Adverse Reaction (SUSAR)**	An unexpected adverse reaction which is also categorised as serious.

11.2 Recording Adverse Events

All adverse events will be recorded in the medical records in the first instance with clinical symptoms and accompanied with a simple, brief description of the event, including dates as appropriate.

All adverse events will be recorded and assessed for seriousness in the medical records and CRF following consent (First Participant First Visit - FPFV) until (Last Participant Last Visit - LPLV), visit 7 and if the participant consents to the optional LP at visit 7, day 279 they will receive a telephone call (telephone call 12) to record AEs within 5 days of this visit.

11.3 Assessments of Adverse Events

Each adverse event will be assessed for the following criteria:

11.4 Severity

Category	Definition

**Mild**	The adverse event does not interfere with the participant's daily routine, and does not require intervention; it causes slight discomfort
**Moderate**	The adverse event interferes with some aspects of the participant's routine, or requires intervention, but is not damaging to health; it causes moderate discomfort
**Severe**	The adverse event results in alteration, discomfort or disability which is clearly damaging to health

11.5 Causality

The assessment of relationship of adverse events to the administration of IMP is a clinical decision based on all available information at the time of the completion of the case report form. The following categories will be used to define the causality of the adverse event:

Category	Definition
Definitely:	There is clear evidence to suggest a causal relationship, and other possible contributing factors can be ruled out.
Probably:	There is evidence to suggest a causal relationship, and the influence of other factors is unlikely
Possibly	There is some evidence to suggest a causal relationship (e.g. the event occurred within a reasonable time after administration of the trial medication). However, the influence of other factors may have contributed to the event (e.g. the participant's clinical condition, other concomitant events).
Unlikely	There is little evidence to suggest there is a causal relationship (e.g. the event did not occur within a reasonable time after administration of the trial medication). There is another reasonable explanation for the event (e.g. the participant's clinical condition, other concomitant treatments).
Not related	There is no evidence of any causal relationship.
Not Assessable	Unable to assess on information available.

11.6 Expectedness

Category	Definition
Expected	An adverse event which is consistent with the information about the IMP listed in section 4.8 of the SmPC (Reference Safety Information - RSI)
Unexpected	An adverse event which is not consistent with the information (RSI) about the IMP listed in the Investigator Brochure (or SmPC if Licensed IMP)*

* this includes listed events that are more frequently reported or more severe than previously reported.

The reference document to be used to assess expectedness against the IMP is highlighted in the SmPC for Ambrosan (Ambroxol) (Marketing Authorisation holdersPRO.MED.CS Praha a.s., Telčská 1, 140 00 Praha 4, Czech Republic)

11.7 Expected Procedural Related Events

Please refer to section on 4 for expected procedural side effects.

11.8 Seriousness

All events will be assessed for seriousness as defined for a SAE in section 11.1

11.9 Procedures for Recording and Reporting Serious Adverse Events

All serious adverse events (SAEs/SARs/SUSARs) will be recorded in the medical records and the CRF, and the sponsor's AE log. The CRF AE log of SAEs will be reported to the sponsor at least once a year.

All SAEs will be recorded from consent FPFV to LPLV. The LP will be optional to participants if they agree to undergo an LP at visit 7, day 279 the CSF sample will be sent for analysis and they will receive a safety telephone call within 5 days of visit 7 to record AEs.

All SAEs must be recorded on a serious adverse event (SAE) form. The CI/PI or designated individual will complete the sponsor's SAE form and the form will be preferably emailed to the Sponsor SAE@ucl.ac.uk and/or faxed on 020 3108 2312, within 24 hours of them becoming aware of the event. The CI/PI will respond to any SAE queries raised by the sponsor as soon as possible.

Completed SAE forms must be sent within 24 hours of becoming aware of the event to the Sponsor

Email forms to SAE@ucl.ac.uk

and/or

Fax forms to 020 3108 2312

v2.0 18.07.16

Flow Chart for SAE reporting

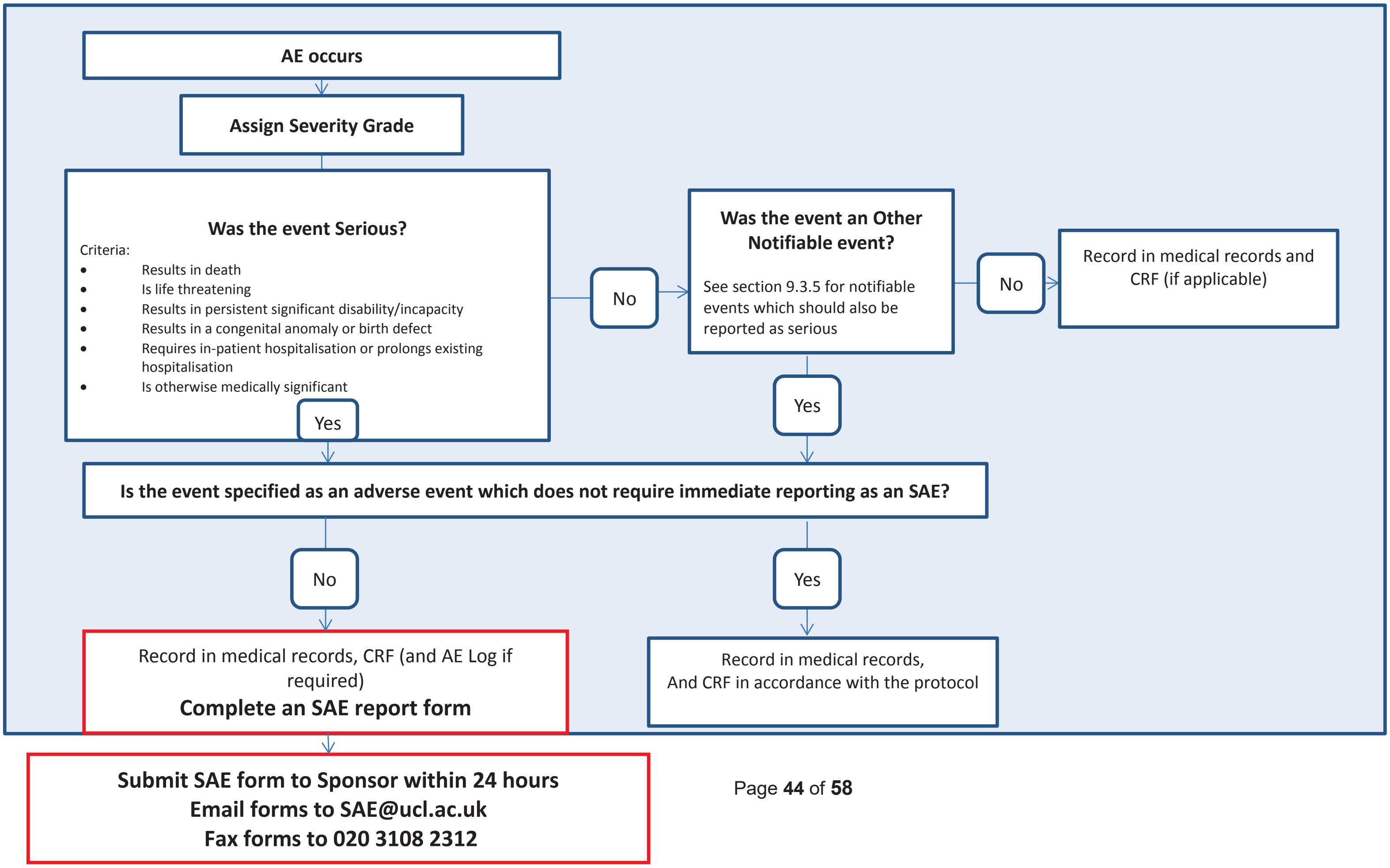

11.10 Reporting SUSARs

The sponsor will notify the main REC and Medicines and Healthcare products Regulatory Agency (MHRA) of all SUSARs. SUSARs that are fatal or life-threatening must be notified to the MHRA and REC within 7 days after the sponsor has learned of them. Other SUSARs must be reported to the REC and MHRA within 15 days after the sponsor has learned of them.

11.11 Development Safety Update Reports (DSUR)

The sponsor will provide the main REC and the MHRA with Development Safety Update Reports (DSUR) which will be written in conjunction with the trial team and the Sponsor's office. The report will be submitted within 60 days of the Developmental International Birth Date (DIBD) of the trial each year until the trial is declared ended. This will completed in accordance to the latest version of the Sponsor's SOP (JRO/INV/S17).

11.12 Pregnancy

Ambroxol crosses the placental barrier. Animal studies do not indicate direct or indirect harmful effects with respect to pregnancy, embryonal/foetal development, parturition or postnatal development. Extensive clinical experience after the 28th week of pregnancy including a clinical trial at high dose has shown no evidence of harmful effects on the foetus. The use of IMP containing ambroxol in pregnancy is not recommended; please refer to the latest version of the SmPC. Any pregnancy that occurs in a female trial subject during a clinical trial should be followed to termination or to term.

If a female participant or the female partner of a male participant becomes pregnant at any point during the trial, a completed trial specific Pregnancy Reporting Form will be emailed to the Sponsor SAE@ucl.ac.uk and/or faxed on **020 3108 2312,** within 24 hours of his /her becoming aware of the event in line with the Sponsors SOP (JRO/INV/S05/S06). The CI/PI will respond to any queries raised by the sponsor as soon as possible.

Completed Pregnancy Reporting Forms must be sent within **24 hours** of becoming aware of the event to the Sponsor
Email forms to SAE@ucl.ac.uk
and/or
Fax forms to 020 3108 2312

11.13 Reporting Urgent Safety Measures and other Safety Events

If any urgent safety measures are taken the CI/ PI shall immediately and in any event no later than 3 days from the date the measures are taken, give written notice to the MHRA, the relevant REC and Sponsor of the measures taken and the circumstances giving rise to those measures.

11.14 Notification of Deaths

All deaths, including deaths deemed unrelated to the IMP, if they occur earlier than expected will be reported to the sponsor.

11.15 Notification of Serious Breaches to GCP and/or the protocol

A “serious breach” is a breach which is likely to affect:

(a) the safety or physical or mental integrity of the participants of the trial; or
(b) the scientific value of the trial.

The sponsor of a clinical trial shall notify the licensing authority in writing of any serious breach of:

(a) the conditions and principles of GCP in connection with that trial; or (b) the protocol relating to that trial, as amended from time to time, within 7 days of becoming aware of that breach.

The sponsor will be notified immediately of any case where the above definition applies during the trial conduct. The sponsor’s SOP on the ‘Notification of violations, urgent safety measures and serious breaches’ will be followed.

11.16 Clinical Incidental Findings

Participants that undergo any of the clinical investigations during the course of the study which may result with a “clinically significant” finding will be excluded from taking part in the study and this judgement will be made by the treating clinician. All clinical incidental findings will be considered AEs and assessed as per section 11.0. Participants with such findings that may require further investigation will be informed of the findings by the Investigator and their GP will be informed, with the participant’s consent.

12. Data Management and Quality Assurance

12.1 Confidentiality

All data will be handled in accordance with the UK Data Protection Act 1998 and in accordance to GCP.

The Case Report Forms (CRFs) will not bear the participant’s name or other personal identifiable data. The participant’s trial identification number will be used for identification and this will be clearly explained to the participant in the PIS.

12.2 Data Collection Tools and Source Document Identification

Source documents would be defined in the source document list.

It is the responsibility of the Investigator to ensure the accuracy of all data entered in the CRFs. The delegation log will identify all those personnel with responsibilities for data collection and handling, including those who have access to the trial database.

12.3 Completing Case Report Forms

All CRFs must be completed and signed by staff that are listed on the site staff delegation log and authorised by the CI/PI. The CI/PI is responsible for the accuracy of all data reported in the CRF. The completed original CRFs will be kept within the LWENC CRF in the Trial Master File (TMF).

12.4 Trial Management Group (TMG)

The TMG will include the CI/PI and members of the research team. The TMG will be responsible for overseeing the trial. The group will meet regularly and the minutes will be recorded at each meeting.

12.5 Data Handling and Analysis

A trial specific data management SOP will be in place for the trial. This will contain details of the software to be used for the database, the process of database design, data entry, data quality checks, data queries, data security, database lock.

Where data are transferred electronically this will be in accordance with the UK Data Protection Act 1998 as well as UCL Information Security Policy and Trust Information Governance Policy. There will be a documented record of data transfer and measures in place for the recovery of original information after transfer.

13. Statistical Considerations

13.1 Primary Outcomes

The primary outcomes in this study are:

- To assess the central nervous system (CNS), cerebrospinal fluid (CSF) penetration by the parameters outlines (GCase activity, ambroxol level) from day 1 to day 186.

13.2 Secondary Outcomes

The secondary outcomes in this study are:

- CSF biomarkers of Parkinson disease and neurodegeneration
- CSF ambroxol levels
- Safety data, i.e.:
- Adverse events
- Laboratory safety data
- Physical and neurological examinations
- ECG

13.3 Sample Size and Recruitment

20 participants will be recruited: 10 *GBA* positive and 10 *GBA* negative. Participants who withdraw from the study prior to completion of the protocol will be replaced (up to two *GBA* positive and two *GBA* negative participants may be replaced).

13.4 Sample Size Calculation

The sample size has been selected by the chief investigator and is not based on any formal statistical calculation or consideration.

13.5 Statistical Analysis Plan (SAP)

Full details of all planned statistical analyses will be included in a written Statistical Analysis Plan which will be completed before the final study database is locked for analysis. The main focus of all analyses will be on the derivation of Bayesian 95% credible intervals for all endpoints and effects of interest, and statistical significance tests will be used sparingly. The Statistical Analysis Plan will specify those endpoints which will be analysed using statistical significance tests, and all final reports and publications will clearly identify and differentiate between pre-planned and post-hoc tests of statistical significance. All p-values will be adjusted to compensate for multiple statistical significance testing.

14. Record Keeping and Archiving

Archiving will be authorised by the Sponsor following submission of the end of study report. The CI is responsible for the secure archiving of essential trial documents and the trial database as per their trust policy. All essential documents will be archived for a minimum of 5 years after completion of trial and in adherence to the Sponsor's SOP (JRO/S21/02).

14.1 Direct Access to Source Data/Documents

The investigator/institution will permit trial-related monitoring, audits, REC review, and regulatory inspection, providing direct access to source data/documents, including medical notes. Trial participants will be informed during the informed consent discussion and will be asked to give consent.

15. Ethics and Regulatory Requirements

The sponsor will ensure that the trial protocol, PIS, consent form, GP letter and submitted supporting documents have been approved by the appropriate regulatory body (MHRA in UK) and an appropriate REC, prior to any participant recruitment. The protocol, all other supporting documents including and agreed amendments, will be documented and submitted for ethical and regulatory approval as required. Amendments will not be implemented prior to receipt of the required approval(s).

Before the site may be opened to recruit participants, the CI/PI or designee must receive NHS permission in writing from the Trust Research & Development (R&D). It is the responsibility of the CI/ PI or designee at each site to ensure that all subsequent amendments gain the necessary approvals, including NHS Permission (where required) at the site. This does not affect the individual clinician's responsibility to take immediate action if thought necessary to protect the health and interest of individual participants for reporting urgent safety measures).

An annual progress report (APR) will be submitted to the REC within 30 days of the anniversary date on which the favourable opinion was given, and annually until the trial is declared ended. The chief investigator will prepare the APR.

Within 90 days after the end of the trial, the CI/Sponsor will ensure that the main REC and the MHRA are notified that the trial has finished. If the trial is terminated prematurely, those reports will be made within 15 days after the end of the trial.

The CI will supply the Sponsor with a report of the clinical trial which complies with the format as defined by the EMA. This will then be uploaded to EudraCT for availability to the MHRA and a copy of the report will be submitted to the main REC, within 1 year after the end of the trial.

16. Monitoring Requirement for the Trial

A trial specific oversight and monitoring plan will be established for this study. The trial will be monitored in accordance with the agreed plan by UCL.

17. Finance

The funder of the study is Cure Parkinson's Trust.

18. Insurance

University College London holds insurance against claims from participants for injury caused by their participation in the clinical trial. Participants may be able to claim compensation if they can prove that UCL has been negligent. However, as this clinical trial is being carried out in a hospital, the hospital continues to have a duty of care to the participant of the clinical trial. University College London does not accept liability for any breach in the hospital's duty of care, or any negligence on the part of hospital employees. This applies whether the hospital is an NHS Trust or otherwise.

Participants may also be able to claim compensation for injury caused by participation in this clinical trial without the need to prove negligence on the part of University College London or another party. Participants who sustain injury and wish to make a claim for compensation should do so in writing in the first instance to the CI, who will pass the claim to the Sponsor's Insurers, via the Sponsor's office.

Hospitals selected to participate in this clinical trial shall provide clinical negligence insurance cover for harm caused by their employees and a copy of the relevant insurance policy or summary shall be provided to University College London, upon request.

19. Publication Policy

The Investigators will publish the findings from this study after all data has been collected, cleaned and analysed and the publication is complete.

20. References

Alcalay, R. N., Caccappolo, E., Mejia-Santana, H., Tang, M. X., Rosado, L., Orbe Reilly, M., et al. (2012). Cognitive performance of GBA mutation carriers with early-onset PD: the CORE-PD study. *Neurology*, *78*(18), 1434–1440. http://doi.org/10.1212/WNL.0b013e318253d54b

Anheim, M., Elbaz, A., Lesage, S., Dürr, A., Condroyer, C., Viallet, F., et al. (2012). Penetrance of Parkinson disease in glucocerebrosidase gene mutation carriers. *Neurology*, *78*(6), 417–420. http://doi.org/10.1212/WNL.0b013e318245f476

Baranwal, A. K., Murthy, A. S., & Singhi, S. C. (2015). High-dose Oral Ambroxol for Early Treatment of Pulmonary Acute Respiratory Distress Syndrome: an Exploratory, Randomized, Controlled Pilot Trial. *Journal of Tropical Pediatrics*. http://doi.org/10.1093/tropej/fmv033

Bendikov-Bar, I., Maor, G., Filocamo, M., & Horowitz, M. (2013). Ambroxol as a pharmacological chaperone for mutant glucocerebrosidase. *Blood Cells, Molecules & Diseases*, *50*(2), 141 145. http://doi.org/10.1016/j.bcmd.2012.10.007

Chahine, L. M., Qiang, J., Ashbridge, E., Minger, J., Yearout, D., Horn, S., et al. (2013). Clinical and Biochemical Differences in Patients Having Parkinson Disease With vs Without GBAMutations. *JAMA Neurology*, *70*(7), 852. http://doi.org/10.1001/jamaneurol.2013.1274

Chaudhuri, K.R., Martinez-Martin, P., Schapira, A.H., Stocchi, F., Sethi, K., Odin, P., Brown, R.G., Koller, W., Barone, P., MacPhee, G., Kelly, L., Rabey, M., MacMahon, D., Thomas, S., Ondo, W., Rye, D., Forbes, A., Tluk, S., Dhawan, V., Bowron, A., Williams, AJ and Olanow, C., (2006) International multicentre pilot study of the first comprehensive self-completed nonmotor symptoms questionnaire for Parkinson's disease: the NMSQuest study. *Mov Disorder* Jul;21(7):916-23

Cullen, V., Sardi, S. P., Ng, J., Xu, Y.-H., Sun, Y., Tomlinson, J. J., et al. (2011). Acid β-glucosidase mutants linked to Gaucher disease, Parkinson disease, and Lewy body dementia alter α-synuclein processing. *Annals of Neurology*, *69*(6), 940–953. http://doi.org/10.1002/ana.22400

Gegg, M. E., Burke, D., Heales, S. J. R., Cooper, J. M., Hardy, J., Wood, N. W., & Schapira, A. H. V. (2012). Glucocerebrosidase deficiency in substantia nigra of parkinson disease brains. *Annals of Neurology*, *72*(3), 455–463. http://doi.org/10.1002/ana.23614

Grabowski, G. A. (2008). Phenotype, diagnosis, and treatment of Gaucher's disease. *Lancet*, *372*(9645), 1263–1271. http://doi.org/10.1016/S0140-6736(08)61522-6

Hobson, P., Gallacher, J., & Meara, J. (2005). Cross-sectional survey of Parkinson's disease and parkinsonism in a rural area of the United Kingdom. *Movement Disorders*, *20*(8), 995–998. http://doi.org/10.1002/mds.20489

Hoops, S., Nazem, S., Siderowf, A. D., Duda, J. E., Xie, S. X., Stern, M. B., & Weintraub, D. (2009). Validity of the MoCA and MMSE in the detection of MCI and dementia in Parkinson disease. *Neurology*, *73*(21), 1738–1745. http://doi.org/10.1212/WNL.0b013e3181c34b47

Luan, Z., Li, L., Higaki, K., Nanba, E., Suzuki, Y., & Ohno, K. (2013). The chaperone activity and toxicity of ambroxol on Gaucher cells and normal mice. *Brain & Development*, *35*(4), 317–322. http://doi.org/10.1016/j.braindev.2012.05.008

Martinez-Martin, P., Chaudhuri, K. R., Rojo-Abuin, J. M., Rodriguez-Blazquez, C., Alvarez-Sanchez, M., Arakaki, T., et al. (2015). Assessing the non-motor symptoms of Parkinson's disease: MDS-UPDRS and NMS Scale. *European Journal of Neurology : the Official Journal of the European Federation of Neurological Societies*, *22*(1), 37–43. http://doi.org/10.1111/ene.12165

Mazzulli, J. R., Xu, Y.-H., Sun, Y., Knight, A. L., McLean, P. J., Caldwell, G. A., et al. (2011). Gaucher Disease Glucocerebrosidase and a Synuclein Form a BidirectionalPathogenic Loop in Synucleinopathies. *Cell*, *146*(1), 37–52. http://doi.org/10.1016/j.cell.2011.06.001

Narita, A., Kubota, N., Takayama, R., Takahashi, Y., Maegaki, Y., Suzuki, Y., & Ohno, K. (2013). Chaperone therapy for neuronopathic Gaucher disease. *Molecular Genetics and Metabolism*, *108*(2), S69.

Narita et al. (2016). Ambroxol chaperone therapy for neuronopathic Gaucher disease: A pilot study. *Annals of Clinical and Translational Neurology*, 3(3):200-215

McNeill, A., Duran, R., Hughes, D. A., Mehta, A., & Schapira, A. H. V. (2012). A clinical and family history study of Parkinson's disease in heterozygous glucocerebrosidase mutation carriers. *Journal of Neurology, Neurosurgery & Psychiatry*, *83*(8), 853–854. http://doi.org/10.1136/jnnp-2012-302402

McNeill, A., Magalhaes, J., Shen, C., Chau, K.-Y., Hughes, D., Mehta, A., et al. (2014). Ambroxol improves lysosomal biochemistry in glucocerebrosidase mutation-linked Parkinson disease cells. *Brain : a Journal of Neurology*, *137*(Pt 5), 1481–1495. http://doi.org/10.1093/brain/awu020

Neumann, J., Bras, J., Deas, E., O'Sullivan, S. S., Parkkinen, L., Lachmann, R. H., et al. (2009). Glucocerebrosidase mutations in clinical and pathologically proven Parkinson's disease. *Brain : a Journal of*

Neurology, *132*(Pt 7), 1783–1794. http://doi.org/10.1093/brain/awp044

Oosterhuis, B., Storm, G., Cornelissen, P. J., Su, C. A., Sollie, F. A., & Jonkman, J. H. (1993). Dose-dependent uricosuric effect of ambroxol. *European Journal of Clinical Pharmacology*, *44*(3), 237–241.

Parenti, G. (2009). Treating lysosomal storage diseases with pharmacological chaperones: from concept to clinics. *EMBO Molecular Medicine*, *1*(5), 268–279. http://doi.org/10.1002/emmm.200900036

Parnetti, L., Chiasserini, D., Persichetti, E., Eusebi, P., Varghese, S., Qureshi, M. M., et al. (2014). Cerebrospinal fluid lysosomal enzymes and α-synuclein in Parkinson's disease. *Movement Disorders*. http://doi.org/10.1002/mds.25772

Sardi, S. P., Clarke, J., Viel, C., Chan, M., Tamsett, T. J., Treleaven, C. M., et al. (2013). Augmenting CNS glucocerebrosidase activity as a therapeutic strategy for parkinsonism and other Gaucher-related synucleinopathies. *Proceedings of the National Academy of Sciences*, *110*(9), 3537–3542. http://doi.org/10.1073/pnas.1220464110

Schapira, A. H. V., & Gegg, M. E. (2013). Glucocerebrosidase in the pathogenesis and treatment of Parkinson disease. *Proceedings of the National Academy of Sciences of the United States of America*, *110*(9), 3214–3215. http://doi.org/10.1073/pnas.1300822110

Schapira, AH., Olanow, CW., Greenamyre, JT., Bezard, E. (2014). Slowing of neurodegeneration in Parkinson's disease and Huntington's disease: future therapeutic perspectives. *Lancet*, 384(9942) pp. 545-555.

Schrag, A., Ben-Shlomo, Y., & Quinn, N. P. (2000). Cross sectional prevalence survey of idiopathic Parkinson's disease and Parkinsonism in London. *BMJ (Clinical Research Ed.)*, *321*(7252), 21–22.

Setó-Salvia, N., Pagonabarraga, J., Houlden, H., Pascual-Sedano, B., Dols-Icardo, O., Tucci, A., et al. (2012). Glucocerebrosidase mutations confer a greater risk of dementia during Parkinson's disease course. *Movement Disorders*, *27*(3), 393–399. http://doi.org/10.1002/mds.24045

Sidransky, E., & Hart, P. S. (2012). Penetrance of PD in Glucocerebrosidase Gene Mutation Carriers. *Neurology*, *79*(1), 106 107. http://doi.org/10.1212/01.wnl.0000416261.29035.4c

Sidransky, E., & Lopez, G. (2012). The link between the GBA gene and parkinsonism. *Lancet Neurology*, *11*(11), 986–998. http://doi.org/10.1016/S1474-4422(12)70190-4

Sidransky, E., Nalls, M. A., Aasly, J. O., Aharon-Peretz, J., Annesi, G., Barbosa, E. R., et al. (2009). Multicenter analysis of glucocerebrosidase mutations in Parkinson's disease. *New England Journal of Medicine*,

361(17), 1651–1661. http://doi.org/10.1056/NEJMoa0901281

Wenning, G. K., Tison, F., Seppi, K., Sampaio, C., Diem, A., Yekhlef, F., et al. (2004). Development and validation of the Unified Multiple System Atrophy Rating Scale (UMSARS). *Movement Disorders*, *19*(12), 1391 1402. http://doi.org/10.1002/mds.20255

Westbroek, W., Gustafson, A. M., & Sidransky, E. (2011). Exploring the link between glucocerebrosidase mutations and parkinsonism. *Trends in Molecular Medicine*, *17*(9), 485–493. http://doi.org/10.1016/j.molmed.2011.05.003

Winder-Rhodes, S. E., Evans, J. R., Ban, M., Mason, S. L., Williams Gray, C. H., Foltynie, T., et al. (2013). Glucocerebrosidase mutations influence the natural history of Parkinson's disease in a community based incident cohort. *Brain : a Journal of Neurology*, *136*(Pt 2), 392 399. http://doi.org/10.1093/brain/aws318

Wu, X., Li, S., Zhang, J., Zhang, Y., Han, L., Deng, Q., & Wan, X. (2014). Meta-analysis of high doses of ambroxol treatment for acute lung injury/acute respiratory distress syndrome based on randomized controlled trials. *The Journal of Clinical Pharmacology*, *54*(11), 1199 1206. http://doi.org/10.1002/jcph.389

Visani, L., & Daniotti, S. (1992). SHORT SAFETY REPORT - Post marketing surveillance on tolerability of high dose ambroxol in pregnant women and newborns and on efficacy in preventing IRDS , 1–3. [Accessed 27.11.15 http://art45-paediatric-studies-docs.ema.europa.eu/GROUP%20A/Ambroxol%20hydrochloride/Ambroxol%20Hydrochloride_U93-0189_Synopsis.pdf]

Yap, T. L., Gruschus, J. M., Velayati, A., Sidransky, E., & Lee, J. C. (2013a). Saposin C protects glucocerebrosidase against α-synuclein inhibition. *Biochemistry*, *52*(41), 7161–7163. http://doi.org/10.1021/bi401191v

Yap, T. L., Velayati, A., Sidransky, E., & Lee, J. C. (2013b). Membrane bound α-synuclein interacts with glucocerebrosidase and inhibits enzyme activity. *Molecular Genetics and Metabolism*, *108*(1), 56–64. http://doi.org/10.1016/j.ymgme.2012.11.010

Zimran, A., Altarescu, G., & Elstein, D. (2013). Pilot study using ambroxol as a pharmacological chaperone in type 1 Gaucher disease. *Blood Cells, Molecules & Diseases*, *50*(2), 134–137. http://doi.org/10.1016/j.bcmd.2012.09.006

21. Appendices Appendix 1 - Schedule of Events

**STUDY PERIOD**	**SCREENING**			**TREATMENT**																**POST-TREATMENT**		
Visit	1	2		3				4									5		6		7[j]	
Day	-60	-60	V2+ 1-3 days	1	2-4	5-7	8	9-11	12-14	15	16-18	19-21	22	23-25	26-28	29	30-32	93	94-98	186	187-191	279	280-284[m]
Visit Window				<60 days of V1														+/-14		+/-14		+/-30	+4
Dose Escalation				1[f*]			2			3			4			5							
Visit Type (cl=clinic, t/c=telephone)	cl	cl	t/c1	cl	t/c2	t/c3		cl	t/c4		t/c5	t/c6		t/c7	t/c8		t/c9	cl	t/c10	cl	t/c11	cl	t/c12
Informed Consent	X						Patient self-administered dose increase			Patient self-administered dose increase			Patient self-administered dose increase			Patient self-administered dose increase							
Eligibility Criteria		X																					
Medical History	X																						
Physical & Neurological Exam	X	X		X				X										X		X		X	
Blood genotyping (GBA&LRRK2)**	X																						
Routine bloods**[b]	X	X						X										X		X		X	
Blood enzyme activity & ELISA**	X							X										X		X		X	
Blood ambroxol**	X							X										X		X			
CSF Enzyme Activity & ELISA**		X																		X		X[m]	
CSF Ambroxol**		X																		X		X[m]	
Vital Signs	X	X		X[d*]				X										X		X		X	
Weight (& height at screening)[e]	X	X		X				X										X		X		X	
ECG	X	X		X[d]																X		X	
Pregnancy test**[a]	X	X		X[c]				X[c]										X[c]		X[c]		X	
Lumbar Puncture[e]		X																		X		X[m]	
Urine Collection **[e]				X[c]				X[c]										X		X		X	
Hoehn & Yahr Scale	X																						
MDS-UPDRS[e]		X		X[c]														X[c]		X[c]		X	
Cognitive Scales: MoCA, NMSS, NMS[c,e]				X																X			
IMP Compliance					X	X		X	X		X	X		X	X		X	X	X	X			
Dosing Instructions/Reminder[f]				X[k]		X		X[k]	X			X			X			X[k]		X[k]			
Dispensing				X														X					
Collect IMP Packaging & Pill Count								X[l]										X		X		X[n]	
Adverse Events	X	X	X	X	X	X		X	X		X	X		X	X		X	X	X	X	X	X	X
Concomitant Medication	X	X	X	X	X	X		X	X		X	X		X	X		X	X	X	X	X	X	X

*Optional LP
**See lab manual
[a]Pregnancy test (serum, FSH, oestradiol and hGC) at screening and urine (hGC) dipstick at subsequent visits for women of childbearing potential.
[b]Routine blood panel: Haematology, biochemistry, coagulation, uric acid.
[c]Conducted pre-dose
[d]Conducted pre-dose and 1 hour post IMP administration
[d*]Conducted pre-dose, 1 hour post IMP administration, and prior to discharge
[e]Morning assessment
[f]Dosing instructions; Participants should be instructed the following: dose 1 (day 1-7, 60 mg TID), dose 2 (day 8-14, 120 mg TID), dose 3 (day 15-21, 180 mg TID), dose 4 (day 22-28, 300 mg TID), and dose 5 (day 29-186, 420 mg TID). Each dose may be taken with or without a meal and with water and be administered in the morning, afternoon and evening each day as instructed by the Investigator.
[f*]On visit 3, day 1 participants can be discharged and instructed to take the remaining daily dose(s) at home as soon as all the protocol procedures are completed.
[g]Escalation visit conducted at LWENC
[h]Escalation conducted at home by patient
[j]If day 279 is early termination visit participants are expected to undergo all assessments for this visit and may consent to the optional lumbar puncture.
[k]IMP administered in clinic; see also [f] and [f*].
[l]Pill count only, no packing collected.
[m]Only conducted if participant agrees to optional lumbar puncture at visit 7.
[n]Collect packaging (no pill count) if this is early termination visit.

Appendix 2 – UK Parkinson disease society brain bank clinical diagnostic criteria

UK PARKINSON'S DISEASE SOCIETY BRAIN BANK CLINICAL DIAGNOSTIC CRITERIA*

Step 1. Diagnosis of Parkinsonian Syndrome

- Bradykinesia
- At least one of the following
  - Muscular rigidity
  - 4-6 Hz rest tremor
  - postural instability not caused by primary visual, vestibular, cerebellar, or proprioceptive dysfunction

Step 2 Exclusion criteria for Parkinson's disease

- history of repeated strokes with stepwise progression of parkinsonian features
- history of repeated head injury
- history of definite encephalitis
- oculogyric crises
- neuroleptic treatment at onset of symptoms
- more than one affected relative
- sustained remission
- strictly unilateral features after 3 years
- supranuclear gaze palsy
- cerebellar signs
- early severe autonomic involvement
- early severe dementia with disturbances of memory, language, and praxis
- Babinski sign
- presence of cerebral tumor or communication hydrocephalus on imaging study
- negative response to large doses of levodopa in absence of malabsorption
- MPTP exposure

Step 3 supportive prospective positive criteria for Parkinson's disease
Three or more required for diagnosis of definite Parkinson's disease in combination with step one

- Unilateral onset
- Rest tremor present
- Progressive disorder
- Persistent asymmetry affecting side of onset most
- Excellent response (70-100%) to levodopa
- Severe levodopa-induced chorea
- Levodopa response for 5 years or more
- Clinical course of ten years or more

**From: Hughes AJ, Daniel SE, Kilford L, Lees AJ. Accuracy of clinical diagnosis of idiopathic Parkinson's disease. A clinico-pathological study of 100 cases. JNNP 1992;55:181-184.*
