# Supplementary material for: Ambroxol for the Treatment of Patients With Parkinson Disease With and Without Glucocerebrosidase Gene Mutations: A Nonrandomized, Noncontrolled Trial
Source: JAMA Neurol. 2020 Jan 13;77(4):427–34. doi: 10.1001/jamaneurol.2019.4611 (PMC6990847; doi:10.1001/jamaneurol.2019.4611)
Supplement: Supplement 2. — eMethods 1. Summary of Product Characteristics of Ambroxol eMethods 2. Inclusion and Exclusion Criteria eMethods 3. Details of Biochemical Assays eMethods 4. Optimization Assays for Liquid Chromatography Mass Spectrometry of Ambroxol eMethods 5. Statistical Analysis Plan eFigure 1. Change in CSF Ambroxol Concentration Plotted Against Inverse of Normal eFigure 2. Change in CSF GCase Activity Plotted Against Inverse of Normal eFigure 3. Scatter Plot of Change in CSF GCase Activity Against Change in CSF Ambroxol eFigure 4. Influence of Change in PD Meds on MDS-UPDRS Total Score eFigure 5. Influence of Change in PD Meds on MDS-UPDRS Part 3 Score eTable 1. Summary of Study Visits eTable 2. Summary Table of Biochemical Results Including GBA1 and Non-GBA1 Subgroups eTable 3. Summary Table of Clinical Results Including GBA1 and Non-GBA1 Subgroups eTable 4. Summary Table of CSF Biochemical Results Including GBA1 and Non-GBA1 Subgroups in Those Who Underwent 3 Lumbar Punctures eTable 5. Summary Table of Adverse Events Felt to Be Possibly, Probably, or Definitely Related to Ambroxol eTable 6. List of Recorded Adverse Events [file jamaneurol-77-427-s002.pdf]

Supplementary Online Content

Mullin S, Smith L, Lee K, et al. Ambroxol for the treatment of patients with Parkinson disease with and without glucocerebrosidase gene mutations: a nonrandomized controlled trial. *JAMA Neurol*. Published January 13, 2020. doi:10.1001/jamaneurol.2019.4611

eMethods 1. Summary of Product Characteristics of Ambroxol

eMethods 2. Inclusion and Exclusion Criteria

eMethods 3. Details of Biochemical Assays

eMethods 4. Optimization Assays for Liquid Chromatography Mass Spectrometry of Ambroxol

eMethods 5. Statistical Analysis Plan

eFigure 1. Change in CSF Ambroxol Concentration Plotted Against Inverse of Normal

eFigure 2. Change in CSF GCase Activity Plotted Against Inverse of Normal

eFigure 3. Scatter Plot of Change in CSF GCase Activity Against Change in CSF Ambroxol

eFigure 4. Influence of Change in PD Meds on MDS-UPDRS Total Score

eFigure 5. Influence of Change in PD Meds on MDS-UPDRS Part 3 Score

eTable 1. Summary of Study Visits

eTable 2. Summary Table of Biochemical Results Including *GBA1* and Non-*GBA1* Subgroups

eTable 3. Summary Table of Clinical Results Including *GBA1* and Non-*GBA1* Subgroups

eTable 4. Summary Table of CSF Biochemical Results Including *GBA1* and Non-*GBA1* Subgroups in Those Who Underwent 3 Lumbar Punctures

eTable 5. Summary Table of Adverse Events Felt to Be Possibly, Probably, or Definitely Related to Ambroxol

eTable 6. List of Recorded Adverse Events

This supplementary material has been provided by the authors to give readers additional information about their work.

© 2020 Mullin S et al. *JAMA Neurology*.

eMethods 1. Summary of Product Characteristics of Ambroxol

SUMMARY OF PRODUCT CHARACTERISTICS

1. NAME OF THE MEDICINAL PRODUCT

Ambrosan 60 mg tablets

2. QUALITATIVE AND QUANTITATIVE COMPOSITION

Each tablet contains 60 mg of ambroxol hydrochloride. Excipients with known effect: 109 mg of lactose monohydrate. For the full list of excipients, see section 6.1.

3. PHARMACEUTICAL FORM

Tablet
Almost white round cross-scored tablets, diameter 9.5 mm. The tablet can be divided into 4 equal doses.

4. CLINICAL PARTICULARS

4.1 Therapeutic indications

Mucolytic therapy in acute and chronic bronchopulmonary diseases associated with abnormal mucus secretion and impaired mucus transport (inflammations of upper and lower respiratory airways, infection diseases of respiratory airways), inflammatory rhinopharyngeal diseases.
The product can be used in children from 5 years, adolescents and adults.

4.2 Posology and method of administration

Adults and adolescents over 12 years: 1 tablet 2 times a day. This dosing regimen is suitable for the treatment of acute respiratory disease and initial treatment of chronic conditions for up to 14 days. Children 5–12 years: 1⁄4 of tablet 2–3 times a day. The tablets should be taken after meal and rinsed down with sufficient amount of liquid. Liquid consumption enhances the mucolytic effect of ambroxol. Treatment duration with Ambrosan 60 mg tablets is given individually depending on the concrete indication and type of disease.

4.3 Contraindications

Hypersensitivity to the active substance or to any of the excipients listed in section 6.1.

4.4 Special warnings and precautions for use

In the presence of impaired renal function or severe hepathopathy ambroxol should be administered with precaution. Accumulation of the metabolites of ambroxol generated in the liver can be expected in the presence of severe renal insufficiency.
There have been very few reports of severe skin lesions such as Stevens-Johnson syndrome and Lyell’s syndrome in temporal association with the administration of expectorants such as ambroxol. Mostly these could be explained by the severity of the patient’s underlying disease and/or concomitant medication. In addition during the early phase of a Stevens-Johnson syndrome or Lyell’s syndrome a patient can first experience non-specific influenza-like prodromes like e.g. fever, aching body, rhinitis,

© 2020 Mullin S et al. *American Medical Association.* All rights reserved.

cough and sore throat. Misled by these non-specific influenza-like prodromes it is possible that a symptomatic treatment is started with a cough and cold medication. Therefore, if new skin or mucosal lesions occur, medical advice should be sought immediately and treatment with ambroxol discontinued as a precaution.

This medicinal product contains lactose. Patients with rare hereditary problems of galactose intolerance, the Lapp lactase deficiency or glucose-galactose malabsorption should not take this medicine.

4.5 Interaction with other medicinal products and other forms of interaction

Concomitant administration of ambroxol and antibiotics (amoxicillin, cefuroxime, erythromycin) results in increased antibiotic concentrations in bronchopulmonary secretion and sputum. This effect could be used therapeutically. No clinically relevant unfavourable interaction with other medications has been reported.

4.6 Pregnancy and lactation

Ambroxol crosses the placental barrier. Animal studies do not indicate direct or indirect harmful effects with respect to pregnancy, embryonal/foetal development, parturition or postnatal development. Extensive clinical experience after the 28th week of pregnancy has shown no evidence of harmful effects on the foetus. Nonetheless, the usual precautions regarding the use of drugs during pregnancy should be observed. Especially during the first trimester, the use of medicines containing ambroxol is not recommended. Ambroxol is excreted in breast milk. Although unfavourable effects on breastfed infants would not be expected, Ambrosan 60 mg tablets is not recommended for use in nursing mothers.

4.7 Effects on ability to drive and use machines

There is no evidence for an effect on the ability to drive and use machines. Studies on the effects on the ability to drive and use machines have not been performed.

4.8 Undesirable effects

Frequency of undesirable effects is defined using the following convention: Very common (≥1/10)
Common (≥1/100 to <1/10)
Uncommon (≥1/1,000 to <1/100)
Rare (≥1/10,000 to <1/1,000)
Very rare (<1/10,000)
Not known (cannot be estimated from the available data)

Ambrosan 60 mg tablets is usually well tolerated. During treatment, it can occur:

Immune system disorders, Skin and subcutaneous tissue disorders:
Rare: Rash, urticaria.
Not known: Anaphylactic reactions including anaphylactic shock, angioedema, pruritus and other hypersensitivity.

© 2020 Mullin S et al. *American Medical Association*. All rights reserved.

Gastrointestinal disorders:

Common: Nausea.

Uncommon: Dyspepsia, vomiting, diarrhoea and abdominal pain.

4.9 Overdose

No specific overdose symptoms have been reported in man to date. Based on accidental overdose and/or medication error reports the observed symptoms are consistent with the known side effects of ambroxol at recommended doses and may need symptomatic treatment.

5. PHARMACOLOGICAL PROPERTIES

5.1 Pharmacodynamic properties

Pharmacotherapeutic group: expectorants, mucolytics. ATC code: R05CB06.

Preclinically ambroxol, the active substance of Ambrosan 60 mg tablets, has been shown to increase secretion of mucus in respiratory system and modify its viscosity. It enhances pulmonary surfactant production and stimulates ciliary activity. These actions result in improved mucus flow and transport (mucociliary clearance). Enhancement of mucous secretion and mucociliary clearance facilitates expectoration and eases cough. Ambroxol decreases bronchial hyperreactivity, increases secretion of IgA in bronchial mucus and shows antioxidant activity.

5.2 Pharmacokinetic properties

Absorption of ambroxol after oral use is rapid and nearly complete, with dose linearity in the therapeutic range. Maximum plasma levels are reached within 0.5 to 3 hours. In the therapeutic range plasma protein binding is approximately 90%. Free fraction of ambroxol is distributed from blood to tissues relatively well, with the highest concentration found in the lungs. Plasma half-life is 7 to 12 hours, accumulation has not been shown. About 30% of the administered oral dose is eliminated via first pass. Ambroxol is metabolised primarily in the liver by conjugation. About 90% of the dose is eliminated by kidneys.

5.3 Preclinical safety data

Ambroxol has a low index for acute toxicity. In repeat-dose studies, oral doses of 150 mg/kg/day (mouse, 4 weeks), 50 mg/kg/day (rat, 52 and 78 weeks), 40 mg/kg/day (rabbit, 26 weeks) and 10 mg/kg/day (dog, 52 weeks) were the no-observed adverse effect level (NOAEL). No toxicological target organs were detected. Four week intravenous toxicity studies with ambroxol in rats (4, 16 and 64 mg/kg/day) and in dogs (45, 90 and 120 mg/kg/day (infusion 3 h/day)) showed no severe local and systemic toxicity including histopathology. All adverse effects were reversible.

Ambroxol was neither embryotoxic nor teratogenic when tested at oral doses up to 3000 mg/kg/day in rats and up to 200 mg/kg/day in rabbits. The fertility of male and female rats was not affected up to 500 mg/kg/day. The NOAEL in the peri- and post-natal development study was 50 mg/kg/day. At 500 mg/kg/day, ambroxol was slightly toxic for dams and pups, as shown by a retarded body-

© 2020 Mullin S et al. *American Medical Association.* All rights reserved.

weight development and reduced litter size.

Genotoxicity studies *in vitro* (Ames and chromosome aberration test) and *in vivo* (mouse micronucleus test) did not reveal any mutagenic potential of ambroxol.

Ambroxol did not show any tumorigenic potential in carcinogenicity studies in mice (50, 200 and 800 mg/kg/day) and rats (65, 250 and 1000 mg/kg/day) when treated with a dietary admixture for 105 and 116 weeks, respectively.

6. PHARMACEUTICAL PARTICULARS

6.1 List of excipients

Lactose monohydrate, granulated microcrystalline cellulose, copovidone, magnesium stearate.

6.2 Incompatibilities

Not applicable.

6.3 Shelf life

4 years

6.4 Special precautions for storage

This medicinal product does not require any special storage conditions.

6.5 Nature and contents of container

Transparent PVC/PVdC/Al blister, carton. Pack size: 20, 30, 60, 100 or 500 tablets. Not all pack sizes may be marketed.

6.6 Special precautions for disposal and other handling

Any unused medicinal product or waste material should be disposed of in accordance with local requirements.

7. MARKETING AUTHORISATION HOLDER

PRO.MED.CS Praha a.s., Telčská 1, 140 00 Praha 4, Czech Republic

8. MARKETING AUTHORISATION NUMBER(S)

52/093/13-C

© 2020 Mullin S et al. *American Medical Association.* All rights reserved.

eMethods 2. Inclusion and Exclusion Criteria

1. Male or female;
2. Age $\geq$ 40 and $\leq$ 80 years of age;
3. Confirmed diagnosis of Parkinson disease at any time; and Hoehn and Yahr criteria, confirmed staged between I – III, inclusive;
4. Able and willing to provide informed consent prior to any study related assessments and procedures at screening visit 1;
5. Capable of complying with all study procedures, including fasting lumbar puncture;
6. Willing to provide a blood sample for screening genomic for Parkinson Disease related DNA analysis and/or consent to Investigators obtaining and using participants previous DNA results if applicable;
7. Willing and able to self-administer oral ambroxol medication, from day 1 to 186 (at 60 mg TID (day 1-7), 120 mg TID (day 8-14), 180 mg TID (day 15-21), 300 mg TID (day 22-28) and 420 mg TID (day 29-186));
8. Able to travel to the participating study site;
9. A female participant is eligible to participate if she is of:

- Non-childbearing potential defined as pre-menopausal females with a documented tubal ligation or hysterectomy; or postmenopausal defined as 12 consecutive months of spontaneous amenorrhea, at least 6 weeks post-surgical bilateral oophorectomy (with or without hysterectomy) or post tubal ligation. In questionable cases, menopausal status will be confirmed by demonstrating levels of follicle stimulating hormone (FSH) 25.8 – 134.8 IU/L and oestradiol < 201 pmol/l at entry.
- Women of child-bearing potential must use accepted contraceptive methods (listedat subsequent visits if applicable. An additional pregnancy test will be performed, and results obtained, prior to administration of the first dose of ambroxol.

Accepted contraception methods:

• True abstinence: When this is in line with the preferred and usual lifestyle of the participant. [Periodic abstinence (e.g., calendar, ovulation, symptothermal, post-ovulation methods) and withdrawal are not acceptable methods of contraception).

© 2020 Mullin S et al. *American Medical Association*. All rights reserved.

Contraceptive Methods with a Failure Rate of < 1%:

- Oral contraceptive, either combined or progestogen alone;
- Injectable progestogen;
- Implants of levonorgestrel;
- Estrogenic vaginal ring;
- Percutaneous contraceptive patches;
- Intrauterine device (IUD) or intrauterine system (IUS) that meets the <1% failure rate as stated in the product label;

Please note:

- All male and female participants of child bearing potential must agree with their partners to use double-barrier birth control or abstinence while participating in the study and for 2 weeks following the last dose of the study drug.

- Participants may continue to take PD medications including glutamate antagonists, anticholinergics, dopamine agonists, Levodopa (L-DOPA and decarboxylase (DDC) inhibitor), Monoamine oxidase B (MAO-B) inhibitors catechol-O-methyltransferase (COMT) inhibitors, beta blockers, selective serotonin uptake inhibitors (SSRIS), tricyclic antidepressants (TCAs) and indomethacin.

Exclusion Criteria:

Participants are excluded from participating in this study if 1 or more of the following criteria are met:

1. Current treatment with anticoagulants (e.g. warfarin) that might preclude safe completion of the lumbar puncture and in the opinion of the Investigator;
2. Current use of investigational medicinal product or participation in another interventional clinical trial or who have done so within 30 days prior to the first dose in the current study;
3. Exposure to more than three investigational medicinal products within 12 months prior to the first dose in the current study;
4. Confirmed dysphagia that would preclude self-administration of ambroxol up to 7

© 2020 Mullin S et al. *American Medical Association*. All rights reserved.

tablets TID for the duration of day 1 to day 186);

5. Significant known lower spinal malformations or other spinal abnormalities that would preclude lumbar puncture;
6. History of known sensitivity to the study medication,ambroxol or its excipients (lactose monohydrate, granulated microcrystalline cellulose, copovidone and magnesium stearate) in the opinion of the investigator that contraindicates their participation;
7. History of known rare hereditary disorders of galactose intolerance, Lapp lactase deficiency or glucose-galactose malabsorption;
8. Evidence or history of hypersensitivity to lidocaine or its derivatives;
9. History of drug abuse or alcoholism in the opinion of the Investigator that would preclude participation in the study;
10. Donation of blood (one unit or 350 ml) within three months prior to receiving the first dose of the study drug;
11. Pregnant or breastfeeding;
12. All participants of child bearing potential in the opinion of the Investigator that would preclude participation in the study and who do not agree to use double-barrier birth control or abstinence while participating in the study and for two weeks following the last dose of study drug;
13. Any clinically significant or unstable medical or surgical condition that in the opinion of the PI or PI-delegated clinician may put the participant at risk when participating in the study or may influence the results of the study or affect the participant’s ability to take part in the study, as determined by medical history, physical examinations, electrocardiogram (ECG), or laboratory tests. Such conditions may include:
    1. Impaired renal function
    2. Moderate/Severe hepatic impairment
    3. A major cardiovascular event (e.g. myocardial infarction, acute coronary syndrome, decompensated congestive heart failure, pulmonary embolism, coronary revascularisation that occurred within 6 months prior to the screening visit.

© 2020 Mullin S et al. *American Medical Association*. All rights reserved.

eMethods 3. Details of Biochemical Assays

CSF GCase activity

Cerebrospinal fluid was collected in 15ml polypropylene tubes (Starstedt 62.554.002) centrifuged at 2200G for 10min and frozen at -80 degrees within an hour of collection in 200ul aliquots in polypropylene 2ml microtubes (Starstedt 72.694.217). CSF was vortexed and centrifuged at 8000rpm for 10 minutes. Ten repeats of 20ul of CSF were added to a 96 well plate. The reaction was commenced by adding 40ul of 5uM 4-MU-β-D-glucopyranoside solution dissolved in McIlvaine citrate buffer (0.15M, pH 5.9) with 28nM sodium taurochlorate. The plate was covered and incubated at 38 degrees for 3 hours whereupon the reaction was stopped using 240ul glycine stopping buffer (1M, pH 10.4). A standard was prepared by 5 repeats of 200ul 1mM 4-methylumbelliferone in distilled water and adding 100ul glycine buffer. The plate was measured at excitation wavelength of 365nm, emission of 450nm with a PerkinElmer (Waltham, MA) fluorescence spectrometer. GCase assays were expressed as nanomoles of substrate catalysed per millilitre of CSF per hour using the standard and a blank in which the CSF was replaced distilled water. Our optimisation experiments have indicated an *inter* and *intra* assay coefficient of variance of 3% and 2% respectively

Leucocyte pellet GCase activity assay

Leucocyte pellets were collected in BD Vacutainer® CPT™ Mononuclear Cell Preparation Tube – Sodium Heparin (catalogue number 362753). Tubes were spun at 3000rpm for 25 minutes. Leucocytes were collected from above the intermediate density liquid and aliquoted into 1.5ml microtubes (Starlabs cat. E1415-2230). Samples were spun at 6000rpm for 6 minutes. If no pellet was visible at this point a further spin at 6000rpm for 6 minutes was carried out. The supernatant was drained and the sample was re-suspended in 1ml red cell lysis buffer (155 mM NH_4Cl 12 mM $NaHCO_3$ 0.1 mM EDTA) for 10 minutes at room temperature. The sample was spun again, the supernatant drained and re-suspended in PBS. One final spin was carried out and the supernatant was drained prior to being assayed or frozen at -80 degrees. In the case of the latter this was always within 1 hour of collection.

Leucocyte pellets were lysed in Fermantas ProteoJET Mammalian Cell Lysis Reagent and diluted 8X in McIlvaine citrate buffer (0.15M, pH 5.9). The reaction was commenced by adding 40ul of 5uM 4-MU-β-D-glucopyranoside solution dissolved in McIlvaine citrate buffer (0.15M, pH 5.9). The plate was covered and incubated at 38 degrees for 3 hours

© 2020 Mullin S et al. *American Medical Association*. All rights reserved.

whereupon the reaction was stopped using 240ul glycine stopping buffer (1M, pH 10.4). A standard was prepared by preparing 5 repeats of 200ul 1mM 4-methylumbelliferone in distilled water and adding 100ul glycine buffer. The plate was measured at excitation of 365nm, emission of 450nm with a PerkinElmer (Waltham, MA) fluorescence spectrometer. Protein concentration was determined using a Pierce BCA Protein Assay. GCase assays were expressed as nanomoles of substrate catalysed per milligram of protein per hour.

Liquid chromatography mass spectrometry of GCase and glucosylceramide

To 600 µL CSF, 3 pmol heavy labelled GCase peptide, NFVDSPII**V**DITK (Genscript, USA), were added as internal standard. The samples were freeze dried and trypsin digested as previously described [1]. Sample clean-up was performed using C_{18} cartridges (Biotage, Sweden) which were washed with two 1 mL aliquots of 70% acetonitrile, 0.1% trifluoroacetic acid (TFA) and primed with two 1 mL aliquots of 0.1% TFA before the sample was loaded. The flow-through was re-applied and the bound peptides washed with one 1 mL aliquot of 0.1% TFA. The peptides were eluted with 500 µL 70% acetonitrile, 0.1% TFA and solvents were evaporated using a SpeedVac. Before analysis, the peptides were re- constituted in 120 µL 3% acetonitrile, 0.1% TFA.

Mass spectral analysis was performed as previously described [2]. 5 µL of digest were injected and peptides separated on a Waters Acquity UPLC system coupled to a Xevo TQ-S mass spectrometer. The monitored peptides were quantifier NFVDSPIIVDITK (m/z 730.9>1100.6), qualifier SYFSEEGIGYNIIR (m/z 824.7>905.5) and internal standard (m/z 733.9>1106.6). The data were integrated using an in-house script written in Python. Analyte responses were normalised to internal standard response before concentrations were calculated in the average of three technical replicates using a calibration curve constructed from synthetic peptides (GenScript, USA) ranging from 0 to 1 pmol μL^{-1}. Quality control samples composed of pooled digest were run at least every seventh sample and showed a coefficient of variance of 5.6%.

Glucosylceramides were extracted using a modified Bligh and Dyer procedure. Briefly, to 200 µL CSF, 400 µL methanol containing the deuterated internal standard glucosylceramide C16:0-D_3 (#1533, Matreya, USA) were added. Samples were vortexed and stored on dry ice followed by sonication. 200 µL chloroform were added and the samples were again vortexed and stored on dry ice followed by sonication. Finally, 200 µL water and 200 µL chloroform

© 2020 Mullin S et al. *American Medical Association*. All rights reserved.

were added. The samples were incubated in room temperature for 60 minutes before centrifugation at +4 °C, 5000*g* for 10 minutes. 300 μL of the organic phase were transferred to a glass vial and solvents were evaporated under nitrogen. Before analysis, the samples were re-constituted in 50 μL methanol.

Analysis was performed using a Waters Acquity Liquid Chromatography Quaternary Solvent Manager system coupled to a Waters Xevo TQ-S mass spectrometer. 5 μL of sample were injected and separated on a Waters Acquity UPLC BEH C8 column, 1.7 μm, 2.1 x 50 mm with a VanGuard pre-column of the same chemistry. The mobile phase consisted of A: water, 0.1% formic acid and B: methanol, 0.1% formic acid. The gradient profile lasted for 6.5 minutes and was initially set to 50% B for 0.2 minutes, then linearly increased to 100% B over 1.8 minutes. The column was washed with 100% B for 1 minute, then returning to equilibrate at initial conditions before the next injection. The flow rate was 0.5 mL min^{-1}. Glucosylceramides were detected using multiple reaction monitoring in positive mode, see below for transitions. The data were integrated using an in-house script written in Python. Analyte responses were normalised to internal standard response before concentrations were calculated in the average of three technical replicates using a calibration curve created from glucosylceramide standard (#1522, Matreya, USA) ranging from 0 to 0.5 ng $μL^{-1}$. Quality control samples consisting of pooled extract were run at least every seventh sample and showed a coefficient of variance of 11%.

ISOFORMS	MONITORED TRANSITIONS
C16:0-d3 glucosylceramide (internal standard)	725.7 >
545.7/536.7 C16:1 glucosylceramide	720.7 > 558.7
C16:0 glucosylceramide	722.7 > 542.6/560.6
C18:1 glucosylceramide	748.6 > 586.6
C18:0 glucosylceramide	750.6 > 588.6
C20:1 glucosylceramide	776.6 > 614.6
C20:0 glucosylceramide	778.6 > 616.7
C22:1 glucosylceramide	804.7 > 642.7
C22:0 glucosylceramide	806.7 > 644.7
C22:0-OH glucosylceramide	820.7 > 658.8
C24:2 glucosylceramide	830.7 > 668.7

© 2020 Mullin S et al. *American Medical Association*. All rights reserved.

C24:1 glucosylceramide	832.7 > 670.7
C24:0 glucosylceramide	834.7 > 672.7
C24:2-OH glucosylceramide	846.7 > 684.8
C24:1-OH glucosylceramide	848.7 > 686.8
C24:0-OH glucosylceramide	850.7 > 688.8
C26:1 glucosylceramide	860.7 > 698.8
C26:0 glucosylceramide	862.7 > 700.8
C26:1-OH glucosylceramide	876.7 > 714.8
C26:0-OH glucosylceramide	878.7 > 716.8

Liquid chromatography mass spectrometry of ambroxol

Liquid chromatography mass spectrometry quantification of ambroxol was performed by Laboratories of the Government Chemist (LGC), Teddington, UK. The achieved limit of detection/quantification was 0.5ng/ml and 1.0ng/ml respectively, the latter being in excess of specified limit of detection (included in the tender and protocol) of 20 ng/ml. Details of the optimisation assays are provided in supplementary materials section 8.

Alpha synuclein and tau measurement

Total A-SYN concentration was measured using a commercially available enzyme-linked immunosorbent assay (ELISA) from Covance (Covance, Dedham, MA, USA). Tau concentration was measured using a commercially available INNOTEST ELISA (Fujirebio, Ghent, Belgium).

In vitro assays estimating effect of ambroxol of CSF GCase activity

For each condition fluorescence was measured with five technical repeats (5 wells) per subject, per condition all measured and performed on a single 96 well plate. For each subject CSF was pooled from the same multiple aliquots to prevent variability due to variation between aliquots (i.e CSF was not pooled from different subjects). CSF GCase activity was measure on control CSF, CSF with 500nM ambroxol added and two negative controls: CSF denatured at 80 degrees and CSF with 1mM Conditurol B epoxide (CBE), an irreversible inhibitor of GCase enzyme, added to it.

Ambroxol powder (Sigma – A0363700) was dissolved in dimethyl sulfoxide (DMSO) to make a stock solution of 10mM. An intermediate stock of 100μM was made, aliquoted and

© 2020 Mullin S et al. *American Medical Association*. All rights reserved.

stored at -20°C. For the assay ambroxol was added in a 1:200 dilution directly to CSF to obtain a working concentration of 500nM and incubated at 37°C for 15min (to ensure the solution was dissolved) before adding to the plate.

A 10mM stock solution of Conditurol B epoxide (CBE), an irreversible inhibitor of GCase enzyme, was made by dissolving the solute in dH_2O and was subsequently aliquoted and stored at -20°C. For the assay, the CBE was added in a 1:10 dilution directly to CSF to obtain a working concentration of 1mM and incubated at 37°C for 15min before adding to the plate. As a second negative control, some of the CSF sample was denatured prior to performing the assay. Denaturation was achieved by heating the CSF to 80°C for a minimum of 15min in a dry bath incubator.

1. Heywood W, Wang D, Madgett TE, et al. The development of a peptide SRM-based tandem mass spectrometry assay for prenatal screening of Down syndrome. *J Proteomics*. 2012;75(11):3248-3257. doi:10.1016/j.jprot.2012.03.037.

2. Heywood WE, Galimberti D, Bliss E, et al. Identification of novel CSF biomarkers for neurodegeneration and their validation by a high-throughput multiplexed targeted proteomic assay. *Mol Neurodegener*. 2015;10:64. doi:10.1186/s13024-015-0059-y.

© 2020 Mullin S et al. *American Medical Association*. All rights reserved.

eMethods 4. Optimization Assays for Liquid Chromatography Mass Spectrometry of Ambroxol

Method – Sample preparation

- Protein precipitation method
    - 250ul matrix
    - Add 50ul Ambroxol standard solution (MeOH for blank)
    - Add 50ul Ambroxol-d5 internal standard
    - Vortex
    - Add 1ml acetonitrile for protein precipitation
    - Vortex
    - Centrifuge 20mins at 13000rpm
    - Transfer and dilute supernatant 50/50 in H2O
    - Inject

Method – LCMS

- LC method
    - Injection Volume: 10ul
    - Column: ACE Excel 2 C18-AR
    - Solvent A: H2O+ 0.1% Formic acid
    - Solvent B: MeCN+ 0.1% Formic Acid
    - Solvent C: MeCN+ 5% THF
    - Gradient:

Time	%A	%B	%C
0.00	85	15	0
1.00	85	15	0
10.00	15	85	0
10.01	0	0	100
12.00	0	0	100
12.01	85	15	0
16.00	85	15	0

- MS method
    - Transitions:
      379 => 264
      377 => 262
      281 => 266
    - IS Transitions:
      384 => 264
      382 => 262
    - Source parameters
      Temp: 450C
      CUR: 10
      GS1: 60
      GS2: 60
      IS: 5000v

- Valco valve
    - Flow directed to MS between 2 and 12 mins
    - Flow directed to waste at all other times

© 2020 Mullin S et al. *American Medical Association*. All rights reserved.

Validation

Consisting of at least 3 batches prepared on 3 separate days for each matrix, including:

- 9 point calibration in matrix 0.1-500ng/ml
- 6 replicate spikes at 2 concentrations (high and low)
- Triplicate injections at LOD and LOQ
- 2 pre-prepared QC's stored in -80 freezer

Acceptance criteria

- Retention time +/-0.1 mins
- Calibration R^2>0.995
- LOD: Peak-to-peak S/N >3, ratios of primary transitions within 20%
- LOQ: Peak-to-peak S/N >10, ratios of all transitions within 20%
- Recovery of spikes between 70-120%
- QC between 70-120%

Validation data -CSF

CSF: High spike - 100ng/ml

	Batch 1	Batch 2	Batch 3
High Spike1	99.2	99.2	105.4
High Spike2	96.5	91.4	100.6
High Spike3	99.2	90.6	102.6
High Spike4	98.3	94.0	98.9
High Spike5	96.5	93.2	104.3
High Spike6	98.3	98.3	97.8

Low spike - 1ng/ml

	Batch 1	Batch 2	Batch 3
Low Spike1	1.1	0.9	1.0
Low Spike2	1.1	0.9	1.1
Low Spike3	1.1	0.9	1.1
Low Spike4	1.0	0.7	1.0
Low Spike5	1.2	0.8	1.1
Low Spike6	1.1	0.8	1.0

CSF QC – 50ng/ml

QC1	61.2	69.2	49.2
QC2	59.8	66.4	48.9

© 2020 Mullin S et al. *American Medical Association*. All rights reserved.

Validation data - Serum

- Calculated concentrations must be between 70-120% of expected

Serum: High spike - 250ng/ml — Low spike - 5ng/ml

	Batch 1	Batch 2	Batch 3	Batch 4		Batch 1	Batch 2	Batch 3	Batch 4
High Spike1	239.6	252.3	256.3	241.5	Low Spike1	4.7	4.8	4.6	5.1
High Spike2	243.9	263.8	248.5	240.3	Low Spike2	4.5	4.9	4.6	4.7
High Spike3	256.1	268.2	233.7	239.3	Low Spike3	4.7	4.7	4.6	4.8
High Spike4	248.2	262.0	260.6	243.3	Low Spike4	4.5	5.1	4.7	5.2
High Spike5	249.1	265.6	254.5	237.9	Low Spike5	4.8	5.1	4.5	5.1
High Spike6	250.8	267.3	246.7	234.8	Low Spike6	4.7	5.4	4.7	5.3
1500 Spike	115.8	121.4	155.0	138.5	1500ng/ml spike diluted 1 in 10 post extraction				

Serum QC – 50ng/ml

QC1	53.8	63.5	65.0	47.2
QC2	53.3	64.5	63.8	46.4

Validation data

All acceptance criteria was met for all batches apart from the QC results. QC's were prepared by bulk spiking and then freezing aliquots at -80C. Due to instrument access the validation batches were completed over a longer time frame then expected, because of this we suspected the standards had degraded causing higher results for the frozen QC's.

For the final batch in each matrix a new stock and standards were prepared resulting in the QC now coming out at the correct level. All previously extracted QC's that were used in the validation were analysed again against the new calibration set.

© 2020 Mullin S et al. *American Medical Association*. All rights reserved.

Validation data - QC

- All QC's used during validation were re-run against a newly prepared calibration showing that all the QC's meet acceptance criteria.

Serum		CSF	
QC1	49.7	QC1	54.1
QC2	48.2	QC2	53.7
QC3	52.8	QC3	52.3
QC4	43.8	QC4	52.8
QC5	42.6	QC5	52.1
QC6	43.5	QC6	51.8
QC7	52.9		
QC8	56.4		

- Due to the time delay between the batches of patient samples, a new stock and standards would be prepared fresh before each analysis. This would eliminate the bias observed during the validation runs.

Summary

- This method is now validated for both serum and CSF

- A labelled internal standard is now included in the method, which will account for patient variation and improve the overall robustness of the method.

- LOD for both serum and CSF is 0.5ng/ml
- LOQ for both serum and CSF is 1ng/ml

© 2020 Mullin S et al. *American Medical Association*. All rights reserved.

eMethods 5. Statistical Analysis Plan

STATISTICAL ANALYSIS PLAN

Ambroxol in Disease Modificationin Parkinson Disease (AiM-PD Study)

A Phase IIA Prospective, Single-Centre, Open Label Clinical Trial to Evaluate the Safety, Tolerability and Pharmacodynamic Effects of Ambroxol in Patients with Parkinson Disease

VERSIO
N 1.0

Chief Investigator Professor Anthony

Schapira Sponsor University College London

(UCL)

Sponsor code 15/0118

Funder Cure Parkinson's Trust

EudraCT Number 2015-002571-24

IRAS ID

Amendment history:

Amendment number & brief description of amendment		Amendment date	Resultant version

© 2020 Mullin S et al. *American Medical Association*. All rights reserved.

CONTENTS

1. SIGNATURE PAGE 3
2. INTRODUCTION 4
3. BACKGROUND 4
4. research QUESTIONSAND HYPOTHESIS 4
5. DESCRIPTION OF VARIABLES 4
   5.1. Primary outcome....................4
   5.2. Secondary outcomes 4
   5.3. Baseline characteristics....................5
6. FOLLOW-UP ANDPARTICIPANT FLOW....................5
7. DATA PRESENTATION....................5
8. STATISTICAL ANALYSIS 6
   8.1. Descriptive analysis....................6
   8.2. Primary analysis 6
9. ADVERSE EVENTS....................7

MODEL CHECKING AND VALIDATION....................7

© 2020 Mullin S et al. *American Medical Association*. All rights reserved.

INTRODUCTION

This statistical analysis plan describes the analysis and output to be generated for AiM-PD Phase 2a trial. This plan is based on study protocol version 2.0 dated 15th July 2016

1. BACKGROUND

The overarching objective of the AiM phase IIa study is to determine whether ambroxol administered in oral doses can cross the blood-brain barrier (BBB) in sufficient concentrations that may in a later phase III study facilitate the study of a hypothesised neuroprotective treatment against Parkinson's disease(PD).

Ambroxol is already approved the European Medicines Agency as an over-the-counter cough linctus, for which drug safety studies have demonstrated a low acute toxicity. Ambroxol has been confirmed to modulate glucocerebrosidase (GCase) enzyme activity in a number of transgenic models carrying the Gaucher disease(GBA) gene. There is evidence that GBA mutations are associated with reduced levels of GCase activity. A reciprocal relationship has been demonstrated between GCase activity levels and cystolic protein, alpha-synuclein, the main constituen of Lewy bodies, which are a pathological hallmark of PD.

2. RESEARCH QUESTIONS ANDHYPOTHESIS

The primary research question in this proof-of-concept and safety study is whether after escalation of the dose in a safety study, ambroxol crosses the BBB in sufficient amounts to register a significant increase in cerebrospinal fluid (CSF) concentrations of the drug. We predict a concomitant change in GCaseactivity. There is, however, no pre-existing evidence for what constitutes a clinically meaningful change in either ambroxol levels or GCase activity. Therefore interpretation will be primarily through the size of effect and the width of the confidence intervals

Descriptive summaries shall be provided for all other outcomes by each visit and by GBA mutation, andfor baseline variables. Descriptive summaries shall also bepresented for the pair of primary outcomes by GBAmutation.

3. DESCRIPTION OFVARIABLES

3.1. Primary outcomes

The results for two primary outcomes of interest shall be reported:

- Change in Ambroxol concentration in CSF from baseline to the six month visit
- Change in GCase enzyme activity in CSF from baseline to six month visit

© 2020 Mullin S et al. *American Medical Association*. All rights reserved.

3.2. Secondary outcomes

This excludes free text, qualitative data, and meta-data such as the date of assessment and assessor details.

For each visit, where collected:

- Weight
- Vital signs: heart rate, blood pressure, respiration rate, temperature
- Abnormal results reported for each part of body after physical examination
- Abnormal results reported for each test of the neurological examination
- ELISA antibody panel results from CSF and blood biomarker analyses
    - Alpha-synuclein tau
    - panel of lysosomalproteins
- CSF enzyme activitypanel
    - Glucosylceramide lipidlevels
    - Glucocerebrosidase proteinlevels
- Blood enzyme activitypanel
    - Leucocyte GCase activitylevel
    - Serum chitotriosidase
    - Blood ambroxolconcentration
- MDS-UPDRS scale
- Sum of the scores from the items in the NMS questionnaire
- Sum of the scores from the items in the NMSS scale
- Sum of the scores from the items in the MoCA assessment

3.3. Baseline characteristics

- Gender
- Age
- Height
- GBA genotype

4. FOLLOW-UP AND PARTICIPANT FLOW

Outcomes and fixed baseline patient characteristics will be reported from screening at visits 1 and 2.All outcome variables will be reported at subsequent treatment phase visits 3, 4 5,6 and at the termination visit 7. The endpoint for the primary outcome is reported at visit 6.

Patient numbers and progression, along with the counts and severity of adverse events shall be presented in a flow diagram for the key stages of the trial at the screening visits, the telephone visits following dose escalation, and the treatment-phase visits recording outcomes.

© 2020 Mullin S et al. *American Medical Association*. All rights reserved.

7. DATA PRESENTATION

Summary statistics for the primary, baseline and secondary outcomes will be presented as appropriate point estimates with their standard deviations and the number of observations, or as counts with percentages, where the data are categorical. Additionally, summary statistics of the primary outcomes will be presented by GBA genotype, but no inference shall be made about the significance of the difference between genotypes.

For descriptive purposes only, 95% confidence intervals will be presented for the laboratory results for the enzyme activity, biomarker and antibody panels by GBA genotype, and shall not be used to determine the success of the study.

8. STATISTICAL ANALYSIS

Following the data lock, the following analyses will be undertaken by the Trial Statistician, and the results acted upon in accordance with the decision rule outlined in this statistical analysis plan.

8.1. Overall strategy

The sample size for the study was selected by the Chief Investigator but there was no formal sample size calculation or statistical consideration. The main focus of all analyses will be on the interpretation of 95% confidence intervals. The results from formal significance testing will be presented as supplementary information only, as indication of the degree of uncertainty.

8.2. Descriptive analysis

A summary of baseline characteristics and the outcomes at a baseline and at subsequent treatment- phase visits for the whole cohort will be presented as point estimates with their standard deviations, or as counts and percentages where the data are categorical. Additionally the 95% confidence intervals for the antibody, biomarker and enzyme activity panels shall be reported. No formal significance testing will be conducted, apart from the primary analysis.

8.3. Primary analysis

Primary analysis will be based on interpretation of the confidence intervals, arbitrarily covering 95% of each distribution of the mean changes in CSF ambroxol concentration and GCase enzyme activity, modelled according to at-distribution of degrees of freedom based on the sample size. Since only an increase in ambroxol is of interest (or possible, if participants not taking ambroxol), then the lower 95% confidence bound will be presented for the change in ambroxol. A two-sided 95% confidence interval will be presented for GCase activity. The distribution of the changes from baseline to visit six in ambroxol concentration and GCaseenzyme activity shall be inspected graphically and transformed, if necessary, to fit a

© 2020 Mullin S et al. *American Medical Association*. All rights reserved.

Normal distribution before calculating the confidence intervals. The confidence intervals will then be interpreted relative to the transformed null value, which might not be zero, if an offset has been applied.

To account for the quantification limit of the instrument measuring ambroxol concentrations, if the mean concentration at visit six is below the1.0ng/ml limit then CSFambroxol will be deemed to have not changed, regardless of the change from baseline to visit six.

8.4. Further descriptive analysis

The individual response in GCase activity to potential changes in CSF ambroxol concentrations will be further explored through a bivariate plot of the changes in each. It is unknown whether there will be a systematic difference between patients in CSF ambroxol to observe any potential relationship, having administered the same ambroxol dose to each. However, the Pearson correlation coefficient will be presented along with its 95% confidence intervals having transformed both variables, if such an observed relationship is non-linear.

9. ADVERSE EVENTS

Safety data and adverse events will be listed descriptively and include details of the event.

10. MODEL CHECKING ANDVALIDATION

All analyses will be undertaken using STATA v14.2.

Before undergoing the primary analysis, decisions about whether to transform the primary outcome (e.g. natural log) will be made based on plots of its distribution, and appropriate statistics reported for skewness if necessary. Checks will be undertaken to assess the robustness of the primary analysis based on the normality of the residuals.

© 2020 Mullin S et al. *American Medical Association*. All rights reserved.

eFigure 1. Change in CSF Ambroxol Concentration Plotted Against Inverse of Normal

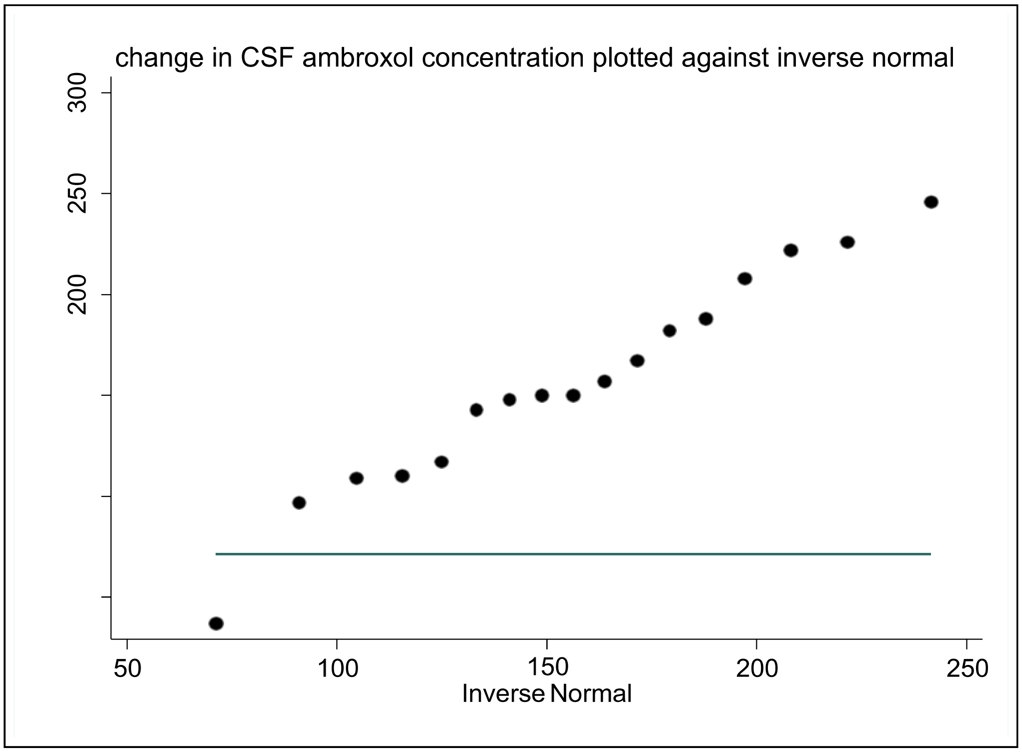

© 2020 Mullin S et al. *American Medical Association*. All rights reserved.

eFigure 2. Change in CSF GCase Activity Plotted Against Inverse of Normal

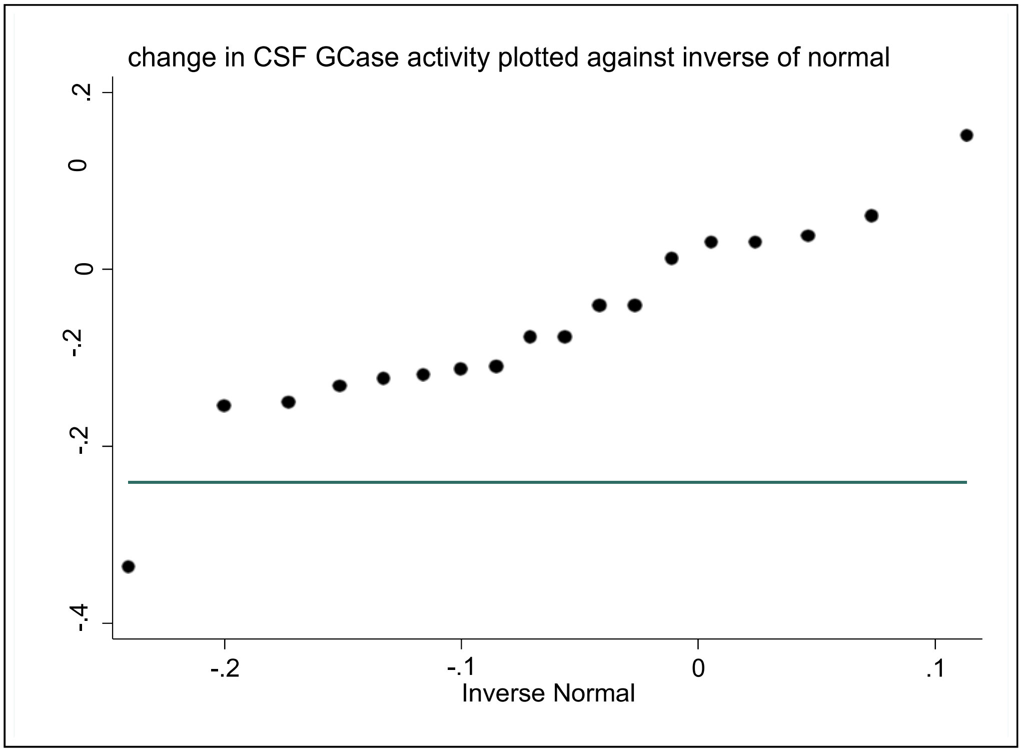

© 2020 Mullin S et al. *American Medical Association*. All rights reserved.

eFigure 3. Scatter Plot of Change in CSF GCase Activity Against Change in CSF Ambroxol

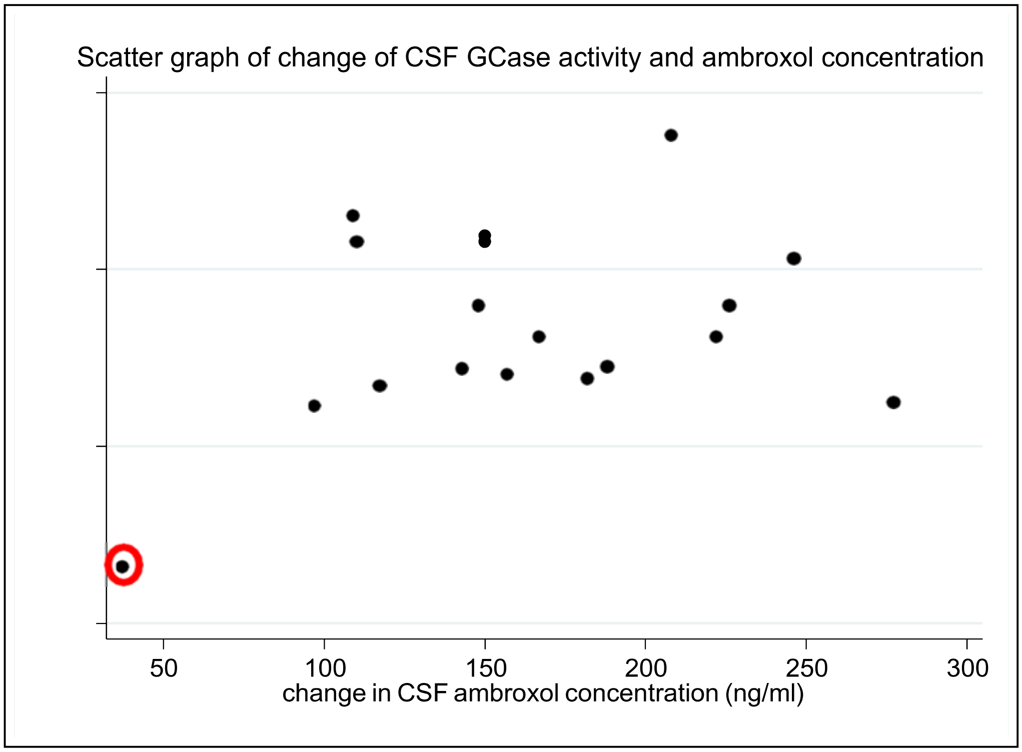

Dashed line represents no change in CSF GCase activity. We considered the distribution of samples for the primary outcomes. In the case of both CSF GCase and CSF ambroxol the data appeared to be normally distributed (see supplementary figures 1 and 2). We identified one outlier who had marked fall in CSF GCase activity with a minimal rise in CSF ambroxol concentration (circled in red in supplementary figure 3). We considered whether this could be due to blood contamination, however at both timepoints no red blood cells were seen on CSF microscopy. In the absence of a convincing scientific rationale to exclude the subject, they were included in both primary and secondary analyses.

© 2020 Mullin S et al. *American Medical Association*. All rights reserved.

eFigure 4. Influence of Change in PD Meds on MDS-UPDRS Total Score

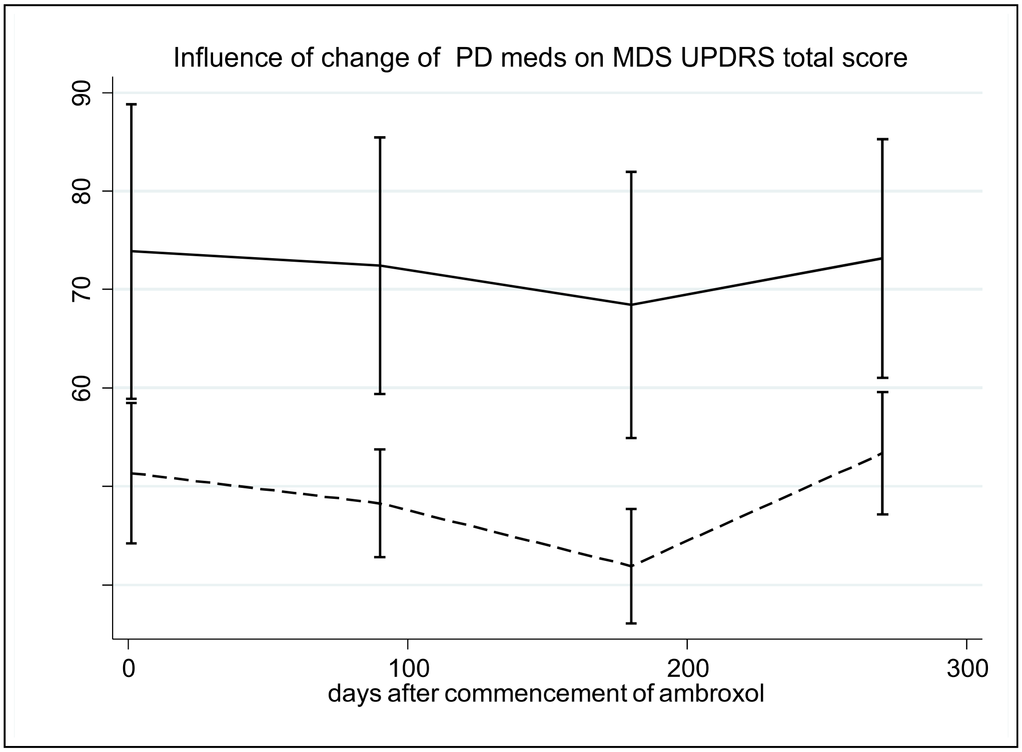

MDS-UPDRS total scores of those who did (solid) or did not (dashed) change PD meds during the study.

© 2020 Mullin S et al. *American Medical Association*. All rights reserved.

eFigure 5. Influence of Change in PD Meds on MDS-UPDRS Part 3 Score

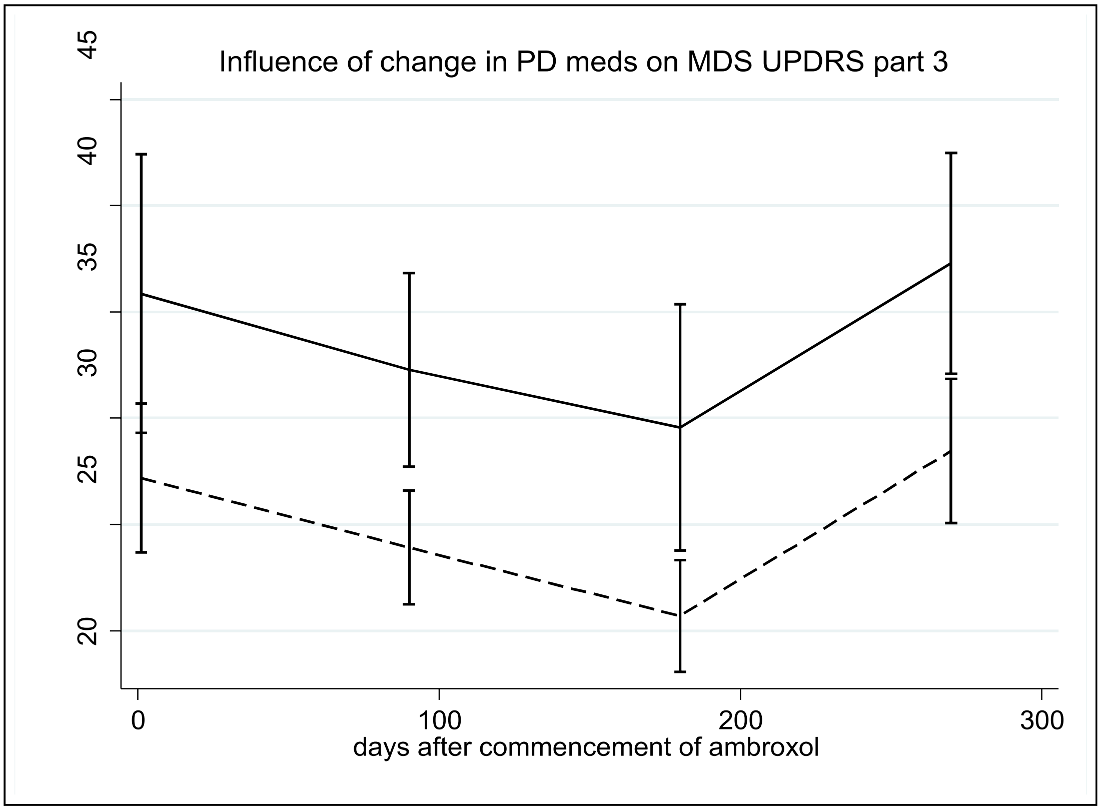

MDS-UPDRS part 3 scores of those who did (solid) or did not (dashed) change PD meds during study

© 2020 Mullin S et al. *American Medical Association.* All rights reserved.

eTable 1. Summary of Study Visits

**Summary of study visits**											
**Study Period**	**Screening Phase (Pre-Treatment Phase)**		**Treatment Phase**								**Follow Up Assessment and washout/Final visit (End of Study/Early Termination)**
Visit	**Visit 1 (at hospital)**	**Visit 2 [t] (at hospital)**	**Visit 3 (at hospital) Dose Escalation 1 [t]**	**Dose Escalation 2[t]**	**Visit 4 [t] (at hospital)**	**Dose Escalation 3[t]**	**Dose Escalation 4[t]**	**Dose Escalation 5[t]**	**Visit 5 (at hospital) Month 3**	**Visit 6 (at hospital) Month 6**	**Visit 7 (at hospital) Month 9**
**Dose Escalation Day**			**1**	**8**		**15**	**22**	**29**	**93**	**186**	**279**
**Visit Window**			(within 60 days of screening Visit 1 & 2)		*(within 3 days after dose escalation 2)*				(+/- 14 days)	(+/- 14 days)	**Early termination visit (+/- 30 days)
**Day**	-60	-60	1	8-14		15-21	22-28	29-186	93	186	279
Informed consent	X										
Medical History	X										
Physical and neurological examinations	X	X	X		X				X	X	X
MDS-UPDRS[c]		X	X[a]						X	X	X
Screening Inclusion/Exclusion criteria		X									
Screening Genotyping (*GBA* & *LRRK2*) if applicable	X										
Vital Signs (HR, BP RR & Temperature)	X	X	X[b,b*,d*]		X				X	X	X
Height and Weight[3,c]	X	X	X		X				X	X	X
ECG	X	X	X[b]							X	X
Lumbar Puncture[c] (Up to 20 mL)		X								X	X*
Pregnancy Test[1***] (if applicable)	X	X	X[a]		X				X	X	X
Routine blood collection/panel[2,***]	X	X			X				X	X	X
**Study Period**	**Screening Phase (Pre-Treatment Phase)**		**Treatment Phase**								**Follow Up Assessment and washout/Final visit (End of Study/Early Termination)**
Visit	**Visit 1 (at hospital)**	**Visit 2 [t] (at hospital)**	**Visit 3 (at hospital) Dose Escalation 1 [t]**	**Dose Escalation 2[t]**	**Visit 4 [t] (at hospital)**	**Dose Escalation 3[t]**	**Dose Escalation 4[t]**	**Dose Escalation 5[t]**	**Visit 5 (at hospital) Month 3**	**Visit 6 (at hospital) Month 6**	**Visit 7 (at hospital) Month 9**
**Dose Escalation Day**			**1**	**8**		**15**	**22**	**29**	**93**	**186**	**279**
**Visit Window**			(within 60 days of screening Visit 1 & 2)		*(within 3 days after dose escalation 2)*				(+/- 14 days)	(+/- 14 days)	**Early termination visit (+/- 30 days)
**Day**	-60	-60	1	8-14		15-21	22-28	29-186	93	186	279
Blood enzyme activity & ELISA antibody panels***	X				X				X	X	X

© 2020 Mullin S et al. *American Medical Association.* All rights reserved.

CSF enzyme activity & ELISA antibody panels***		X								X	X
Blood ambroxol collection***	X				X				X	X	
CSF ambroxol collection***		X								X	X
Urine Collection[c] (Up to 50 mL)***			X		X				X	X	X
Dosing, day 1 to 186 (inclusive)d (dosing, on-site, if applicable)			X		X				X	X	
Dispensing 3 months' supply IMP			X						X		
Collecting IMP packaging (pill count)									X	X	X
Cognitive/Questionnaire – MoCA[c], NMSS & NMS			X							X	
IMP compliance and dosing[d] instructions			X	X	X	X	X	X			
Adverse Events Review	X	X	X	X	X	X	X	X	X	X	X
Concomitant Medication Review	X	X	X	X	X	X	X	X	X	X	X

© 2020 Mullin S et al. *American Medical Association.* All rights reserved.

eTable 2. Summary Table of Biochemical Results Including *GBA1* and Non-*GBA1* Subgroups

Summary table of biochemical results including *GBA1* and non *GBA1* subgroups. (Participants who completed study).
Data are mean (SD). CSF – cerebrospinal fluid, SD – standard deviation, GCase – glucocerebrosidase enzyme

		Baseline	Day 29	Day 93	Day 186	Day 279 (washout)
**Number of participants (n)**		**Blood 18, CSF 17**	**Blood 18, CSF 17**	**Blood 18, CSF 17**	**Blood 18, CSF 17**	**Blood 18, CSF 6**
**Ambroxol, ng/ml**						
**CSF**	**All**	0 (0)	-	-	156 (53)	0(0)
	**GBA1**	0 (0)	-	-	173 (44)	0(0)
	**non GBA1**	0 (0)	-	-	141 (59)	0(0)
**Blood (serum)**	**All**	0 (0)	316 (196)	1084 (396)	1432 (570)	0(0)
	**GBA1**	0 (0)	348 (100)	1090 (223)	1568 (557)	0(0)
	**non GBA1**	0 (0)	299 (200)	1080 (507)	1324 (586)	0(0)
**Glucocerebrosidase (GCase) enzyme activity, nmol/ml/hr (CSF) or nmol/mg/hr (blood)**						
**CSF**	**All**	0.309 (0.153)	-	-	0.250 (0.142)	0.223 (0.131)
	**GBA1**	0.200 (0.090)	-	-	0.142 (0.055)	0.128 (0.044)
	**non GBA1**	0.405 (0.133)	-	-	0.345 (0.126)	0.318 (0.118)
**Blood (leucocytes)**	**All**	11.0 (5.2)	12.8 (4.9)	13.1 (4.8)	12.0 (5.2)	9.8 (4.5)
	**GBA1**	8.0 (4.3)	8.8 (2.9)	10.3 (3.2)	8.9 (3.1)	9.0 (3.1)
	**non GBA1**	13.4 (4.8)	16.0 (3.8)	15.6 (4.8)	14.5 (5.0)	10.6 (5.6)
**Glucocerebrosidase (GCase) protein levels, pmol/L**						
**CSF**	**All**	250 (47)	-	-	338 (104)	394 (66)
	**GBA1**	233 (41)	-	-	328 (100)	388 (101)
	**non GBA1**	264 (50)	-	-	347 (113)	399 (21)
**Alpha synuclein (A-SYN) , pg/ml**						
**CSF**	**All**	383 (103)	-	-	433 (117)	372 (46)
	**GBA1**	336 (68)	-	-	367 (74)	401 (23)
	**non GBA1**	425 (115)	-	-	492 (120)	342 (46)
**Blood (serum)**	**All**	20793 (9418)	19991 (7380)	24964 (9391)	23395 (9998)	22266 (12423)
	**GBA1**	22209 (8373)	19638 (8374)	26867 (12002)	23253 (10689)	21623 (10295)
	**non GBA1**	19660 (10472)	20273 (6940)	23441(6984)	23509 (9997)	22779 (14437)
**Tau, pg/ml**						
**CSF**	**All**	206 (59)	-	-	211 (63)	207 (53)
	**GBA1**	179 (43)	-	-	186 (51)	227 (59)
	**non GBA1**	230 (62)	-	-	233 (66)	188 (49)
**Blood (serum)**	**All**	1.00 (0.25)	0.84 (0.24)	0.88 (0.22)	0.80 (0.24)	0.66 (0.23)
	**GBA1**	0.96 (0.24)	0.93 (0.29)	0.88 (0.21)	0.79 (0.26)	0.64 (0.28)
	**non GBA1**	1.04 (0.26)	0.76 (0.17)	0.88 (0.24)	0.81 (0.24)	0.68 (0.20)
**Glucosylceramide, pg/ml**						
**CSF**	**All**	246 (83)	-	-	260 (80)	256 (103)
	**GBA1**	276 (65)	-	-	274 (53)	333 (49)
	**non GBA1**	219 (91)	-	-	247 (99)	178 (79)

© 2020 Mullin S et al. *American Medical Association.* All rights reserved.

eTable 3. Summary Table of Clinical Results Including *GBA1* and Non-*GBA1* Subgroups

Summary table of clinical results including *GBA1* and non *GBA1* subgroups. (Participants who completed study).

Data are mean (SD). Clinical markers markers are recorded in the off state. MDS UPDRS – Movement disorders society unified Parkinson disease ratings scale, MoCa – Montreal cognitive assessment, NMSS – Non motor symptoms scale, NMSQuest – Non motor symptoms questionnaire, SD – Standard deviation

	Baseline	Day 29	Day 93	Day 186	Day 279 (washout)
**Number of participants (n)**	18	18	18	18	18
**Movement disorders Society Unified Parkinson disease rating scale (MDS UPDRS) part I**					
**All**	10.3 (8.1)		10.5 (7.8)	10.1 (7.1)	9.4 (6.8)
GBA1	15.2 (9.7)		16.0 (8.6)	14.8 (7.0)	13.6 (7.8)
**non *GBA1***	6.3 (4.6)		6.1 (3.2)	6.4 (4.9)	6.1 (3.5)
**Movement disorders Society Unified Parkinson disease rating scale (MDS UPDRS) part II**					
**All**	15.2 (10.2)		14.9 (9.8)	15.6 (10.6)	14.4 (10.1)
GBA1	22.5 (10.5)		22.1 (10.0)	23.5 (9.1)	21.6 (10.3)
**non *GBA1***	9.4 (5.1)		9.1 (4.7)	8.6 (5.7)	8.6 (5.2)
**Movement disorders Society Unified Parkinson disease rating scale (MDS UPDRS) part III**					
**All**	31.1 (14.5)		27.2 (10.7)	24.3 (12.1)	31.9 (12.7)
GBA1	38.4 (15.9)		31.5 (11.1)	30.3 (13.1)	37.4 (11.4)
**non *GBA1***	24.3 (9.6)		23.7 (9.5)	19.0 (8.7)	27.5 (12.4)_
**Movement disorders Society Unified Parkinson disease rating scale (MDS UPDRS) part IV**					
**All**	4.9 (4.0)		5.1 (4.5)	4.3 (3.6)	5.3 (3.2)
GBA1	5.6 (5.7)		6.4 (5.8)	6.0 (4.2)	6.1 (3.6)
**non *GBA1***	4.4 (2.4)		4.1 (3.2)	3.1 (2.7)	4.7 (2.9)
**Movement disorders Society Unified Parkinson disease rating scale (MDS UPDRS) total**					
**All**	62.6 (32.2)		57.7 (27.6)	53.9 (30.3)	61.1 (26.7)
GBA1	82.4 (34.6)		76.0 (29.0)	73.8 (27.4)	78.8 (25.1)
**non *GBA1***	44.6 (16.8)		43.0 (15.5)	37.6 (19.9)	46.9 (18.7)
**Montreal Cognitive assessment (MoCa)**					
**All**	25.0 (4.8)	-	-	26.7 (4.0)	-
GBA1	23.6 (6.2)	-	-	25.6 (5.2)	-
**non *GBA1***	26.1 (3.2)	-	-	27.5 (2.7)	-
**Non Motor symptoms scale (NMSS)**					
**All**	49.3 (36.1)			60.8 (38.6)	-
GBA1	77.1 (38.0)			85.8 (37.9)	-
**non *GBA1***	27.0 (10.4)			40.8 (26.2)	-
**Non motor symptoms questionnaire (NMSQuest)**					
**All**	10.6 (6.0)	-	-	10.8 (6.0)	-
GBA1	14.2 95.8)	-	-	14.3 (5.0)	-
**non *GBA1***	7.6 (4.5)	-	-	7.9 (5.4)	-
**Safety information**					
**Weight, kg (all)**	83 (17)	83 (17)	82 (17)	82 (17)	83 (15)
**Mean arterial blood pressure, mmHg (all)**	90 (8)	88 (9)	90 (10)	90 (11)	95 (9)

© 2020 Mullin S et al. *American Medical Association*. All rights reserved.

eTable 4. Summary Table of CSF Biochemical Results Including *GBA1* and Non-*GBA1* Subgroups in Those Who Underwent 3 Lumbar Punctures

Summary table of CSF biochemical results including *GBA1* and non *GBA1* subgroups in those who underwent 3 lumbar punctures.
Data are mean (SD). CSF – cerebrospinal fluid, SD – standard deviation, GCase – glucocerebrosidase enzyme

		Baseline	Day 186	Day 279 (washout)
**Number of participants (n)**		**6**	**6**	**6**
**Ambroxol, ng/ml**				
**CSF**	**All**	0 (0)	191 (39)	0 (0)
	**GBA1**	0 (0)	187 (52)	0 (0)
	**non GBA1**	0 (0)	196 (34)	0 (0)
**Glucocerebrosidase (GCase) enzyme activity, nmol/ml/hr (CSF) or nmol/mg/hr (blood)**				
**CSF**	**All**	0.292 (0.135)	0.268 (0.159)	0.223 (0.130)
	**GBA1**	0.180 (0.068)	0.146 (0.075)	0.128 (0.044)
	**non GBA1**	0.403 (0.059)	0.389 (0.114)	0.318 (0.118)
**Glucocerebrosidase (GCase) protein levels, pmol/L**				
**CSF**	**All**	259 (50)	337 (102)	382 (67)
	**GBA1**	234 (38)	328 (101)	388 (102)
	**non GBA1**	277 (51)	345 (107)	378 (46)
**Alpha synuclein (A-SYN) , pg/ml**				
**CSF**	**All**	351 (73)	378 (65)	372 (46)
	**GBA1**	364 (103)	365 (72)	401 (23)
	**non GBA1**	338 (46)	391 (69)	342 (46)
**Tau, pg/ml**				
**CSF**	**All**	184 (50)	190 (37)	207 (53)
	**GBA1**	192 (49)	204 (34)	227 (59)
	**non GBA1**	176 (39)	175 (41)	188 (48)
**Glucosylceramide, pg/ml**				
**CSF**	**All**	224 (76)	243 (71)	255 (103)
	**GBA1**	283 (43)	287 (21)	333 (50)
	**non GBA1**	166 (48)	199 (78)	179 (78)

© 2020 Mullin S et al. *American Medical Association*. All rights reserved.

eTable 5. Summary Table of Adverse Events Felt to Be Possibly, Probably, or Definitely Related to Ambroxol

**Summary table of adverse events (AEs) felt to be possibly, probably or definitely related to ambroxol**			
AE	Number of AEs	Number of participants affected	Percentage of cohort who commenced IMP (n=20) affected
Nausea/Vomiting	5	5	20%
Rash	3	3	15%
Dizziness	5	3	15%
Reflux	2	2	10%
Diarrhoea	2	2	10%
Memory loss	1	1	5%
Runny Nose	1	1	5%

© 2020 Mullin S et al. *American Medical Association.* All rights reserved.

eTable 6. List of Recorded Adverse Events

**List of recorded adverse events**						
**Adverse event description**	**serious?**	**Related to ambroxol?**	**severity**	**Expected?**	**resolved?**	**occurred whilst taking ambroxol?**
Slightly raised serum urea	no	not related	mild	no	resolved	no
Slightly raised serum urate	no	not related	mild	no	resolved	no
Mild lymphopaenia	no	not related	mild	no	resolved	no
Ejection systolic murmur	no	not related	mild	no	resolved	no
Headache	no	not related	mild	no	resolved	no
Muscle stiffness in back	no	not related	mild	no	resolved	no
Fall out of bed	no	unlikely	mild	no	resolved	yes
Fall forward on to abdomen	no	unlikely	mild	no	resolved	yes
Bruise on left rib	no	unlikely	mild	no	resolved	yes
Unsteady on feet	no	unlikely	mild	no	unresolved	yes
Fall	no	unlikely	mild	no	resolved	yes
Cut on left thigh	no	unlikely	mild	no	resolved	yes
Productive cough	no	not related	mild	no	resolved	yes
Arguing with wife in sleep	no	not related	mild	no	resolved	yes
Fall	no	unlikely	mild	no	resolved	yes
Bruise Right arm	no	unlikely	mild	no	resolved	yes
Slurred speech	no	unlikely	mild	no	resolved	yes
Memory loss	no	unlikely	mild	no	resolved	yes
Fall	no	unlikely	mild	no	resolved	yes
Urinary incontinence	no	unlikely	mild	no	resolved	yes
Injury when paving fell on leg	no	not related	mild	no	resolved	yes
Chest infection	no	not related	mild	no	resolved	no
Headache post-LP	no	not related	severe	no	resolved	no
Nausea	no	not related	mild	no	resolved	no
Vomiting x1	no	not related	mild	no	resolved	no
Urinary Frequency	no	not related	mild	no	unresolved	no
Pain in Back	no	not related	mild	yes	resolved	no
Mitral valve leak	no	not related	mild	no	unresolved	yes
Shortness of breath	no	not related	mild	no	unresolved	yes
Neck Stiffness	no	not related	mild	no	resolved	yes
Worsening Dysarthria	no	not related	mild	no	resolved	yes
Worsening Dysphonia	no	not related	mild	no	resolved	yes
Fatigue	no	not related	mild	no	unresolved	yes
Fall	no	not related	mild	no	resolved	yes

© 2020 Mullin S et al. *American Medical Association*. All rights reserved.

Post LP headache	no	not related	mild	no	resolved	no
Backache LP Site	no	not related	mild	no	resolved	no
Headache post lumbar puncture	no	not related	mild	no	resolved	no
Diarrhoea	no	unlikely	mild	no	resolved	yes
Kidney stone pain	no	not related	mild	no	resolved	yes
Dry Mouth	no	unlikely	mild	no	resolved	yes
Constipation	no	unlikely	mild	no	resolved	yes
Dry eyes	no	unlikely	mild	no	resolved	yes
Slight kidney infection	no	not related	mild	no	resolved	yes
Pain in ribcage	no	not related	mild	no	resolved	yes
Aches and pains in ribcage	no	not related	mild	no	unresolved	yes
Pain in back	no	not related	mild	no	resolved	yes
Left arm muscle cramps	no	not related	mild	no	resolved	yes
Erythema at LP site	no	not related	mild	no	resolved	yes
Headache	no	not related	mild	no	resolved	no
Nausea	no	not related	mild	no	resolved	no
Headache	no	not related	mild	no	resolved	no
Mild Back Pain (Post LP)	no	not related	mild	no	resolved	no
Vomiting post LP	no	not related	mild	no	resolved	no
Headache post LP	no	not related	mild	no	resolved	no
Right arm muscular pain	no	not related	mild	no	resolved	yes
Rashes on chest,back and arms	no	probably	mild	yes	resolved	yes
memory loss	no	possibly	mild	no	resolved	yes
Weight loss	no	possibly	mild	no	unresolved	yes
Fall	no	unlikely	mild	no	resolved	yes
Fall	no	unlikely	mild	no	resolved	yes
Back pain around lumbar puncture site	no	not related	mild	no	resolved	yes
Slight Dryness to Face and Hands	no	not related	mild	no	resolved	yes
Slight Redness to Face and Hands	no	not related	mild	no	resolved	yes

Post LP headache	no	not related	mild	no	resolved	yes
Pneumonia	no	not related	mild	no	resolved	yes
Pain at LP Site	no	not related	mild	no	resolved	no
Anxiety pre LP	no	not related	mild	no	resolved	no
Bruise at LP Site (~2 inches)	no	not related	mild	no	resolved	no
Area of LP Erythema Site	no	not related	mild	no	resolved	yes
Acid Reflux	no	probably	mild	yes	resolved	yes
Loose Stools	no	probably	mild	yes	resolved	yes
Burning sensation to back of throat when taking IMP	no	probably	mild	yes	resolved	yes

© 2020 Mullin S et al. *American Medical Association*. All rights reserved.

Constipation	no	unlikely	mild	no	resolved	yes
Nausea	no	probably	mild	yes	resolved	yes
Anxiety	no	unlikely	mild	no	resolved	yes
Spasm in left lower back at night at LP site	no	not related	mild	no	unresolved	yes
Walking and balance problems	no	possibly	mild	no	resolved	yes
Mild discomfort/pain on LP site	no	not related	mild	no	resolved	no
Cut on finger	no	not related	mild	no	resolved	yes
Prolonged bleeding from finger cut	no	not related	mild	no	resolved	yes
Soreness around LP site	no	not related	mild	no	resolved	no
Bruising around LP site	no	not related	mild	no	resolved	no
Tendonitis	no	not related	mild	no	resolved	yes
Diarrhoea	no	unlikely	mild	no	resolved	yes
Rash at top of thighs	no	unlikely	mild	no	resolved	yes
Constipation	no	possibly	mild	no	resolved	yes
Nausea	no	possibly	mild	yes	resolved	yes
Cough	no	not related	mild	no	resolved	no
Sore throat	no	not related	mild	no	resolved	no
Back Pain (LP)	no	not related	mild	no	resolved	no
Itchy Red Area on Abdomen	no	possibly	mild	yes	resolved	yes
Headache	no	not related	mild	no	resolved	yes
Calcified area on left breast	no	not related	mild	no	unresolved	yes
Breast biopsy	no	not related	mild	no	resolved	yes
Breast biopsy	no	not related	mild	no	resolved	yes

Nausea	no	possibly	mild	yes	resolved	yes
Back Pain	no	not related	mild	no	resolved	yes
Knee pain	no	not related	mild	no	unresolved	yes
Fall	no	not related	mild	no	resolved	no
Coccyx pain	no	not related	mild	no	resolved	no
Muscular pain in neck	no	not related	mild	no	resolved	yes
Runny nose	no	possibly	moderate	no	resolved	yes
Headache	no	not related	mild	no	resolved	yes
Deterioration of gait	no	not related	mild	no	unresolved	no
Back ache at the site of LP	no	not related	mild	no	resolved	no
Fall	no	not related	mild	no	resolved	no
Trochanteric bursitis	no	not related	mild	no	unresolved	yes
Pain LP Site	no	not related	mild	no	resolved	yes
Lumbar Pain	no	not related	mild	no	unresolved	yes
Floating feeling/light headedness	no	possibly	mild	no	resolved	yes

© 2020 Mullin S et al. *American Medical Association*. All rights reserved.

Floating feeling/light headedness	no	possibly	mild	no	resolved	yes
Afternoon sweats and dizziness	no	possibly	mild	no	resolved	yes
Impaired swallow	no	unlikely	mild	no	unresolved	yes
Chest infection	no	unlikely	mild	no	resolved	yes
UTI	no	not related	mild	no	resolved	yes
Transient postural dizziness	no	not related	mild	no	resolved	yes
Transient postural dizziness	no	not related	mild	no	resolved	no
20 min period of increased shaking	no	not related	mild	no	resolved	no
Rapid eye movement sleep disorder	no	not related	mild	no	unresolved	yes
Feels 'spaced out' after first dosing. Lasts from 5 to 30 minutes.	no	possibly	mild	no	resolved	yes
Fall	no	unlikely	mild	no	resolved	yes
Cellulitis on left arm at biopsy site	no	not related	mild	no	resolved	yes
Vomiting (after 3 pints of beer)	no	definitely	mild	yes	resolved	yes
Nausea	no	definitely	mild	yes	resolved	yes
Vomiting	no	definitely	mild	yes	resolved	yes
Diarrhoea	no	possibly	mild	no	resolved	yes
Fall	no	unlikely	mild	no	resolved	yes
Tingling in left side of neck	no	not related	mild	no	resolved	yes
Vomiting	no	unlikely	mild	yes	resolved	yes
Falls	no	not related	mild	no	resolved	yes
Fall	no	not related	mild	no	resolved	yes
Slight headache	no	not related	mild	no	resolved	no
Soreness and mild pain at LP site	no	not related	mild	no	resolved	no
Whooshing in ears post Lp	no	not related	moderate	no	resolved	no
Severe headache post Lp	no	not related	severe	no	resolved	no
Nausea	no	not related	moderate	no	resolved	no
Non malignant skin growth	no	not related	mild	no	resolved	yes
Tender sacrum post LP	no	not related	mild	no	resolved	no
Headache post LP	no	not related	mild	no	resolved	no
Fatigue	no	not related	mild	no	resolved	no
UTI	no	not related	mild	no	resolved	yes
Dizziness	no	possibly	mild	no	resolved	yes
UTI, recurrent	no	not related	moderate	no	unresolved	yes
Back pain post LP	no	not related	mild	no	resolved	yes
Bruise post LP	no	not related	mild	no	resolved	yes
Increased coldness/freezing	no	unlikely	mild	no	resolved	yes
Headache post LP	no	not related	moderate	no	resolved	yes
Nausea post LP	no	not related	moderate	no	resolved	yes

© 2020 Mullin S et al. *American Medical Association*. All rights reserved.

Whooshing sound in ears post LP	no	not related	mild	no	resolved	yes
Headache post LP	no	not related	mild	no	resolved	no
Decreased hearing post LP	no	not related	mild	no	resolved	no
Nausea post LP	no	not related	mild	no	resolved	no
Headache, stated as 3/10 pain score	no	not related	mild	no	resolved	yes
Decreased hearing	no	not related	mild	no	resolved	yes
Decreased volume of voice	no	not related	mild	no	resolved	yes
Increased sleep	no	not related	mild	no	resolved	yes
Itching left forearm	no	not related	mild	no	resolved	yes
Erectile dysfunction	no	unlikely	mild	no	unresolved	yes
Viral illness	no	possibly	mild	no	resolved	yes
Headache post LP	no	not related	moderate	no	resolved	no
Back pain post LP	no	not related	mild	no	resolved	no
Nausea	no	not related	mild	no	resolved	no
Vomiting	no	not related	mild	no	resolved	no
Grinding of teeth	no	unlikely	mild	no	resolved	yes
Upper respiratory tract infection	no	not related	mild	no	resolved	yes
Umbilical hernia	no	not related	mild	no	unresolved	yes
Mild pain/ discomfort and soreness at the LP site	no	not related	mild	no	resolved	yes
Vomiting	no	unlikely	mild	no	resolved	yes
Increased tremors	no	unlikely	mild	no	resolved	yes
Vomiting	no	not related	mild	no	resolved	no
Post LP headache	no	not related	mild	no	resolved	no
Anaemia	no	not related	mild	no	unresolved	no
Increased tremors	no	not related	mild	no	resolved	no
Neck stiffness	no	not related	severe	no	resolved	yes
Headache	no	not related	severe	no	resolved	yes
Back pain	no	not related	mild	no	resolved	yes

© 2020 Mullin S et al. *American Medical Association*. All rights reserved.
